# Mitochondrial quality control in human health and disease

**DOI:** 10.1186/s40779-024-00536-5

**Published:** 2024-05-29

**Authors:** Bo-Hao Liu, Chen-Zhen Xu, Yi Liu, Zi-Long Lu, Ting-Lv Fu, Guo-Rui Li, Yu Deng, Guo-Qing Luo, Song Ding, Ning Li, Qing Geng

**Affiliations:** 1 Department of Thoracic Surgery, Renmin Hospital of Wuhan University, Wuhan, 430060 China; 2 Department of Thoracic Surgery, First Hospital of Jilin University, Changchun, 130021 China

**Keywords:** Mitochondrial quality control, Metabolism, Programmed cell death, Cancer, Cardiovascular disease, Metabolic disease, Nervous disease, Pulmonary disease, Kidney disease, Digestive system disease

## Abstract

Mitochondria, the most crucial energy-generating organelles in eukaryotic cells, play a pivotal role in regulating energy metabolism. However, their significance extends beyond this, as they are also indispensable in vital life processes such as cell proliferation, differentiation, immune responses, and redox balance. In response to various physiological signals or external stimuli, a sophisticated mitochondrial quality control (MQC) mechanism has evolved, encompassing key processes like mitochondrial biogenesis, mitochondrial dynamics, and mitophagy, which have garnered increasing attention from researchers to unveil their specific molecular mechanisms. In this review, we present a comprehensive summary of the primary mechanisms and functions of key regulators involved in major components of MQC. Furthermore, the critical physiological functions regulated by MQC and its diverse roles in the progression of various systemic diseases have been described in detail. We also discuss agonists or antagonists targeting MQC, aiming to explore potential therapeutic and research prospects by enhancing MQC to stabilize mitochondrial function.

## Background

Mitochondria are organelles found within eukaryotic cells that play a crucial role in energy production via oxidative phosphorylation (OXPHOS), generating adenosine triphosphate (ATP) [1]. Beyond their pivotal function as “the powerhouse of the cell”, mitochondria also contribute to the regulation of several cellular processes, including fatty acid oxidation (FAO), calcium buffering, phospholipid synthesis, iron-sulfur cluster biosynthesis, innate immune signaling, and cell death [2, 3]. Despite their importance in cell homeostasis, the process of OXPHOS generates reactive oxygen species (ROS) as a by-product, which can damage mitochondrial proteins, lipids, and DNA. Compounding this inherent challenge, mitochondria are also continually exposed to various environmental stressors [4], which augment their vulnerability to dysregulation. To combat this threat, eukaryotic cells have developed an intricate set of mitochondrial quality control (MQC) mechanisms to diligently monitor and maintain the integrity and function of the intracellular mitochondrial network.

MQC is a multifaceted group of processes that serve to safeguard the mitochondria against damage and prevent the accumulation of defective mitochondria. Those processes mainly involve three distinct mechanisms, namely mitochondrial biogenesis, mitochondrial dynamics (fusion and fission), and mitophagy [5]. Mitochondrial biogenesis is a tightly regulated process that involves the coordinated expression of nuclear and mitochondrial genes to bolster the size and quantity of mitochondria. Meanwhile, mitochondrial dynamics maintain mitochondrial health by continuously shifting between fusion and fission, enabling the elimination of unhealthy mitochondria to prevent their accumulation [6]. In cases where mild to moderate damage occurs, mitochondria can compensate for their loss of function by fusing with healthy mitochondria or undergoing fission to remove harmful components. However, when damage is too severe, mitophagy is required to selectively remove hypofunctional or damaged mitochondria [7]. The components of degraded mitochondria are subsequently renewed by protein and mitochondrial biogenesis. When the primary damage surpasses the capacity of MQC, the mitochondria unleash danger signals that can prompt cell-death decisions, ultimately resulting in tissue injury and potential organ failure. Therefore, the preservation of mitochondrial homeostasis is of paramount importance in ensuring cellular survival and overall organismal well-being.

In this review, we aim to clarify the current state of knowledge about MQC in human health and disease. We will delve into the intricate molecular mechanisms underlying mitochondrial biogenesis, mitochondrial dynamics, and mitophagy, along with their regulation. Moreover, we will thoroughly investigate the crucial role of MQC in a wide range of human diseases, including cancer, cardiovascular disease, metabolic disorders, neurological disorders, respiratory diseases, renal diseases, and digestive diseases. By deepening our comprehension of the complex mechanisms involved in MQC, we can devise innovative strategies to mitigate mitochondrial dysfunction and associated pathologies.

## Molecular regulations of MQC

**Fig. 1:**
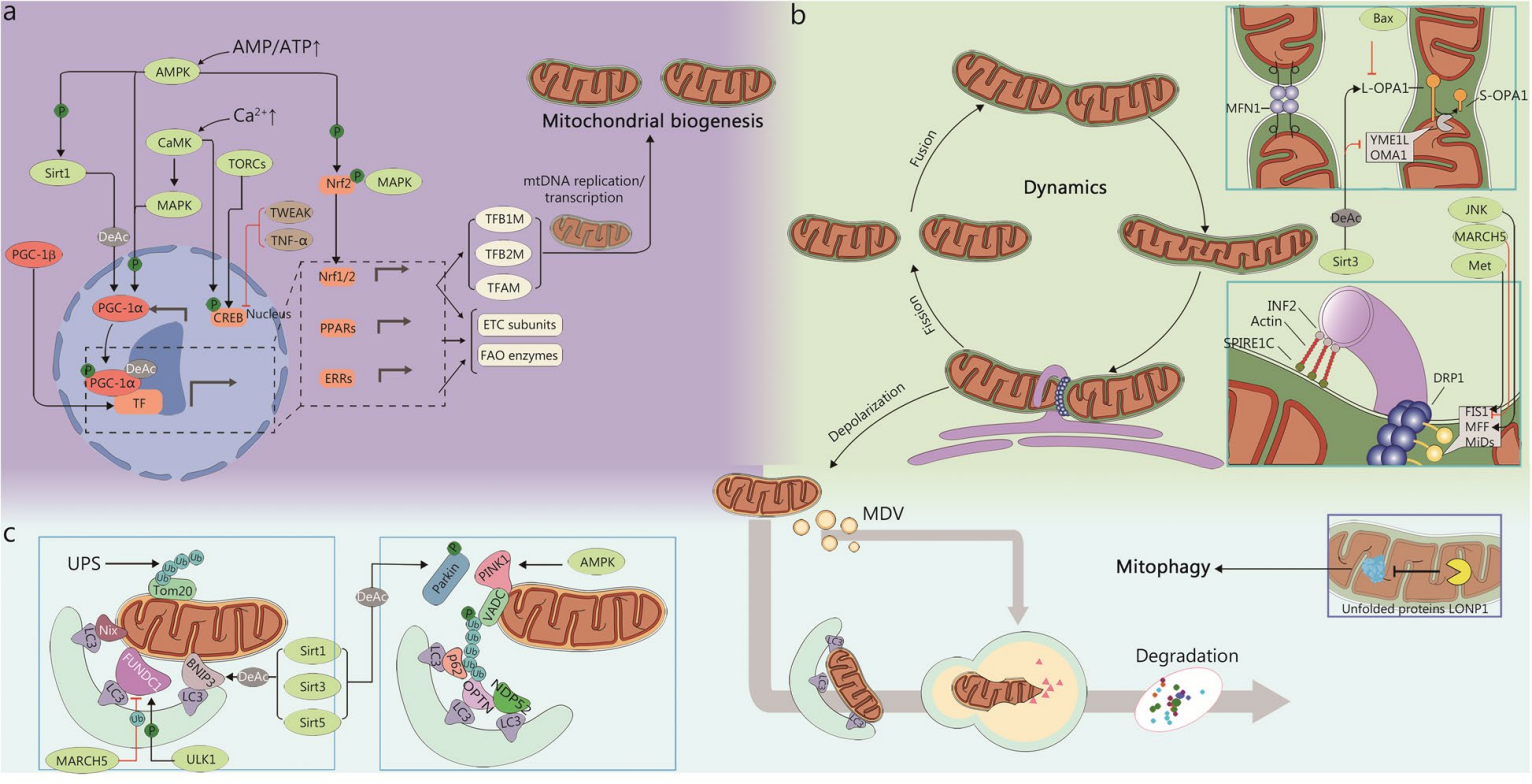
Molecular regulation of Mitochondrial quality control. **a** PGC-1α plays a central role in mitochondrial biogenesis. Several regulators, including AMPK, Sirts, and Ca^2+^, are involved in the regulation of PCG-1α expression and activity. In addition, PGC-1β is also involved in the mitochondrial biogenesis process. **b** Mitochondrial dynamics consists of fission and fusion. The fission-associated proteins (DRP1, FIS1, MFF, et al.) mediate the fission of mitochondria, a process receiving complex regulation by various factors such as endoplasmic reticulum and multiple kinases. The fusion event consists of mitochondrial outer membrane fusion mediated by MFNs and mitochondrial inner membrane fusion mediated by OPA1, similarly, they are subject to complex regulation at different stages of fusion. **c** The role of mitophagy is to remove damaged mitochondria promptly. There are two pathways of mitophagy, respectively, the PINK1/Parkin-dependent pathway and the PINK1/Parkin-independent pathway. Their common feature is the formation of autophagosomes that enclose damaged mitochondria and the complex regulation by multiple intracellular signals. Moreover, the presence of protein quality control systems in mitochondria removes misfolded mitochondrial proteins, and the accumulated unfolded proteins will promote mitophagy. AMPK AMP-activated protein kinaseBNIP3 BCL2 interacting protein 3, CaMK calcium/calmodulin-dependent protein kinase, DeAc deactylation, DRP1 dynamin-related protein 1, ETC electron transport chain, FAO fatty acid oxidation, FIS1 fission protein 1, FUNDC1 FUN14 domain containing 1, INF2 inverted formin 2, JNK Jun N-terminal kinase, LC3 microtubule associated protein 1 light chain 3, LONP1 lon protease 1, MAPK mitogen-activated protein kinases, MARCH5 membrane associated ring-CH-type finger 5, MDV mitochondria derived vesicle, Met MET proto-oncogene, MFF mitochondrial fission factor, MFN mitofusin, MiD mitochondrial dynamics, NDP52 nuclear dots protein 52, Nix NIP3-like protein X, Nrf2 nuclear factor E2-related factor 2, OMA1 OMA1 zinc metallopeptidase, OPA1 optic atrophy 1, OPTN optineurin, PGC-1α PPAR-γ coactivator-1α, PINK1 PTEN-induced kinase 1, Sirt1 sirtuin 1, TFAM mitochondrial transcription factor A, TFB1M mitochondrial transcription factors B1, TFB2M mitochondrial transcription factors B1, TNF-α tumor necrosis factor-α, Tom20 translocase of outer mitochondrial membrane 20, TORC transducer of regulated CREB (cAMP response element-binding protein), ULK1 unc-51 like autophagy activating kinase 1, UPS ubiquitin–proteasome system, VDAC voltage dependent anion channel, YME1L YME1 like 1 ATPase

Mitochondrial morphology, structures, mass, and even quantity are highly plastic in response to signals generated by normal physiological activities or pathological stimuli. To sustain cellular function and homeostasis, complex and highly dynamic MQC mechanisms have evolved under the regulation of the nuclear and mitochondrial genomes [8]. In general, the homeostasis of mitochondrial function and quality is achieved through two processes: the elimination of damaged mitochondria and the synthesis of new intact mitochondria [9]. Following, we review several important mechanisms of MQC and recent advances including mitochondrial biogenesis, mitochondrial dynamics, and mitophagy (Fig. 1).

### Mitochondrial biogenesis

Mitochondrial biogenesis was first found when the level of intracellular mitochondrial content was significantly increased during endurance training in the study of Holloszy et al. [10]. It can be understood as the growth and division of existing mitochondria in cells, which mainly requires the production of the mitochondrial inner and outer membrane, the replication of the mitochondrial genome, and the synthesis and assembly of mitochondrial proteins [11]. As stated, mitochondria are unique in that they have a separate extranuclear genome. Most of the mitochondrial proteins are encoded by nuclear genes, and mitochondrial DNA (mtDNA) is responsible for encoding 13 basic components of the electron transport chain (ETC), transfer RNA, and ribosomal RNA [12]. Therefore, a complex mechanism is necessary to coordinate the transcription and replication of the nuclear and mitochondrial genomes to guarantee dynamic and correct mitochondrial biogenesis, and a targeted delivery and assembly system is required for importing nuclear-encoded proteins to ensure proper mitochondrial morphology and function. The following text mainly elaborates on the regulatory factors and the latest upstream regulatory factors involved in the process of mitochondrial biogenesis.

#### Master transcriptional regulators of mitochondrial biogenesis

##### Peroxisome proliferator-activated receptor (PPAR)-γ coactivator-1α (PGC-1α)

It is the most important transcriptional cascade regulator in mitochondrial biogenesis, facilitating the transcription of mitochondrial proteins and the replication of mtDNA, which are key steps in mitochondrial biogenesis mentioned above [13]. PPARs are a superfamily of nuclear receptors that control the expression of related genes through the stimulation of different ligands to maintain intracellular energy homeostasis and manage oxidative stress levels [14]. PGC-1α was initially identified as a protein that interacts with PPARγ and enhances its transcriptional activity. It is noteworthy that upon binding to PPARs, particularly PPARα, PGC-1α further stimulates the generation of intracellular fatty acid transporters and the activation of mitochondrial FAO enzymes, leading to an upregulation of mitochondrial FAO process [15]. In recent years, many studies have shown that PGC-1α is not limited to activating PPARs but is the most crucial regulator in mitochondrial biogenesis, participating in the regulation of almost all processes related to mitochondrial biogenesis [16–18].

PGC-1α, as a potent transcriptional coactivator, interacts with various transcription factors without sequence-specific DNA binding to promote the expression of specific proteins, which is indispensable for mitochondrial biogenesis and regulation of cellular metabolism [19]. In tissues with abundant mitochondria and active oxidative metabolism, PGC-1α is expressed at high levels, and its expression is significantly increased by physiological signals such as exercise and fasting [20]. Upon signal stimulation, PGC-1α is activated and translocated into the nucleus, where it acts as a transcriptional coactivator to activate nuclear factor E2-related factor 2 (Nrf2) [21]. It not only directly promotes the expression of ROS scavenging enzymes, but also promotes the expression of mitochondrial-related proteins encoded by the nuclear genome induced by Nrf2. Nrf2, as well as its family member Nrf1, plays important roles in regulating mitochondrial gene replication and protein synthesis [22]. PGC-1α triggers the activation of Nrf1/2, which in turn directly boosts the expression of almost all components of the mitochondrial ETC. The PGC-1α-Nrf1 axis also stimulates the transcription of mitochondrial gene-encoded factors, including mitochondrial transcription factor B1 (TFB1M), TFB2M, and mitochondrial transcription factor A (TFAM), which mediate mtDNA replication and transcription [23]. Moreover, the expression of OXPHOS proteins also depends on PGC-1α promoting the binding of Nrf1/2 to the corresponding gene promoters, thus enhancing their transcription [24]. Additionally, the co-activation of PGC-1α and estrogen-related receptors (ERRs) is crucial for the process of mitochondrial biogenesis. The nuclear receptor family ERRs mainly includes ERRα, ERRβ, and ERRγ, which have significant homology with classic hormone receptors in the ligand-binding domain, but exert biological functions without requiring ligand. Currently, ERRα and ERRγ are known to play important regulatory roles in maintaining mitochondrial function [25]. Numerous studies have shown that PGC-1α activates ERRα to regulate the expression of key genes that are responsible for managing almost all energy transduction and ATP synthesis pathways in mitochondria, including FAO, the tricarboxylic acid (TCA) cycle, and OXPHOS [26, 27]. Furthermore, ERRγ is more likely to control ion channel proteins to affect the process of mitochondrial energy metabolism [28]. However, the function of ERRβ in mitochondrial biogenesis is still unknown. In summary, PGC-1α emerges as the pivotal regulator in orchestrating the transcriptional cascades of mitochondrial biogenesis and modulating the expression level or activation degree of PGC-1α represents a potent strategy to finely regulate the intricate process of mitochondrial biogenesis.

##### PGC-1β

PGC-1β and PGC-1α are part of the PGC-1 family and have very similar structures and functions [29]. However, PGC-1β is distributed differently among different cell types in the body. It acts as a coactivator for PPARs and ERRs, binding to nuclear receptors and transcription factors related to mitochondrial biogenesis. This process helps increase mitochondrial biogenesis and sustain the baseline function of mitochondrial [30]. For example, both PGC-1β and PGC-1α play a direct role in controlling mitochondrial biogenesis through Nrf1, which allows mitochondria to fulfill the energy needs of cells [31]. Additionally, PGC-1β maintains mitochondrial function and promotes mitochondrial biogenesis during proliferation, differentiation, and activation processes, particularly in M2 macrophages and osteoclasts that can proliferate [32]. Nevertheless, PGC-1β does not increase in brown adipose tissue in response to cold exposure or muscles during exercise. Overall, the role of PGC-1β in mitochondrial biogenesis is also crucial, separate from PGC-1α in this process.

#### Upstream regulatory targets for mitochondrial biogenesis

##### AMP-activated protein kinase (AMPK)

Changes in energy levels are an effective factor in stimulating mitochondrial biogenesis at an early stage. AMPK, a key player in connecting cellular metabolism and immune response, serves as a vital sensor for energy changes [33]. When a sudden drop in energy levels causes an increase in AMP, AMPK is triggered and activated to exert its phosphatase function, thereby directly stimulating PGC-1α and subsequently promoting the downstream Nrf1/2-TFAM pathway, leading to the upregulation of mitochondrial biogenesis [34, 35]. Additionally, upon activation, AMPK can also directly stimulate Nrf2 to initiate mitochondrial protein synthesis independently of PGC-1α [36]. Moreover, due to its wide range of functions, AMPK can activate some upstream factors of PGC-1α, such as calcium/calmodulin-dependent protein kinase (CaMK), cytochrome C, and the Sirt family [37–40]. A study has shown that AMPK can form a complex with PPARδ, even without exercise, enhancing mitochondrial energy metabolism and improving mass [41]. Currently, the use of drugs possessing AMPK-activating properties or upregulation of AMPK upstream-activated proteins offers a novel clinical strategy for enhancing mitochondrial biogenesis in the treatment and prevention of diseases.

##### CaMK

Ca^2+^ plays a crucial role in regulating mitochondrial biogenesis. When Ca^2+^ levels increase, it triggers a pathway involving CaMK activation that leads to the expression of PGC-1α and TFAM. This, along with enhanced binding of Nrfs to DNA, positively regulates mitochondrial biogenesis [42, 43]. The CaMK family can be divided into four categories, among which CaMK I, CaMKII, and CaMKIV have been widely studied concerning mitochondrial biogenesis. Their lower affinity and specificity for substrates enable them to exhibit diverse functions in vivo and act as enzymes that promote the phosphorylation of multiple intracellular proteins [44]. Studies have shown that CaMK upregulates and activates PGC-1α by promoting the phosphorylation of p38 mitogen-activated protein kinases (MAPK) and activating extracellular regulated protein kinase (ERK)/cAMP-response element-binding protein (CREB) pathways [43, 45, 46]. CaMKII is particularly important for promoting mitochondrial biogenesis in the heart and skeletal muscle, while CaMKIV, although significant, is not essential for this process [47]. Further research is needed to fully understand the role of CaMK in mitochondrial biogenesis.

##### Sirtuin 1 (Sirt1)

The NAD^+^/NADH ratio is seen as a key factor in modulating mitochondrial function. Sirt1, a well-researched “longevity gene”, is an NAD^+^-dependent histone deacetylase that is important for deacetylating mitochondrial proteins [48]. Research has shown that Sirt1 upregulates the activity of PGC-1α by promoting its deacetylation, thereby enhancing mitochondrial quality and biogenesis [48]. Additionally, the activity of Sirt1 can be influenced by AMPK as well [49].

##### Others

In addition to the factors mentioned above, there are still many other factors that can impact mitochondrial biogenesis by controlling the expression levels and activation of PGC-1α. Histone crotonylation, a type of post-translational modification, is one such factor that may regulate the expression of PGC-1α [50]. Therefore, enhancing the levels of the precursor of the substrate for histone crotonylation has been reported to boost mitochondrial biogenesis, particularly in cases of acute kidney injury [50]. The transcription of the *PGC-1α* gene is strongly activated by the coactivator transducer of Creb-related binding protein 1 (TORC1). Additionally, TORC2 and TORC3, other members of the TORC family, also play a role in activating PGC-1α transcription [51]. Furthermore, TORCs induce mitochondrial respiratory chain and TCA cycle processes. Similarly, there are several negative regulatory factors in the body as well. Members of the tumor necrosis factor (TNF) superfamily, such as TNF-like weak inducer of apoptosis (TWEAK) and TNF-α, reduce the expression of PGC-1α and mitochondrial biogenesis by activating nuclear factor-κB (NF-κB) [52]. Interestingly, research has indicated that the absence of receptor-interacting protein 140 significantly increases mitochondrial biogenesis and intracellular FAO [53]. The exploration of key targets regulating PGC-1α is becoming increasingly important in the study of mitochondrial biogenesis.

### Mitochondrial fusion and fission

The regulation of mitochondrial fusion and fission balance, also known as mitochondrial dynamics, is a pivotal process in MQC that allows cells to meet their metabolic demands, ensures the clearance of damaged organelles, and accomplishes self-renewal of mitochondria with minimal resources and energy requirements [54]. Mitochondrial fusion refers to the process in which two mitochondria merge to form a single mitochondrion [55]. This process is not limited to the relative positions of the two mitochondria and can occur at either the ends or the sides. Due to the double-membrane structure of mitochondria, outer membrane fusion, and inner membrane fusion are the two major events involved in mitochondrial fusion, and there may be a time difference between them [55]. Following the fusion of mitochondrial membranes, the mitochondrial matrix fusion occurs. However, it should be noted that the mitochondrial genome is located within the matrix, and its fusion is somewhat restricted [56]. Mitochondrial fission is when the mitochondrial membrane contracts triggered by the dynamin-related protein 1 (DRP1), leading to the division of the original mitochondrion into two independent organelles [57]. The dynamic changes in mitochondrial fusion and fission control the morphology of mitochondria, exchange of contents, and maintain the normal function of mitochondria at physiological or pathological levels [58]. In the regulation of mitochondrial dynamics, the following key molecules serve as the primary agents and regulatory targets for fusion or fission.

#### Regulators of mitochondrial fusion

##### Mitofusins (MFNs)

It is well-recognized that outer mitochondrial membrane (OMM) fusion is entirely reliant on MFN1 and MFN2 [59], which are highly homologous GTPases in mammals. They possess conserved GTPase domains that regulate GTP binding and hydrolysis, leading to conformational changes [60]. Consequently, both MFN1 and MFN2 exhibit remarkable similarities in terms of function and structure. Through their α-helical regions, MFNs form homodimers or heterodimers, thereby acting as bridges to facilitate OMM fusion. It has been observed that the knockout of MFNs followed by overexpression of either MFN1 or MFN2 through plasmid transfer reveals the greater significance of MFN1 in OMM fusion [61]. Apart from its core role in regulating mitochondrial fusion, MFN2 plays a more prominent role in mediating the contact between the endoplasmic reticulum (ER) and mitochondria [62]. Within the ER, MFN2 is accumulated in regions where the ER interacts with mitochondria, known as the mitochondria-associated ER membranes (MAMs) [63, 64]. By interacting with MFN1 or MFN2 located in the OMM, MFN2 facilitates fusion between mitochondria and the ER, which is essential for regulating Ca^2+^ levels and mitochondrial function through inter-organelle communication [65].

The MFNs undergo modifications at different sites in response to cellular stimuli or signals, resulting in distinct patterns of OMM fusion. Both MFN1 and MFN2 can be ubiquitinated by Parkin, a key protein involved in mitophagy [66, 67]. Following ubiquitination, these two proteins are degraded by the proteasomes, leading to the inhibition of mitochondrial fusion while promoting mitochondrial fission and mitophagy. Similarly, MARCH5 can ubiquitinate and inhibit the function of MFN1/2 [68, 69]. Additionally, phosphorylation modifications exert diverse effects on MFNs. Different members of the MAPK family promote the phosphorylation of MFN1/2. For instance, ERK phosphorylates Thr562 of MFN1, whereas Jun N-terminal kinase (JNK) upregulates the phosphorylation level of Ser27 in MFN2 [70, 71]. After being regulated by MAPK, MFN1/2 plays a negative regulatory role in mitochondrial fusion. Interestingly, it has been demonstrated that site-specific phosphorylation is a prerequisite for the ubiquitination and recognition of MFN2 by the proteasomes [72]. However, AMPK, as a cellular sensor for energy and metabolism, exhibits opposite effects on the function of phosphorylated MFN1/2 [73]. It upregulates the process of mitochondrial fusion, and the direct interaction between AMPK and MFN2 significantly impacts ER-mitochondria fusion [64]. In comparison to phosphorylation modifications, the role of deacetylation modifications of MFN1/2 in promoting mitochondrial fusion is more precisely defined [74, 75]. In summary, the fusion of OMM is a central initial step in mitochondrial fusion, and post-translational modifications of MFNs play an important role in maintaining mitochondrial morphology and function.

##### Optic atrophy 1 (OPA1)

The process of mitochondrial fusion is intricate and not always guaranteed to occur normally, particularly when it comes to the inner mitochondrial membrane (IMM) fusion following the OMM fusion, which adds to the complexity [55]. If the OMM fuses while the IMM fusion fails, the resulting complex will undergo fission and fragmentation. The primary regulator of IMM fusion is OPA1, a member of the GTPase family similar to MFNs. OPA1 is located on the inner side of the mitochondrial inner membrane and is anchored to the IMM through its unique N-terminal transmembrane domain [76]. During inner membrane fusion, GTPase activity of OPA1 is modulated and binds to GTP, facilitating conformational changes that enable polymerization and promote inner membrane fusion [77]. When performing its core function, OPA1 often requires MFN1 rather than MFN2 [78]. In cells, OPA1 exists in various forms distinguished by length. Among them, the long isoform of OPA1, known as L-OPA1, plays a pivotal role in promoting IMM fusion and maintaining mitochondrial network integrity. The cleavage of L-OPA1 is primarily carried out by two proteases on the IMM, i-AAA protease YME1L, and metallopeptidase OMA1, in response to stress or metabolic changes [79]. Excessive accumulation of cleaved fragments, namely S-OPA1, results in mitochondrial fission or even fragmentation. Therefore, maintaining a balanced level between L-OPA1 and S-OPA1 is essential for regulating mitochondrial dynamics [80].

OPA1 is a key factor in maintaining the morphology and function of mitochondria. Its deficiency not only inhibits mitochondrial fusion but also significantly impacts the remodeling of mitochondrial cristae. It has been reported that upon activation of the apoptotic B-cell leukemia/lymphoma 2 (BCL2) family members Bax/Bak, there is an elevation in OPA1 phosphorylation levels, leading to its dissociation and interference with the formation and remodeling of mitochondrial inner membrane cristae, thereby affecting mitochondrial energy metabolism processes [81]. The regulation of OPA1 in cells is complex and involves its transcriptional level, activation state, and proteolytic cleavage. Mitochondrial factors like PGC-1α and TFAM can control the transcriptional level of OPA1 [82]. Furthermore, transcription factors related to immune regulation, such as NF-κB and signal transducer and activator of transcription 3 (STAT3), can also participate in OPA1 regulation in response to cellular environmental changes or inflammatory stimulation [83]. Concerning OPA1 modifications, research suggests that phosphorylation of OPA1 negatively impacts mitochondrial fusion, with upstream regulatory factors including Bax. Additionally, Sirt3 has been found to promote OPA1 deacetylation, enhancing its GTPase activity, which in turn facilitates OPA1 oligomerization and promotes mitochondrial fusion in response to cellular stimuli [84]. Sirt3 also aids in the deacetylation of YME1L, inhibiting its degradation activity and reducing the accumulation of S-OPA1, thereby promoting mitochondrial fusion through an alternative pathway [85].

#### Regulators of mitochondrial fission

##### DRP1

The dynamin-like GTPase DRP1 plays a crucial role in regulating mitochondrial fission. While primarily located in the cytoplasm during its inactive state, DRP1 is translocated to the OMM upon receiving signals to initiate mitochondrial fission. Here, it utilizes its GTPase activity by hydrolyzing GTP to obtain the necessary energy [86]. Upon activation, DRP1 undergoes conformational changes facilitated by interactions with other DRP1 molecules within the same mitochondrion, forming ring-shaped or helical aggregates that divide the intermembrane space between the inner and outer mitochondrial membranes [56]. Subsequent contraction of these DRP1 aggregates results in the division of mitochondria into two independent daughter mitochondria, which is a vital process for maintaining mitochondrial quantity and morphology [87].

Due to the lack of a domain directly binding to membrane phospholipids, the localization and activation of DRP1 in mitochondria rely on receptor proteins and recruitment factors on the mitochondrial surface [88]. Fission protein 1 (FIS1) interacts closely with the OMM through its C-terminal transmembrane domain, positioning itself on the OMM and recruiting DRP1 [89]. In yeast, the absence of FIS1 leads to the loss of DRP1’s function in promoting mitochondrial fission [89]. In mammals, although FIS1 is involved in promoting mitochondrial fission, there are also other proteins such as mitochondrial fission factor (MFF), mitochondrial dynamics protein of 49 kD (MiD49), and MiD51 that partially overlap with FIS1 in function and form complexes with DRP1 to regulate its function [90, 91]. MFF shares similar localization and function with FIS1, recruiting high-oligomeric forms of DRP1 and stimulating its GTPase activity [92]. Experimental evidence shows that overexpression of MFF exacerbates mitochondrial fission, while its absence does not significantly affect mitochondrial dynamics. The MiDs (MiD49 and MiD51) possess nucleotide transferase domains, with MiD51 requiring ADP as a cofactor to stimulate DRP1 oligomerization and GTPase activity [93]. The functions of MiDs are distinct from MFF, and in cells lacking MiD49/51, there is limited recruitment of DRP1, with minimal release of mitochondrial contents like cytochrome C [93]. Notably, MiDs exhibit a pro-fission role at low expression levels, but upon overexpression, mitochondria tend to elongate rather than undergo fission in response to stimuli [94, 95], possibly due to limited space for DRP1 contraction and aggregation, necessitating further investigation into the underlying mechanisms.

Additionally, the level of DRP1 modification significantly influences its function. During the early stages of cellular proliferation, Cyclin B/cyclin-dependent kinase 1 (CDK1) enhances the phosphorylation levels of DRP1 at Ser585 and Ser616, thereby increasing the GTPase activity of DRP1 and promoting mitochondrial fission [96, 97]. To interact with mitochondria, DRP1 must bind to specific receptors. Research indicates that phosphorylation of the Ser637 site of DRP1 inhibits its binding to mitochondrial receptors [96]. CaMK has been identified as a drive of mitochondrial fission through the promotion of dephosphorylation at the Ser637 site, underscoring the involvement of Ca^2+^ in mitochondrial dynamics [98]. However, protein kinase A (PKA) acts in opposition by elevating phosphorylation at the same site, thereby inhibiting the GTPase activity of DRP1 [99]. Moreover, the mitochondrial phosphatase phosphoglycerate mutase 5 (PGAM5) promotes mitochondrial fission and exacerbates cell death by recruiting and regulating dephosphorylation levels of DRP1 at Ser637 [100]. In various cell types, phosphorylation modifications of DRP1 exhibit distinct functional roles. For example, in neuronal cells, phosphatase and tensin homolog (PTEN)-induced kinase 1 (PINK1) enhances Ser616 phosphorylation of DRP1 to facilitate synaptic development, while in macrophages, STAT2 increases mitochondrial quality and modulates the pro-inflammatory phenotype by promoting phosphorylation of DRP1 at the same site [101, 102]. SUMOylation refers to a post-translational modification in which small ubiquitin-like modifier (SUMO) proteins are attached to specific sites of a target protein, altering its conformation and activity without inducing degradation [103]. The key enzyme responsible for SUMOylation modification of DRP1 is the mitochondrial anchoring protein ligase MAPL (mitochondrial E3 ubiquitin protein ligase 1, MUL1) [104]. Overexpression of MAPL promotes SUMOylation modification of DRP1, increasing its stability and activity, and positively regulating the process of mitochondrial fission, which is an important process in programmed cell death (PCD) [104]. Conversely, SUMO-specific peptidase 5 (SENP5), acting as a SUMO protease, inhibits OMM fission by cleaving SUMO1 from DRP1 [105]. Loss of SENP5 leads to uncontrolled fragmentation of mitochondria and even cell cycle arrest. However, a recent study has shown that two isoforms of SENP5, SENP5L, and SENP5S, counteract each other in the SUMOylation modification of DRP1 [105]. The balance between the two isoforms influences mitochondrial dynamics directionally; however, further investigation is required to elucidate this specific regulatory mechanism.

The role of ER in the initial stage of mitochondrial fission is a crucial aspect that warrants attention. DRP1 is not yet fully recruited and activated during the preparatory phase of mitochondrial fission. In the presence of mtDNA and Ca^2+^, the ER envelops the mitochondria, inducing preconstruction and reducing their average diameter to approximately 150 nm [106]. This process involves the interaction between inverted formin protein 2 (INF2) associated with the ER and SPIRE1C anchored to the mitochondria, which facilitates actin polymerization [107]. Simultaneously, at the interface where the ER contacts the mitochondria, known as the mitochondrial-associated membrane, oligomeric forms of DRP1 are recruited by MFF or MiDs to form a ring-like structure that initiates mitochondrial membrane fission [108]. Furthermore, the activation of DRP1 by certain cytoskeletal proteins has been confirmed, highlighting the significant role of the ER in regulating mitochondrial dynamics.

##### Adapters of DRP1

As previously discussed, although the adapter proteins of DRP1 exhibit overlapping functions, their post-translational modifications also play a significant role in mitochondrial fission. For instance, the tyrosine kinase Met promotes the phosphorylation of FIS1, facilitating its binding to DRP1 and enhancing mitochondrial fission [109]. Conversely, MARCH5 targets FIS1 for ubiquitination, resulting in its degradation by proteases and inhibiting its overall levels, consequently attenuating mitochondrial fission [110]. Similarly, in response to cellular changes, JNK can elevate the phosphorylation levels of MFF, thereby augmenting the cellular response to mitochondrial fission [111].

##### S-OPA1

The role of L-OPA1, located in the mitochondrial inner membrane, in mitochondrial fusion is well known. However, the fragmented form of OPA1, termed S-OPA1, has recently been found to exert significant effects on mitochondrial fission. S-OPA1 is generated through the cleavage of L-OPA1 by mitochondrial enzymes like OMA1, YME1L, or MPP, and remains soluble in the intermembrane space of the mitochondria [112]. Although different cleavage sites result in varying lengths of S-OPA1 structures, the accumulation of any isoform of S-OPA1 positively influences mitochondrial fission. Elevated cellular stress signals or alterations in metabolic conditions, particularly abnormalities in OXPHOS, enhance the activity of OMA1 or YME1L, accelerating the accumulation of S-OPA1 [113]. In normal physiological conditions, S-OPA1 synergistically interacts with L-OPA1, but excessive accumulation of S-OPA1 yields contrary effects [79, 114]. The specific mechanisms underlying the limitation of mitochondrial fusion and the promotion of mitochondrial fission due to the accumulation of S-OPA1 remain incompletely elucidated. Nonetheless, a prevailing view among researchers is that S-OPA1 disrupts the stability of the mitochondrial contact sites and cristae junction organizing system complex, as well as the function of OMM-IMM contact, thereby facilitating IMM fission [115]. Furthermore, the decline in the proportion of L-OPA1 significantly impedes mitochondrial fusion. Consequently, there exists substantial scope for further exploration into the ramifications of distinct OPA1 forms on mitochondrial dynamics.

### Mitophagy

Autophagy is a highly conserved cellular process in eukaryotic cells. It involves the recycling of cellular components through the formation of autophagosomes, which engulf and degrade cellular contents in lysosomes [116]. This process maintains a balance between biosynthesis and degradation of cellular organelles and facilitates the selective turnover of surplus or dysfunctional cellular components to meet energy and macromolecular demands during cellular renewal [117]. Autophagy can be classified into three main types based on the pathways through which the degraded components reach lysosomes: macroautophagy, microautophagy, and chaperone-mediated autophagy [118]. Among these, macroautophagy is the most important and prevalent type and is characterized by the non-specific degradation of engulfed materials. When cells are subjected to external stimuli such as damage, nutrient deprivation, or oxidative stress, mitochondria may be damaged, depolarized, and lose their membrane potential, which triggers a selective macroautophagy mechanism that degrades mitochondria, known as mitophagy [9, 119]. The damaged mitochondria are engulfed and sequestered into autophagosomes, forming mitochondrial autophagosomes, which then fuse with lysosomes for degradation, ensuring the balance of mitochondrial content and energy [7]. Mitophagy can occur in various cell types. In addition to clearing dysfunctional mitochondria and reducing cellular damage signals, excessive mitophagy can result in the accumulation of cytotoxic substances and metabolic disturbances. Mitophagy can be divided into two categories based on initiating factors and mitophagosome formation mechanism: ubiquitin-dependent pathway and ubiquitin-independent pathway. In the ubiquitin-independent pathway, specific receptor proteins such as BCL2 interacting protein 3 (BNIP3)/ BCL2 interacting protein 3 like (BNIP3L), FUNDC1, and AMBRA1 recruit LC3 to facilitate mitochondria degradation [7]. The remainder of this section provides a comprehensive overview of the main pathways and regulatory factors involved in mitophagy.

#### Main pathways of mitophagy

##### PINK1/Parkin

Ubiquitin-dependent process of mitophagy is mediated by Parkin, an E3 ubiquitin ligase that regulates the ubiquitination of mitochondrial proteins. Similar to other RING-between-RING E3 ligases, Parkin forms a ubiquitin-thioester intermediate at the active site Cys431 and transfers ubiquitin to substrates [120]. However, Parkin exhibits low specificity in substrate selection, allowing it to modify multiple proteins on the OMM to drive mitophagy. Notably, numerous OMM proteins ubiquitinated by Parkin are extensively degraded by the ubiquitin–proteasome system (UPS) before mitophagy, including MFN1, MFN2, FIS1, and Tom20, which are essential for subsequent PINK1/Parkin-mediated mitophagy [121–123]. These ubiquitinated and degraded OMM proteins, such as MFN2, can drive mitophagy by disrupting mitochondria-ER coupling [124]. The degradation of specific OMM proteins can also result in OMM rupture and the subsequent degradation of IMM and mitochondrial matrix proteins, which may also be in part removed by the lysosome [125, 126]. The acquisition of Parkin enzymatic activity relies on its ubiquitin-like domain [127]. In the inactive state, the inhibitory conformation resulting from interactions among Parkin’s unique domains prevents downstream target ubiquitination. Upon activation by PINK1, the ubiquitin-like domain undergoes phosphorylation at the Ser65 site, altering Parkin’s conformation and exposing the active site Cys431, thereby enabling its ubiquitin ligase activity, along with the phosphorylated ubiquitin by PINK1 activation at Ser65 [127]. PINK1’s activation of Parkin extends beyond this role. PINK1 phosphorylates the ubiquitin moieties on mitochondrial surface proteins, generating phosphorylated ubiquitin, which recruits Parkin to the mitochondria [127]. On depolarized mitochondria, Parkin facilitates further substrate ubiquitination, which promotes the phosphorylation activity of PINK1 and forms a positive feedback loop [128]. These two phosphorylation events establish the essential role of PINK1 in Parkin-mediated mitophagy.

Once Parkin is fully functional, the presence of autophagy-related adaptor proteins ensures the encapsulation of mitochondria by autophagosomes, with LC3 coating their surfaces [129]. Among them, the most important is Sequestosome-1 (p62/SQSTM1), which contains a ubiquitin-binding domain to recognize ubiquitin chains on mitochondria [130]. It also possesses an LC3-interacting region (LIR) to recruit and bind to the autophagosome, thus selectively bridging ubiquitinated proteins to the autophagosome. Research has shown that the absence of p62 does not affect the activation of PINK1/Parkin but does impair the final clearance of targeted mitochondria [67]. Similar autophagy receptor proteins include optineurin (OPTN), NDP52, NBR1, and TAX1BP1 [131]. Among them, OPTN and NDP52 can directly bind ubiquitin-tagged mitochondria to the autophagosome through their LC3-interacting regions, even without the involvement of Parkin [132].

The fusion of autophagosomes with lysosomes is the final step, in which autolysosomes are formed to degrade damaged mitochondria [133]. Pleckstrin homology domain-containing protein family member 1 (PLEKHM1) contributes to the initiation of autophagosome-lysosome fusion by attaching to the LC3, Rab7, and HOPS complex [134]. Furthermore, Atg8 family members, especially the GABARAP subfamily, can recruit PLEKHM1 to promote autophagosome-lysosome fusion during PINK1/Parkin-mediated mitophagy [7, 129].

##### BNIP3 and BNIP3L (Nix)

Some receptor proteins on the mitochondria can directly bind to LC3 through their own LIR without the need for Parkin-mediated ubiquitination to initiate mitophagy. In mammals, BNIP3/BNIP3L share a certain degree of homology and belong to a subfamily of the BCL2 family [135]. BNIP3 was initially identified as a pro-apoptotic factor that regulates the function of BCL2, with its C-terminus anchoring to the OMM through a transmembrane domain, and its N-terminus being the region where it mainly exerts its pro-mitophagy function [136]. The N-terminus of BNIP3 contains a BH3 domain and a LIR domain surrounded by two serine residues (Ser17 and Ser24), of which phosphorylation of Ser17 is crucial for the binding of BNIP3 to LC3B [137]. BNIP3L, also known as Nix, has a highly similar structure to BNIP3, including the distribution of the C- and N-terminus, and both require self-dimerization for pro-mitophagy function. BNIP3L was initially reported to be involved in mitochondrial clearance during erythrocyte maturation, but subsequent studies have revealed its role in various cell types [138]. Phosphorylation of Ser34 and Ser35 near the LIR domain of BNIP3L stabilizes its interaction with LC3 [139]. Under hypoxic conditions, Ser81 of BNIP3L is activated by hypoxia-inducible factor-1α (HIF-1α), which promotes its binding to LC3 [140]. In this case, ubiquitination of specific OMM proteins and degradation by UPS affect mitochondria fragmentation, representing the upstream of BNIP3-dependent mitophagy [123]. In abnormal cellular metabolic environments with increased energy demands, BNIP3L promptly clears dysfunctional mitochondria to accelerate mitochondrial recovery and enhance OXPHOS efficiency, which requires the recruitment of the small GTPase RHEB to the OMM for upregulating BNIP3 function [141, 142].

Due to the sequence similarity between BNIP3 and BNIP3L, the expression of BNIP3 can restore the mitochondrial clearance rate in reticulocytes lacking BNIP3L, and the knockout of BNIP3 can induce upregulation of BNIP3L expression, but this upregulation cannot compensate for the reduction in mitophagy caused by BNIP3 knockout [143]. Recent findings seem to have indicated the roles of BNIP3 and BNIP3L in PINK1/Parkin-mediated mitophagy. BNIP3L is ubiquitinated by Parkin, which promotes the targeting of the selective autophagy receptor NBR1 [144]. BNIP3 interacts with PINK1, promoting the accumulation of PINK1 on the OMM, leading to the translocation of Parkin to the mitochondria [145].

##### FUN14 domain containing 1 (FUNDC1)

FUNDC1 is exclusively localized to the OMM and serves as a receptor mediating mitophagy in response to hypoxic stress in mammals [146]. FUNDC1 has three transmembrane domains and a LIR domain at its N-terminus [146]. The pro-mitophagy function of FUNDC1 is regulated by phosphorylation and dephosphorylation of residues Ser13 and Tyr18 near its LIR domain [147]. Under resting conditions, FUNDC1 is phosphorylated at Tyr18 by SRC (SRC proto-oncogene, non-receptor tyrosine kinase) and at Ser13 by casein kinase II, inhibiting its interaction with LC3 under normoxic conditions [148, 149]. However, during hypoxic conditions, the deactivation of SRC weakens Tyr phosphorylation, PGAM5 dephosphorylates Ser13, and Unc-51-like autophagy activating kinase 1(ULK1) activates Ser17 [149, 150]. These events collectively enhance the interaction between FUNDC1 and LC3, thus promoting mitophagy.

##### Others

There are several other mitophagy receptor proteins present on mitochondria that participate in binding with autophagosomes to promote mitophagy. Similar to BNIP3 and BNIP3L, AMBRA1 contains a BH3 domain that facilitates its interaction with BCL2 family proteins [151]. Early in mitophagy initiation, AMBRA1 is released from mitochondrial BCL2 and interacts with BECN1 to participate in the autophagy process [152]. Moreover, AMBRA1 synergistically interacts with the E3 ubiquitin ligase HUWE1 to recruit LC3B indirectly and induce mitophagy in response to mitochondrial depolarization [151]. BCL2 like 13 (BCL2L13), originally described as a pro-apoptotic member of the BCL2 protein family, contains all conserved BH domains: BH1, BH2, BH3, and BH4, as well as two LIR domains [153]. While the homolog of yeast Atg32 has not been identified in mammals, BCL2L13 can induce mitophagy in cells lacking Atg32, leading some researchers to propose that BCL2L13 is a functional counterpart of mammalian Atg32 [153]. BCL2L13 functions independently of Parkin-mediated mitophagy, and phosphorylation at Ser272 increases the binding of BCL2L13 to LC3 [154]. Additionally, BCL2L13 can regulate mitochondrial morphology by mediating mitochondrial fission through its four BH domains [155].

Under mild stress conditions, mitochondria-derived vesicle (MDV) formation serves as an alternative mechanism, allowing cells to degrade aberrant mitochondrial proteins and delicately regulates the mitochondrial proteome [156, 157]. Different MDV subpopulations are separated by single or double membranes composed of OMM or both OMM and IMM, which contain different cargoes and are selectively transported to multivesicular bodies or lysosomes for degradation [158–160]. PINK1 and Parkin appear to be involved in a subset of MDV biogenesis induced by oxidative stress [159]. In this process, syntaxin-17 is recruited to Parkin-dependent mitochondria-derived vesicles (MDVs) and subsequently forms a soluble NSF attachment protein receptor (SNARE) complex with SNAP29 and VAMP7 to facilitate the late endosomes/lysosomes fusion [161, 162]. The formation and degradation of Parkin-dependent MDVs occur independently of autophagy-related proteins and are supported by the silence of Atg5 and beclin-1, separating this process from mitophagy [159]. Conversely, PINK1 and Parkin have been shown to play a negative role in inflammation-induced MDV formation [163]. Previous studies have demonstrated that neither PINK1 nor Parkin are essential for the formation of steady-state MDVs [164]. This subset of MDVs is generated through the protrusion of the mitochondrial membrane facilitated by microtubule-associated motor proteins MIRO1 and MIRO2 (MIRO1/2) and subsequently released via DRP1 recruitment mediated by MID49, MID51, and MFF [164, 165].

Emerging findings on mitochondrial remodeling and MQC mechanisms manifest as a distinct mitochondrial structural transformation, called mitochondrial spheroids [166, 167]. Reportedly, oxidative stress can induce the formation of mitochondrial spheroids, and those with ring or cup-like morphology can surround cytosolic components, including ER and other mitochondria [167–169]. MFN1 and MFN2 are required for the generation of mitochondrial spheroids [170]. Parkin lies at the crossroads of mitochondrial spheroids and mitophagy. Parkin suppresses mitochondrial spheroid formation by triggering MFN1 and MFN2 proteasomal degradation. Mitochondrial spheroids are positive for lysosome proteins, implying a possible capacity to degrade their enwrapped contents, while definitive evidence is yet lacking [171]. Given the current findings, mitochondrial spheroids are considered to represent a protective alternative strategy for maintaining mitochondrial homeostasis when PINK1/Parkin-related mitophagy is hindered [169, 170].

#### Regulators of mitophagy

##### ULK1

As a functional homolog of yeast Atg1 in mammalian cells, it forms a complex with Atg13 and RB1CC1 to initiate autophagy [172]. ULK1 functions as a phosphatase in mitophagy by promoting FUNDC1 Ser17 phosphorylation to increase its LC3 binding ability, and upregulating ACT domain Ser108 phosphorylation in Parkin to activate it in an alternative pathway to PINK1 [173]. BCL2L13-mediated mitophagy depends on the interaction with ULK1 to localize the autophagy initiation complex to the mitochondria [174].

##### AMPK

It plays a positive regulatory role in autophagy by inhibiting the phosphorylation of the negative regulator mTOR and directly upregulating the activity of ULK1 [172]. Additionally, the activation of AMPK promotes the translocation of PINK1 from the cytoplasm to the mitochondria, thereby facilitating mitophagy [175]. AMPK can also activate mitochondrial fission protein FIS1 to upregulate the process of mitophagy [176].

##### MARCH5

As an E3 ubiquitin ligase, MARCH5 inhibits mitochondrial fusion by increasing the ubiquitination of MFN1/2. Research has revealed that MARCH5-mediated ubiquitination of FUNDC1 at Lys119 decreases the levels of FUNDC1 protein in OMM [177]. Specifically, FUNDC1 expression is reduced via ubiquitin-proteasome-dependent degradation during hypoxia, thereby suppressing mitophagy.

##### Sirts

Sirt family is a subclass of the histone deacetylase family that relies on NAD^+^ levels to maintain activity. Among them, Sirt1 promotes mitophagy by upregulating the transcription factor forkhead box O3 (FOXO3), which in turn promotes BNIP3 expression [178]. Additionally, Sirt1 has been reported to directly upregulate the protein levels of PINK1 and Parkin [179]. Sirt3 regulates BNIP3 activity through the ERK-CREB signaling pathway and promotes mitophagy by FOXO3a-mediated PINK1/Parkin pathway [180–182]. Sirt5 also upregulates the expression levels of mitophagy markers PINK1, Parkin, and even BNIP3 [183]. However, the specific mechanisms still require further exploration.

##### Human Lon protease 1 (LONP1)

The protein LONP1 localizes within the mitochondrial matrix, and plays a crucial role in degrading misfolded proteins, thereby preventing protein aggregation. This function is essential for maintaining the mitochondrial proteostasis [1, 184]. Research has demonstrated that LONP1 specifically targets unfolded proteins in the mitochondrial matrix, and their accumulation promotes PINK1/Parkin-mediated mitophagy in a manner independent of mitochondrial depolarization [185].

## Physiological roles of MQC

**Fig. 2:**
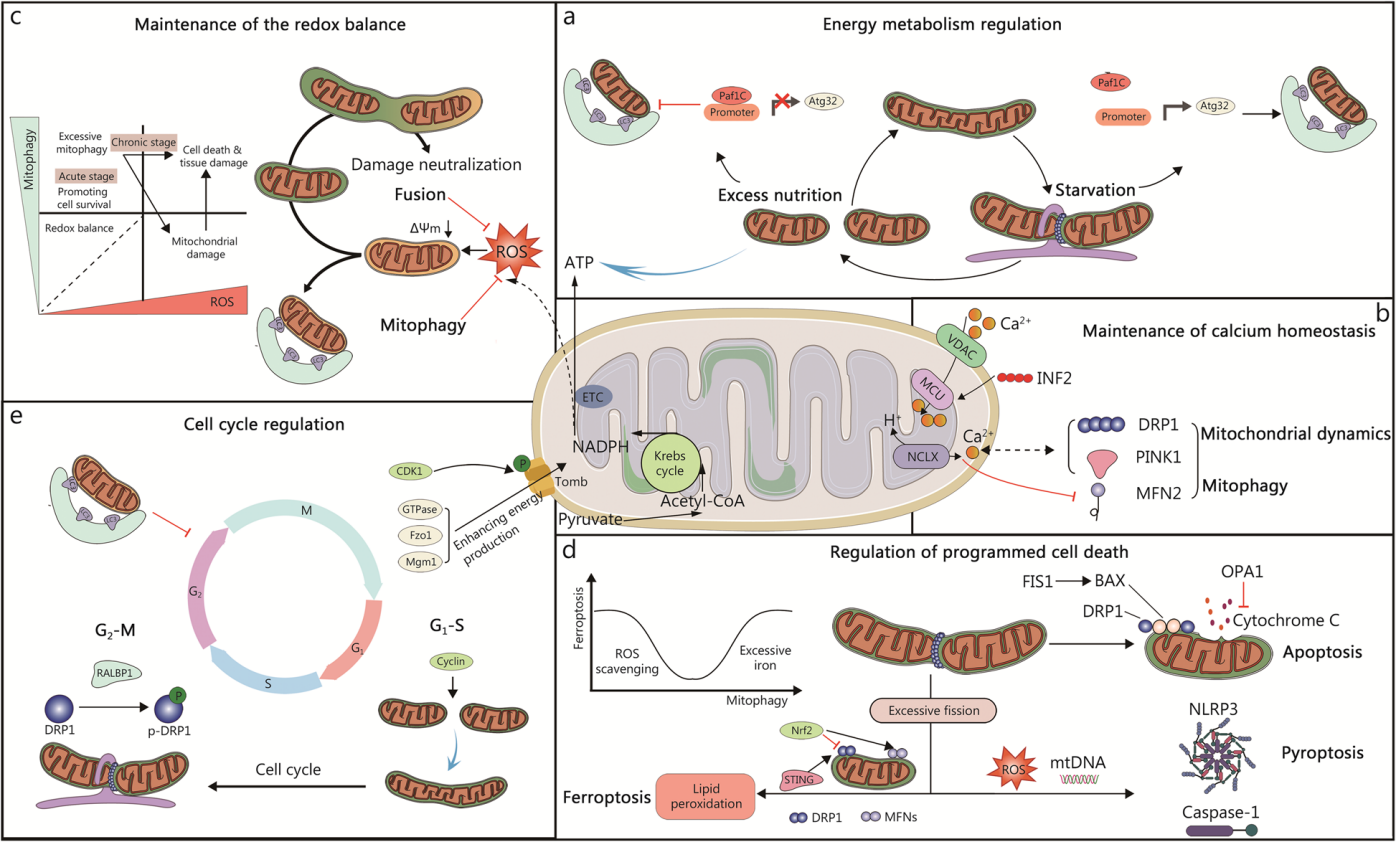
Physiological roles of mitochondrial quality control. **a** Mitochondria, as the core of energy metabolism, regulate their quality to adapt to the cellular environment under different bioenergy conditions. When nutrients are in excess, mitochondrial dynamics switch to division dominance and mitophagy is blocked. Under starvation conditions, the mitochondria fuse, and the mitochondrial autophagic flux is enhanced. Together, these regulations promote the balance of mitochondrial energy metabolism. **b** Mitochondria can act as calcium pools in cells, and there are extensive interactions between calcium ions and mitochondrial quality control to jointly regulate mitochondrial mass and calcium ion homeostasis. **c** Depolarizing mitochondria leads to oxidative stress. Mildly damaged mitochondria can fuse with healthy mitochondria to neutralize damage, and severely damaged mitochondria are cleared by mitophagy, but excessive mitophagy can also lead to oxidative stress. **d** Excessive mitochondrial fission is an early event in apoptosis, pyroptosis, and ferroptosis. Mitochondrial fragmentation promotes cytochrome and mtDNA release and causes oxidative stress. Furthermore, the regulation of ferroptosis correlates with mitophagy flux, and moderate mitophagy promotes the clearance of ROS, while excess mitophagy results in excess iron ions. **e** Mitochondria initiate fine kinetic processes to accommodate the mitochondrial genetic process in progeny during the cell cycle. Cyclin regulator CDK1 promotes protein import into the mitochondria to ensure mitochondrial energetic support during the cell cycle. Mitophagy reduces the pool of mitochondria in the cell, which would further limit the cell cycle. ATP adenosine triphosphate, BAX BCL2 associated X, CDK1 cyclin B/cyclin-dependent kinase 1, DRP1 dynamin-related protein 1, ETC electron transport chain, FIS1 fission protein 1, Fzo1 fuzzy onion 1, INF2 inverted formin 2, GTPase guanosine triphosphatase, MCU mitochondrial calcium uniporter, MFN mitofusin, Mgm1 mitochondria genome maintenance 1, mtDNA mitochondrial DNA, NCLX Na^+^/Ca^2+^/Li^+ ^exchanger, NLRP3 NLR family pyrin domain containing 3, Nrf2 nuclear factor E2-related factor 2, OPA1 optic atrophy 1, PINK1 PTEN-induced kinase 1, RALBP1 ralA binding protein 1, ROS reactive oxygen species, STING stimulator of interferon response cGAMP interactor, TCA tricarboxylic acid, VDAC voltage-dependent anion channel

Mitochondria play a vital role not only in regulating energy metabolism during physiological activities but also in regulating calcium homeostasis, redox homeostasis, PCD, and other essential processes. Numerous studies have highlighted their irreplaceable significance in these cellular functions. The subsequent section discusses the importance of MQC in ensuring and regulating these critical life activities (Fig. 2).

### Energy metabolism regulation

Mitochondria are the energy-generating component of cells, accounting for approximately 90% of cellular energy produced. MQC is involved in the regulation of energy metabolism, which is crucial to maintaining the energy supply of the body in the normal physiological state, most notably for organs and tissues with high energy demands such as the heart, liver, kidney, brain, and skeletal muscle. As the main energy donor, glucose is oxidized into pyruvate by glycolysis in the cytoplasm under normal physiological conditions, and then enters the mitochondria via the pyruvate transporter and is converted to acetyl-CoA, which further drives TCA cycle capacity. Theoretically, 1 mol of glucose produces 36 – 38 ATP molecules, of which 34 – 36 ATP come from OXPHOS and the rest from glycolysis [186].

Under the condition of excess nutrition or starvation, the MQC system is initiated to precisely regulate the dynamic balance of mitochondria at the subcellular level to ensure the balance of energy demand and supply. The mitochondria tend to maintain their isolated state in the presence of a nutrient-rich environment, while they undergo elongation under conditions of cellular starvation [187, 188]. Previous studies have shown that mitochondrial fragmentation can be detected when the β cell line INS1 is simply exposed to high fat or combined with high glucose after 4 and 24 h [189]. Furthermore, reduced mitochondrial size in muscle and decreased MFN2 expression was observed in mouse models of type 2 diabetes mellitus (T2DM) and obesity [190–192]. In contrast to overnutrition, the recruitment of DRP1 to mitochondria is inhibited during starvation, resulting in suppressed mitochondrial fission and elongation, along with increased amounts of mitochondrial cristae and ATP synthesis capacity to maintain the required ATP demand when nutrient supply is limited [188, 193]. These studies suggest that starvation-induced mitochondrial elongation may be a positive mechanism for mitochondrial bioenergetic efficiency.

An increasing number of studies also suggest that mitophagy is involved in regulating energy metabolism [194–196]. Under glucose-rich conditions, the polymerase-associated factor 1 complex (Paf1C) can maintain low levels of mitophagy by binding to the promoter of the *Atg32* gene, while upon starvation, Paf1C dissociates from *Atg32*, resulting in increased expression of the *Atg32* gene and induction of mitophagy [197]. Deletion of *Fundc1* induces defective mitophagy and impaired MQC, leading to more severe obesity and insulin resistance (IR) in mice fed a high-fat diet (HFD) [198]. Furthermore, mitochondria undergo different topological changes during starvation, either becoming swollen or developing a donut shape, and are further removed by mitophagy, while mitophagy-resistant mitochondria reintegrate via mitochondrial fission and fusion [199].

As a supplementary pathway to mitophagy, MDVs envelop and release damaged mitochondrial material in brown adipose tissue, leading to the obstruction of PPARγ signaling and ultimately inhibiting the TCA cycle in brown adipose tissue [200]. This phenomenon can be alleviated by the engulfment of extracellular vehicles (EVs) generated by these MDVs.

In conclusion, the MQC system can accurately regulate energy metabolism at the subcellular level, maintain the dynamic balance of mitochondria, and ensure the energy demand of the body.

### Maintenance of calcium homeostasis

As a second cellular messenger, calcium is involved in regulating gene expression, proliferation, differentiation, metabolism, cell death, and survival, and plays an irreplaceable role in cellular pathophysiology [201]. Mitochondria buffer intracellular calcium levels by absorbing, storing, and releasing Ca^2+^, while MQC as an important mechanism for regulating cellular fate, also plays an important role in maintaining calcium homeostasis. The OMM regulates the voltage-dependent anion channel (VDAC) to facilitate the absorption of calcium ions from the cytoplasm into cells. Subsequently, these calcium ions traverse through the mitochondrial calcium uniporter (MCU) complex located in the IMM, thereby participating in the regulation of energy production and metabolism [186, 202, 203]. However, the calcium ions of the mitochondrial matrix mainly flow out to the cytoplasm through the Na^+^/Ca^2+^ exchanger (NCLX) and H^+^/Ca^2+^ antiporter [204]. In mitochondria, Ca^2+^ can trigger apoptosis by opening the mitochondrial permeability transition pore (mPTP) [205, 206]. The decrease of the adenine nucleotide pool decreases and the increase of phosphate, matrix calcium, and mitochondrial ROS (mtROS) will promote the massive outflow of particles less than 1.5 kD, such as protons and calcium ions, from the mitochondrial matrix [207, 208]. In addition, both mitochondria and ER are the main storage areas of intracellular calcium ions. Mitochondria interact with the ER through the mitochondrial ER contact site (MERCS) to regulate cellular and mitochondrial calcium homeostasis [209–212].

In the process of responding to changes in energy metabolism and cellular activity, there exists a mutual influence and regulation between MQC and calcium homeostasis. Early findings demonstrated that extracellular or intracellular calcium can inhibit mitochondrial disruption by inhibiting intracellular C2-ceramide [213]. Subsequently, it was confirmed that intracellular calcium regulates mitochondrial fission by controlling DRP1 localization, where calcineurin mediates DRP1 dephosphorylation, which then induces DRP1 transfer to mitochondria and accumulates on the OMM, thereby increasing the rate of mitochondrial fission [214, 215]. Moreover, in MERCS, INF2 was found to mediate actin polymerization, and then control MCU-mediated calcium uptake and DRP1-dependent mitochondrial assembly, thus promoting mitochondrial fission [216, 217]. Mitochondrial division inhibitor 1 (mdivi-1) effectively inhibits DRP1-mediated mitochondrial fission and also induces mitochondrial depolarization, ER Ca^2+^ depletion, and Ca^2+^ signaling regulation [218]. Ablation of constitutive DRP1 leads to enlarged mitochondrial morphology, increased mitochondrial calcium uptake, and abnormal mitochondrial function [219]. Moreover, Ca^2+^ is also related to the regulation of mitophagy. Calcium ion sensors RHOT1 and RHOT2 mediate Parkintrans location, which induces mitophagy after binding to mitochondria [220, 221]. Calcium flows from the ER to MERCS and into the mitochondria, uncoupling the mitochondria from the ER by disrupting MFN2, then regulating PINK1/Parkin-mediated phosphorylation-ubiquitination and inducing mitophagy [124, 222]. MUL1 deficiency increases MFN2 activity and decreases ER-mitochondria coupling, which further leads to increased cytoplasmic Ca^2+^ load, activating of calcineurin, and induction of DRP1-dependent mitochondrial fragmentation and mitophagy [223]. The mutual regulation between mitophagy and Ca^2+^ uptake and release is significant. The latest research findings indicate that transmembrane BAX inhibitor motif containing 6 (TMBIM6), an ER protein with calcium leakage activity regulates mitochondrial Ca^2+^ uptake by preventing the oligomerization of VDAC1, thereby causing impaired mitophagy and disrupted mitochondrial biosynthesis, further leading to compromised mitochondrial respiration and ATP production [224]. At the same time, damaged mitochondria induce the intracellular migration of calcium via the MCU, while mitophagy significantly inhibits this trend after clearing damaged mitochondria [225].

In summary, calcium imbalance leads to MQC dysregulation, resulting in mitochondrial dysfunction. Simultaneously, alterations in MQC can significantly influence the content and distribution of calcium ions within the cell. Further research on VDAC, MCU, and MERCS is expected to elucidate the connection between MQC and calcium homeostasis regulation.

### Maintenance of the redox balance

mtROS is the main component of intracellular ROS, and is an important signaling molecule involved in the regulation of physiological functions, playing a significant role in maintaining the redox balance [186]. The production of mtROS is primarily influenced by OXPHOS capacity, the oxidation of NADPH/NADH, and the biosynthesis of heme and iron-sulfur centers [226–228]. During OXPHOS, electrons leak from mitochondrial complexes 1 and 3 and interact with oxygen molecules to produce the most dangerous mtROS: superoxide anion [229, 230]. Moreover, mitochondrial complex 1 can also deliver electrons from NADPH/NADH produced in the TCA cycle to oxygen molecules, promote the oxidation of NADPH/NADH and the generation of superoxide radicals, and then induce a large amount of mtROS production [231, 232]. Excessive production of mtROS can induce oxidative stress of lipids and protein and DNA damage. To avoid further cell damage, mitochondria eliminate excess ROS, thus maintaining the redox homeostasis via a system of antioxidants including superoxide oxidase, catalase, glutathione peroxidase, and antioxidant factors such as glutathione, vitamins [233, 234]. An increasing number of studies suggest that MQC plays a crucial role in the redox imbalance mediated by mtROS.

Mitophagy limits mtROS overproduction and maintains the redox balance by sequestering and engulfing aging and damaged mitochondria [235, 236]. Studies have shown that long-term exposure to PM2.5 can induce the massive production of mtROS and impair mitophagy, leading to redox imbalance. However, the addition of mitochondria-targeted antioxidant Coenzyme Q can improve mitophagy and oxidative damage by reducing the accumulation of ROS [237, 238]. Moreover, mitophagy inhibitors have been shown to induce significant ROS accumulation and redox imbalance, and eventually aggravate the disease state [239–241]. Among these inhibitors, endogenous and exogenous substances such as α-ketoglutarate and rapamycin, as well as signaling pathways such as AMPK, MAPK, and Nrf2, can maintain the redox balance of mitochondria by increasing mitophagy to clear ROS and oxidative stress [242–246]. However, increased mitophagy is often an early response to promote cell survival, and a chronic state of excessive mitochondrial damage can induce pathological increased mitophagy, resulting in cell death and tissue damage [246]. Thus, excessive mitophagy may also lead to an increase in mtROS, which could further induce redox imbalance. Additionally, under hypoxic conditions, mitochondria undergo widespread ubiquitination facilitated by UPS to mitigate mtROS accumulation and oxidative stress levels. The ubiquitination process activates receptor-dependent mitophagy, specifically emphasizing BNIP3/Nix, rather than the classical pathway mediated by Parkin [123]. Recent research has reported a close association between the biogenesis of peroxisomes and MPVs. The MPVs-encapsulated Pex3 and Pex14 are crucial components of peroxisomal precursors, while the lipids and peroxides carried by MPVs serve as metabolic substrates and tools of the peroxisomes [247]. MPVs primarily regulate the cellular redox balance through the encapsulation and release of mitochondrial contents [165].

Mitochondrial fission and fusion also play an important role in regulating oxidative stress. Under the pressure of oxidative stress, mitochondrial fusion increases to reduce ROS production [248, 249]. Increased MFN2 expression promotes mitochondrial fusion and autophagy, and reduces ROS, thereby maintaining the redox balance [250, 251]. In vitro experiments confirmed that increasing the ratio of GSSG/GSH leads to cis-oligomerization of MFN disulfide bonds and promotes mitochondrial fusion [252, 253]. A study has confirmed that C684 residue is required for MFN2 disulfide bond and fusion activity. When C684 is absent, MFN2 is more prone to redox changes, which affects the energy export of mitochondria [254]. In the newly proposed topological modification of MFN, it was also indicated that residue C684 can directly sense the redox environment of mitochondria [255]. In addition, studies have confirmed that MFN2 is a prerequisite for mitochondrial respiration to stress and ROS production, and the knockout of MFN2 in macrophages leads to ROS production defects and impairs immune response function [256, 257]. Furthermore, when oxidative stress escalates, the activation of the Nrf2 pathway not only aids in upregulating antioxidant defenses and restoring redox balance but also promotes the proteasomal degradation of the mitochondrial fission protein DRP1 [258]. It was also confirmed that the degradation of DRP1 is contingent upon non-ubiquitinated degradation facilitated by the 20S proteasome, while MFN degradation relies on ubiquitin-mediated degradation of the 26S proteasome. The oxidative stress can promote the breakdown of 26S proteasome into 20S and 19S subunits, which in turn promotes the disassembly of DRP1 and stabilizes MFN, thus reducing mitochondrial fission [259]. Overall, under the stimulation of oxidative stress, cells can protectively reduce the accumulation of ROS and restore the redox balance by regulating the MQC system.

### Cell cycle regulation

The cell cycle comprises a series of ordered lifecycle stages during which a cell undergoes division from one mother cell into two daughter cells, facilitating cellular renewal, growth, and replication of genetic material [260]. The cell cycle can be divided into two main stages: the mitotic phase (M phase) and interphase. The interphase, consisting of the G_1_ phase, S phase, and G_2_ phase, serves as the preparatory stage of the cell cycle that provides conditions for subsequent division, while the M phase is the primary stage of cell division. To meet the material and energy requirements of various stages in the cell cycle, the functionality and morphology of mitochondria undergo dynamic changes [261]. During the G_1_ and G_2_ phases, mitochondria form an interconnected network, which subsequently undergoes division in mitosis and the S phase [262]. In addition to exerting influence on the levels of materials and energy required for cellular activities, mitochondria can also passively participate in regulating cell cycle progression by affecting specific checkpoints.

The translocase at the outer membrane of mitochondria (TOM complex) is a protein translocase essential for the import of raw materials into mitochondria during mitochondrial biogenesis [164]. In the M phase, the expression of TOM6, a key component of the TOM complex, significantly increases. Simultaneously, the cell cycle regulatory factor CDK1 facilitates the phosphorylation of TOM6 Ser16 [263]. The phosphorylation of TOM6 enhances the steady-state level and activity of the TOM complex, thereby promoting protein entry into mitochondria. This process involves an increase in mitochondrial M-phase-specific regulatory GTPases, Fzo1 and Mgm1, which ultimately enhance energy production to meet the heightened energy demands during the M phase [55]. Similarly, during the early stages of the M phase, RALBP1, the effector protein of RALA, interacts with CDK1/Cyclin B to phosphorylate DRP1 at Ser616, thus promoting mitochondrial fission [264]. As part of a feedback regulatory mechanism, acute loss of DRP1 enhances the mitochondrial recruitment of Cyclin E, a crucial protein regulating the G_1_/S transition of the cell cycle, which prevents its degradation and promotes the cellular transition from G1 to S phase [265]. However, chronic inhibition of DRP1 leads to mitochondrial hyperfusion, which in turn results in a p53-dependent blockade of S-phase entry [265]. Subsequently, DRP1 deficiency-induced mitochondrial hyperfusion also leads to DNA replication stress-induced ATM-dependent G_2_/M arrest through the excessive accumulation of Cyclin E [266]. Depletion of OPA1 can ameliorate the aforementioned phenomena [266]. It highlights the necessity of mitochondrial fission during the S, G_2_, and M phases following the completion of the G_1_/S transition to ensure that daughter cells inherit consistently healthy mitochondria. In cells containing damaged mitochondria, PINK1/Parkin-mediated mitophagy is activated, promoting optineurin (TBK1) mitochondrial translocation and phosphorylation [267]. Consequently, this leads to G_2_/M cell cycle arrest and inhibition of mitosis [267]. In summary, MQC actively participates in regulating the cell cycle to ensure the proper transmission of mitochondrial genetic material and the stability of intracellular energy supply during cell growth.

### Regulation of PCD

Different types of cell death are the inevitable fate of all life to maintain healthy cell renewal and the homeostasis of the organism [268, 269]. PCD is the primary means of cell renewal and is controlled by numerous genes. Strict MQC is essential for mitochondrial function and is closely associated with physiological PCD.

Apoptosis is a type of PCD executed by caspases and is characterized by specific morphological changes, including cell membrane blebbing, nucleus condensation, condensation and fragmentation of genetic materials, and formation of apoptotic bodies [268, 270]. Two distinct apoptosis pathways include intrinsic and extrinsic pathways, known as the mitochondrial and death receptor pathways, respectively [271]. Intrinsic apoptosis is caused by DNA damage, hypoxia, metabolic stress, and other stimulations, inducing the expression of pro-apoptotic BH3-only proteins (such as BIM, PUMA, BAD), binding and neutralizing pro-survival BCL2 proteins (such as BCL2, BCL-XL, and BFL-1), thus releasing apoptosis effectors BAK and BAX. It leads to mitochondrial outer membrane permeabilization and subsequent release of mitochondrial proteins, including cytochrome C, the second mitochondrial activator of caspases [272, 273]. Mitochondrial fission is considered an early event during apoptosis [274]. During apoptosis, DRP1 redistributes to mitochondrial membranes and induces mitochondria fragmentation [275]. DRP1 is irreversibly locked on the membrane in a BAX/BAK-dependent manner after mitochondrial fragmentation, accompanied by stable SUMOylation [276]. Deficiency of DRP1 delays the release of cytochrome C and apoptosis, which is related to the enclosure of cytochrome C into the highly stacked cristae [275, 277]. Loss of FIS1 has been found to reduce mitochondrial fission and apoptosis, partly because it inhibits BAX translocation [278]. Correspondingly, insufficient mitochondrial fusion may also lead to mitochondrial fragmentation during apoptosis. OPA1 participates in controlling cristae remodeling and cytochrome C release, whereas deletion of OPA1 leads to the disruption of the mitochondrial network and apoptosis [76, 279]. Interestingly, BAX and BAK physiologically regulate MFN2 activity and normal morphogenesis of mitochondria; yet, during apoptosis, BAK dissociates from MFN2 and interacts with MFN1, which may reduce mitochondrial fusion and induce mitochondrial fragmentation [280, 281]. In addition, MFN1 phosphorylation by ERK also influences BAK oligomerization under apoptotic stimuli [70]. Dynamic PGAM5 multimers have been reported to act as a molecular switch to coordinate mitophagy and apoptosis. In response to distinct stresses, PGAM5 dephosphorylates BCL-XL to inhibit apoptosis or FUNDC1 to enhance mitophagy by switching between dimeric and multimeric states [282]. Surprisingly, in the absence of mitophagy, PINK1/Parkin can promote cell apoptosis through a non-BAX pathway [283]. The ubiquitination effect of UPS on OMM promotes the release of cytochrome C, directly activates autophagy receptors, upregulates mitophagy, and inhibits apoptosis [283].

Pyroptosis is an immunogenic PCD driven by inflammasome [273]. Pathogen- or danger-associated molecular patterns trigger canonical caspase-1 inflammasome pathway. In this process, activated caspase-1 directly cleaves GSDMD and releases N-terminal fragments to bind to phosphatidylinositol on the plasma membrane, forming membrane pores [268, 284]. Meanwhile, caspase-1 cleaves pro-IL-1β and pro-IL-18 to produce mature IL-1β and IL-18, which are released from the GSDMD pores. It has been reported that DRP1-mediated excessive mitochondrial fission contributes to the activation of caspase-1 and NLRP3 inflammasome, which may be related to the increased ROS induced by mitochondrial dynamics dysfunction [285, 286]. Analogously, ROS also acts as a bridge between defective mitophagy and pyroptosis [287, 288]. Defective mitophagy can result in mitochondrial dysfunction, increased mPTP, and mtDNA leakage, leading to a vicious cycle between MQC deficiency and excessive inflammation [289].

Ferroptosis is an iron-dependent form of PCD, primarily driven by the toxic accumulation of cell membrane lipoperoxides [290]. Mitochondria, serving as the primary sources of intracellular ROS and ATP, are also actively involved in various processes, including the TCA cycle, lipid biosynthesis, and glutaminolysis [291]. These biological components play a key regulatory role in the regulation of the ferroptosis process. Within cellular activities, the interplay between MQC and ferroptosis is relatively complex and can be dissected at several key junctures. As described above, Nrf2 regulates proteasome genes that contribute to DRP1 degradation, upregulates MFN2 protein levels to promote mitochondrial fusion and inhibit fission, and functions as a promoter protein for mitochondrial biogenesis, the absence of Nrf2 greatly upregulates ferroptosis [258]. Additionally, STING accumulates significantly during its transportation from the ER to mitochondria, where it binds to MFN1/2, thereby triggering mitochondrial fusion [292]. During this process, the increase in ROS and fatty acid levels leads to lipid peroxidation, consequently promoting ferroptosis. In contrast, mitophagy exhibits a dual role in the regulation of ferroptosis. The release of ROS and certain pro-apoptotic factors from damaged or dysfunctional mitochondria promotes ferroptosis. This positive regulation is notably inhibited when mitophagy clears abnormal mitochondria [293]. Furthermore, during mild stress or in the early stages of iron overload, mitophagy can reduce the source material of ROS in ferroptosis by chelating iron. However, further expansion of mitophagy may ultimately provide additional iron, thereby amplifying lipid peroxidation and ferroptosis [294]. Current perspectives suggest that the direction of mitophagy regulation in ferroptosis primarily depends on the magnitude of mitophagy flux. In conclusion, MQC plays a pivotal role in regulating ferroptosis, and in-depth exploration of their interconnectedness and mechanisms will offer new insights into understanding cellular life processes and disease progression.

## MQC in human diseases

### MQC and cancer

#### Tumor initiation and progression

Cancer cells can redirect metabolites towards biosynthetic pathways to support their rapid proliferation and accumulate the cellular building blocks required for tumor growth. Enhanced mitochondrial biogenesis contributes to enhanced OXPHOS and fosters cancer growth [295]. Migratory/invasive cancer cells utilize PGC-1α to boost mitochondrial biogenesis, OXPHOS, and oxygen consumption rates, promoting cancer cell invasion and metastasis [296]. Mitochondrial biogenesis mediated by mitochondrial Ca^2+^ signaling promotes the growth of colorectal cancer cells in vitro and in vivo [297]. Inhibiting mitochondrial biogenesis through genetic or pharmacological means can suppress tumor cell metabolism and activity, and inhibit tumor progression [298, 299]. However, it is interesting to note that low levels of mitochondrial biogenesis have been linked to sorafenib resistance in hepatocellular carcinoma. The study found that mitochondria in sorafenib-resistant cells maintain greater functional and morphological integrity under sorafenib treatment, but their number was lower. This can be attributed to the decrease in mitochondrial biogenesis, which is caused by the accelerated degradation of PGC-1β [300]. Cancer stem cells (CSCs) are distinct subpopulations of cancer cells with stem cell-like abilities that are more resistant to chemotherapy, leading to tumor recurrence. By enhancing mitochondrial biogenesis, CSCs maintain mitochondrial content and metabolic homeostasis to support cancer growth. Targeting mitochondrial biogenesis may be a potential therapeutic option to eliminate CSCs [301].

Imbalanced mitochondrial dynamics have a significant impact on cancer development and metastasis. Typically, mitochondria are fragmented and distributed within different types of tumor cells through fission, such as lung cancer [302], colon cancer [303], breast cancer [304], neuroblastoma [305], melanoma [306], ovarian cancer [307], prostate cancer [308], pancreatic cancer [309]. Carcinogenic mutations are a major cause of excessive mitochondrial fission, with BRAF^V600E^ or RAS^G12V^ mutations driving MAPK pathway activation to induce mitochondrial fission and promote tumor growth. Inhibiting DRP1 or promoting fusion can induce tumor cell death [306, 310–312]. The tendency of tumor cells to undergo mitochondrial fission is related to metabolic reprogramming, cell cycle progression, and increased migration, invasion, and metastatic potential [6, 313]. During mitosis in tumor cells, mitochondrial fission is crucial to ensure equal segregation of mitochondrial contents between daughter cells, which is important for the rapid proliferation of tumor cells. Lack of mitochondrial fission induces cell death and cellular dysfunction due to replication pressure and compromised genomic integrity [312, 314]. Additionally, excessive mitochondrial fission resulting in mitochondrial fragmentation redirects metabolism to the peripheral cytoskeleton’s lamellipodia, which serves as a concentrated energy source for tumor cell movement and invasion, promoting tumor cell invasion and metastasis [315]. Therefore, targeting mitochondrial fission or promoting fusion is a potential therapeutic strategy for many cancer treatments [6]. Yu et al. [316] proposed that the main tumor suppression mechanism of promoting mitochondrial fusion is to enhance mitophagy, which proportionally reduces mitochondrial mass and ATP production. However, mitochondrial dynamics exhibit heterogeneity in cancer. Overactive mitochondrial fusion has been observed in tumor tissues of hepatocellular carcinoma patients and tumor-like organoids of cholangiocarcinoma in vitro. Knockdown of OPA1 or MFN1 to inhibit mitochondrial fusion weakened oxygen consumption and cellular ATP production of tumor cells, suppressed cell growth in vitro, and inhibited tumor formation in vivo. This inhibitory effect was related to inducing cell apoptosis but not to cell cycle arrest [317]. Similarly, researchers found that enhancing mitochondrial fission inhibited signal transduction and metastasis of triple-negative breast cancer while enhancing mitochondrial fusion overcame the inhibitory effect of fission on migration, signal transduction, and metastasis. Further exploration of existing datasets on breast cancer revealed that increased expression of mitochondrial fission-related genes was associated with improved survival in human breast cancer [318]. Therefore, more mechanistic studies are needed to comprehensively understand how mitochondrial dynamics imbalance leads to cancer occurrence and progression. Additionally, OPA1-mediated mitochondrial fusion in endothelial cells promotes tumor angiogenesis, affecting tumor growth and metastasis [319]. In addition, activation of the CDK1/Cyclin B-DRP1 pathway induces mitochondrial fission, facilitating cell cycle progression and increasing the sensitivity of tumors to radiotherapy [320]. In CSCs, mitochondrial fission is often associated with increased rates of mitophagy, epithelial-to-mesenchymal transition, and increased rates of glycolysis. Conversely, mitochondrial fusion is typically associated with increased oxidative phosphorylation and resistance to therapy [321].

Mitophagy suppresses tumors by eliminating dysfunctional mitochondria that disrupt cellular metabolism and promote cellular transformation and tumorigenesis. Conversely, mitophagy-mediated clearance of apoptotic mitochondria in cancer cells may have a cytoprotective effect [322, 323]. Mitophagy is closely related to the metabolic reprogramming of tumor cells. Cancer cells generate energy through aerobic glycolysis even under normal oxygen conditions, and the unique metabolic profile of reduced oxidative phosphorylation and enhanced aerobic glycolysis in cancer cells is known as the Warburg effect [324]. Defective mitophagy leads to the Warburg effect, mitochondrial metabolic changes, and associated enhancement of glycolysis. Mitophagy deficiency increases the expression of exokinase 2, resulting in enhanced glycolysis in liver cancer and promoting tumor growth [325]. P53/BNIP3-dependent mitophagy suppresses tumor growth by inhibiting glycolysis in radioresistant cancer cells of head and neck squamous cell carcinoma [326]. High expression of CD44 is linked to enhanced malignant potential of esophageal squamous cell carcinoma, and Parkin-mediated mitophagy promotes CD44^+^ activity, while inhibition of mitophagy leads to oxidative stress and cell death [327]. NIX-mediated mitophagy promotes the clearance of ROS induced by hypoxia in highly invasive glioblastoma, maintains cancer stem cells, and promotes tumor cell survival [328]. In CSCs, mitophagy is utilized as a positive regulatory mechanism to alter their metabolic state for better adaptation to various metabolic stresses [301]. Mitophagy maintains the stemness of liver cancer CSCs by removing the tumor suppressor p53 [329]. Additionally, Towers et al. [157] have revealed that autophagy-dependent cancer cells can utilize MDVs to compensate for the removal of damaged mitochondria and preserve mitochondrial homeostasis in the presence of mitophagy inactivation.

#### Tumor immunity

Immune cells participate in the regulation of tumor biology, wherein appropriate mitochondrial function is of crucial importance for the phenotype, proliferation, and differentiation of immune cells [330]. However, driven by tumor progression, nutrient depletion, hypoxia and production of immunosuppressive metabolite in the tumor microenvironment (TME) can alter anti-tumor activities of immune cells, ultimately contributing to immune escape [331]. Therefore, understanding the MQC changes of immune cells in the TME is a promising avenue in cancer research.

As the principal effectors of anti-tumor immunity, functional cytotoxic CD8^+^ T cells are closely related to the survival of cancer patients [332, 333]. Naïve CD8^+^ T cells maintain their low metabolic demands by relying on OXPHOS; while the T cell receptor (TCR) is activated, metabolic reprogramming occurs in effector T cells, including aerobic glycolysis, OXPHOS, and glutaminolysis to meet the requirements of proliferative outbreak and effector functions [332, 334]. Both metabolic stresses of T cells driven by TME fuel depletion and increased expression of inhibitory receptors like programmed cell death-1 (PD-1), cytotoxic T lymphocyte-associated protein 4 are the essential factors that result in tumor-infiltrating CD8^+^ T cells exhaustion [332, 335]. Experiments have demonstrated that the TME can impair PGC-1α-mediated mitochondrial biogenesis and function in tumor-infiltrating CD8^+^ T cells, in part by chronic activation of Akt, and these processes can be reversed by PGC-1α overexpression [336]. In addition, nuclear receptor coactivator 2 has also been proven to be an upstream factor in the promotion of PGC-1α expression, which is involved in the regulation of CD8^+^ T cell-mediated anti-tumor immune responses [333]. Malinee et al. [337] reported that EnPGC-1, an epigenetic activator of PGC-1α/β, enhances mitochondrial biogenesis and increases OXPHOS and FAO in effector T cells, improving the synergistic effect of PD-1 blockade therapy. Notably, enforced mitochondrial biogenesis can promote differentiation of CD8^+^ T cells into memory T cells with better recall capacity [338]. Mitochondrial dynamics also play a crucial role in controlling T cell phenotype. During T cell activation, DRP1-mediated mitochondrial fragmentation can be observed and migrate to the immune synapses, inducing metabolic gene transcription through modulating the balance between AMPK and mTOR/cMyc under an appropriate calcium current [88, 339, 340]. Here, loosely attached mitochondrial cristae in effector T cells lead to lower ETC efficiency and promote aerobic glycolysis [339, 341]. Correspondingly, OPA1-mediated mitochondrial fusion or DRP1 deletion seems to induce the generation of memory T cells. As a result, abnormal mitochondrial dynamics may result in T-cell dysfunction and impaired anti-tumor response. It has been reported that PD‐1 signaling prevents DRP1-mediated mitochondrial fission via regulating mTOR and ERK pathways to impair CD8^+^ T cell function in TME [342]. Mitophagy in CD8^+^ T cells is also influenced by the TME. The combined challenge of TCR stimulation, microenvironmental stressors, and PD-1 signals inhibit mitophagy in tumor-infiltrating CD8^+^ T cells, which leads to the accumulation of depolarized mitochondria and ultimately T cell exhaustion [343]. Persistent activation of ER stress in TME causes T cell dysfunction, whereas interestingly, transient activation of ER stress by carbon monoxide promotes T cell anti-tumor function through driving mitochondrial biogenesis and protective mitophagy [344]. Moreover, Denk et al. [345] recently revealed that the pharmacological inducer of Pink1-dependent mitophagy in CD8^+^ T cells promotes T memory stem cell formation and anti-tumor effects in colorectal cancer, showing a beneficial effect in chimeric antigen receptor T-cell therapy.

Natural killer (NK) cells are the major effectors of innate immunity against cancer [346]. Increasing evidence suggests that changes in mitochondrial dynamics are connected to the anti-tumor capacity of NK cells. In comparison to the normal NK cells, small and fragmented mitochondria are observed in the tumor-infiltrating NK cells from patients with liver cancer, accompanied by lower mitochondrial mass [347]. To be more specific, the hypoxic state of TME causes mitochondrial fragmentation by increasing mTOR-DRP1 signaling in NK cells, resulting in abnormal mitochondrial respiration and tumor evasion of immune detection, which can be reversed by DRP1 inhibitor [347].

Tumor-associated macrophages (TAMs) are the most abundant components in the TME. After being recruited to TME by tumor-related cytokines, TAMs promote cancer angiogenesis, metastasis, and immunosuppression through the further secretion of cytokines, chemokines, growth factors, and matrix metalloproteinases [348]. In other words, the extent of TAM infiltration is closely associated with a poor prognosis of cancer. In tumor cells, mitochondrial fission mediated by DRP1 enhances the recruitment of TAMs by releasing mtDNA, which promotes the release of the chemokine CCL2, thus facilitating tumor progression [349]. ROS plays a crucial role in the inflammatory state of the TME and immune escape mediated by PD-L1 expression. However, TAMs that T cell immunoglobulin and mucin domain containing-4-positive exhibit elevated levels of oxidative phosphorylation and upregulate mitophagy to eliminate ROS and other hazardous factors, thus promoting tumor metastasis [350]. Furthermore, the activation of the IL-33/ST2 signaling pathway significantly upregulates mitophagy processes, thereby modulating the oxidative phosphorylation and polarization levels of M2 macrophages in the TME to inhibit tumor growth [351].

**Fig. 3:**
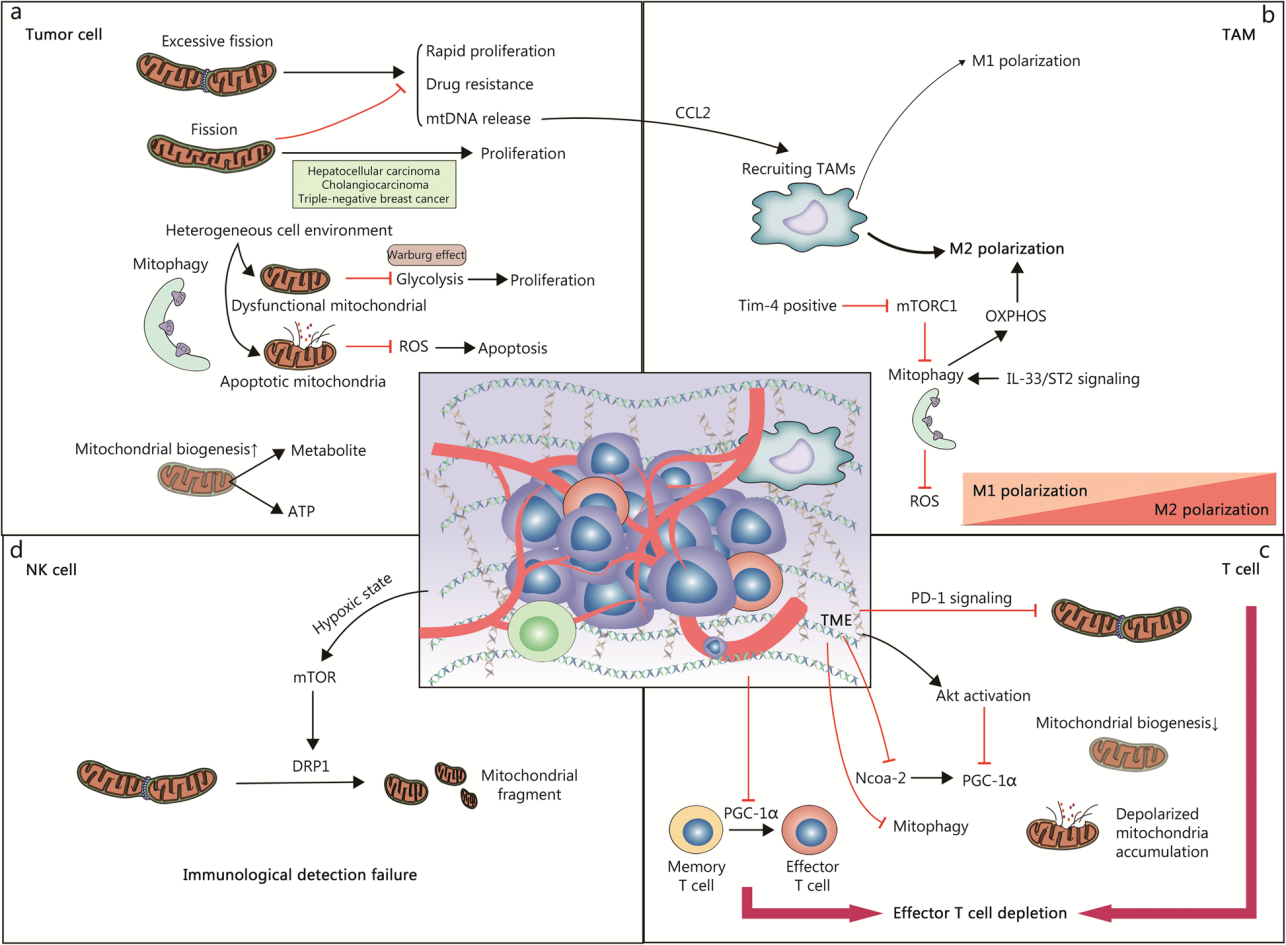
Mitochondrial quality control and cancer. **a** The role of mitochondrial quality control varies in different tumors. Mitophagy could remove dysfunctional mitochondria and hinder the proliferation of tumor cells and, on the other hand, the clearance of apoptotic mitochondria can remove the oxidative stress adaptation of tumor cells and subsequently promote tumor cell apoptosis. Tumor cells tend to have highly active mitochondrial biogenesis, and the high density of mitochondria provides both energy and metabolic intermediates for tumor proliferation. **b** In tumor-associated macrophages, mitophagy promotes oxidative phosphorylation and the clearance of ROS, thereby promoting the M2 phenotype of tumor macrophages. **c** In T cells, the insufficiency of mitochondrial fission, mitochondrial biogenesis, and mitophagy leads to the depletion of effector T cells. **d** In NK cells, the hypoxic microenvironment promotes mitochondrial fragmentation, which leads to the failure of immune surveillance. ATP adenosine triphosphate, DRP1 dynamin-related protein 1, CCL2 C–C motif chemokine ligand 2, TAM tumor-associated macrophage, mTOC1 mammalian target of rapamycin complex 1, Akt v-akt murine thymoma viral oncogene homolog, PD-1 programmed cell death protein 1, NK natural killer, IL interleukin, mtDNA mitochondrial DNA, OXPHOS oxidative phosphorylation, PGC-1α PPAR-γ coactivator-1α, ROS reactive oxygen species, TME tumor microenvironment

Overall, the mitochondrial function of immune cells, particularly the MQC system, is affected by complex TME, which may result in decreased tumor susceptibility and immune escape. Therapeutic strategies aimed at maintaining MQC stability in TME cannot be overlooked for their role in inhibiting tumor development (Fig. 3).

### MQC and cardiovascular disease

**Fig. 4:**
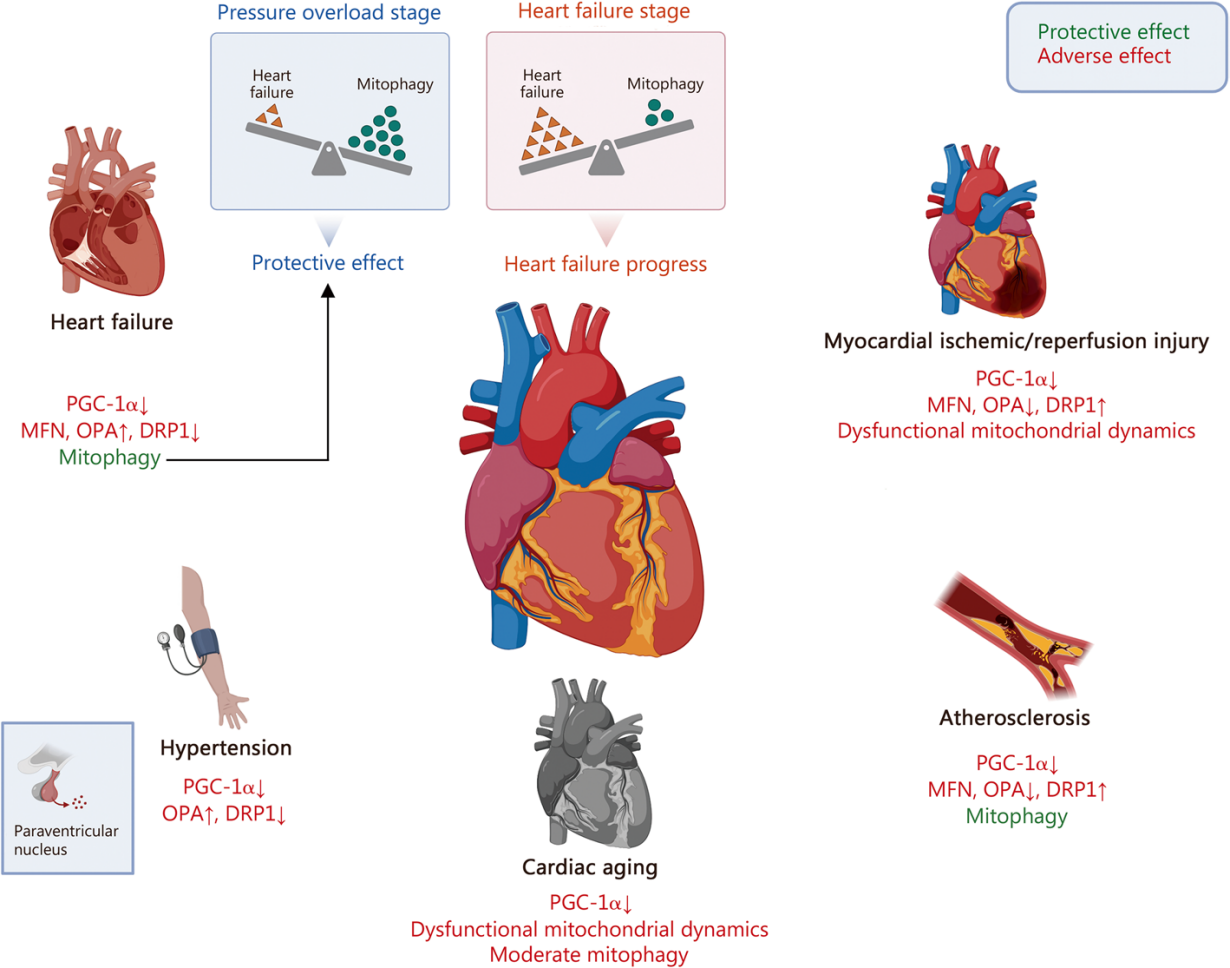
Mitochondrial quality control and cardiovascular disease. The common features of mitochondrial quality control in cardiovascular disease are the downregulation of mitochondrial biogenesis, a shift of mitochondrial dynamics to fission phenotypes, and the downregulation of mitophagy. In the early stage of most cardiovascular diseases, mitophagy is properly upregulated to compensate for mitochondrial quality disorders, but in the stage of disease progression, mitophagy is downregulated. Created by Biorender.com, accessed on 25 Aug 2023. DRP1 dynamin-related protein 1, MFN mitofusin, OPA optic atrophy, PGC-1α PPAR-γ coactivator-1α

The heart has a high energy demand, and mitochondria maintain the physiological function of cardiomyocytes by supplying large amounts of energy constantly and rapidly [352]. Thus, mitochondria account for 30% of cardiomyocyte volume [353]. Mitochondrial dysfunction is always accompanied by impaired MQC and is significantly linked to the development of various cardiovascular diseases (Fig. 4).

#### Heart failure (HF)

HF is a complex clinical syndrome, which is characterized by insufficient cardiac output due to abnormalities of cardiac structure/function [354]. HF is typically the terminal state of various cardiovascular diseases. Recent evidence has demonstrated that MQC plays a crucial role in the process of HF.

In a clinical trial of HF, microarray analysis of myocardial tissues from cardiomyopathy HF patients and healthy controls demonstrated that the expression level of PGC-1α, the major regulator of mitochondrial biogenesis, was significantly reduced in the myocardium of HF patients [355]. Similarly, the protein expression of PGC-1α was found to be downregulated in both patients with HF and a rat model of HF, relating to the left ventricular ejection fraction (LVEF) [356]. Heart-specific PGC-1α deletion in mice induced failing heart phenotypes, manifested by reduced LVEF, enlarged left ventricular, and increased natriuretic peptide type B, accompanied by the suppression of energy metabolism and mitochondrial biogenesis [357–359]. However, some studies have found the opposite. Hu et al. [360] discovered that PGC-1α expression remained unchanged in both patients with HF and in mouse samples. Besides, overexpression of PGC-1α may induce uncontrolled mitochondrial proliferation in the cardiac ventricle and lead to cardiac dysfunction, contingent upon the restoration of mitochondrial biogenesis [361]. As a double-edged sword, the balance between excessive expression treatment of PGC-1α in maintaining mitochondrial biogenesis and cardiac function remains unclear.

Mitochondria are tightly packed between myofibrils in the adult cardiac myocytes, and therefore, mitochondrial dynamics were thought to be insignificant in this subpopulation [362]. However, recent studies have shown that mitochondrial fusion events exist in cardiomyocytes, as evidenced by rapid content mixing events between adjacent organelles and slower events between both neighboring and distant mitochondria, and are associated with cardiac contractility [363]. Mitochondrial dynamics is impaired in HF. Ablation of the *OPA1* gene is embryonically lethal, and heterozygous mice exhibit impaired mitochondrial function, susceptibility to hemodynamic stress, and decreased function in the heart, although it can survive [364, 365]. In humans and rats with HF, the reduced expression of OPA1 in the cardiomyocytes is characterized by small and fragmented mitochondria, which may be due to an imbalance between L-OPA1 and S-OPA1, the release of cytochrome C and deacetylation levels [80, 364, 366, 367]. Decreased mitochondrial fusion proteins MFN1/2 have been in the myocardium of mice with HF [368]. Mid-gestational deletion of MFN1/2 resulted in dilated cardiomyopathy and HF in mice on day 7 postnatal, accompanied by a decrease in mitochondrial biogenesis and mitophagy [369]. Yue and colleagues [370] found that prolonged activated Yes-associated protein 1 disrupted mitochondrial dynamics and triggered mitochondrial dysfunction by targeting Dnm1l and MFN1 in a mouse model of abdominal aortic constriction-induced HF, resulting in cardiac hypertrophy, and this process was also related to impaired mitochondrial biogenesis. However, the effects of knockout of MFN1/2 alone on cardiac function in mouse cardiac myocytes remain controversial [371–373]. Similarly, DRP1 was upregulated in HF patients and mammal models [374–376]. With the treatment of hypertrophic agonist norepinephrine in neonatal rat cardiomyocytes, mitochondrial fission was enhanced followed by a decrease in mitochondrial function. Mechanistically, norepinephrine increased cytoplasmic Ca^2+^ to activate calcineurin through targeting α_1_-adrenergic receptors, promoting DRP1 migration to mitochondria, involving dephosphorylation of DRP1 at Ser637 [377]. Interestingly, Xu et al. [378] demonstrated that chronic stimulation of β-adrenergic receptors by isoproterenol-induced mPTP openings and mitochondrial damage in failing hearts through activating CaMKII followed by phosphorylation of DRP1 at Ser616, independent of phosphorylation at Ser637. Pharmacological inhibition of DRP1 can reverse disordered mitochondrial dynamics and cardiac hypertrophy [378, 379]. However, DRP1 deficiency still does not favor mitochondrial health and cardiac function, due to damaged mitophagy and PCD changes, which seem to be associated with the duration of DRP1 deletion [380, 381]. Recently, Donnarumma et al. [382] found that mitochondrial inner membrane protein mitochondrial fission process 1 affected cardiac pathological events by targeting mPTP and mitochondria uncoupling of the inner membrane, with no effect on mitochondrial division.

There is growing evidence suggests that maladaptive mitophagy exacerbates mitochondrial dysfunction and HF [85, 383, 384]. Given the core role of PINK1-mediated MFN2 phosphorylation, which induces the localization of Parkin on the mitochondrion, the PINK1/Parkin pathway is essential for proper MQC. Decreased protein levels of PINK1 and Parkin have been observed in HF samples from patients and mouse models [383, 385]. Supporting these data, PINK1^−/−^ mice exhibit left ventricular dysfunction and cardiac hypertrophy at 2 months of age, accompanied by increased oxidative stress and damaged mitochondrial function [385]. Mechanistically, the isoform switch from AMPKα2 to AMPKα1 during HF resulted in the inhibition of PINK1/Parkin/SQSTM1-mediated mitophagy, aggravating mitochondrial dysfunction and severe cardiomyocyte apoptosis via targeting PINK1 dephosphorylation [383]. Correspondingly, specific activators of AMPKα2 may be a potential clinical translational therapy to attenuate HF. Interestingly, mitophagy was transiently increased during early pressure overload as a protective role, depending on the temporary activation of conventional mitophagy and ULK1-dependent alternative mitophagy [386]. Pharmacological inducer of PINK1/Parkin-mediated mitophagy has also been proven to mitigate cardiac function [384, 387]. However, Chaanine et al. [388] found that in the case of pressure overload, JNK signaling induced activation of BNIP3-dependent mitophagy and impaired cardiomyocytes, exacerbating HF. Therefore, it is crucial to identify the proper timing and method for targeting mitophagy in the treatment of HF.

#### Myocardial ischemia/reperfusion (I/R) injury and myocardial infarction (MI)

Acute MI is a major cause of death and disability worldwide. The absence of oxygen and nutrient supply at acute myocardial ischemia, excess oxidative stress, and mPTP opening at myocardial reperfusion result in severe biochemical and metabolic disorders in the cardiomyocytes, finally contributing to mitochondrial dysfunction and cardiomyocyte death [389, 390]. During these processes, mitochondrial morphology, and function change dramatically, implying that the MQC system may play important roles in MI.

The continuous renewal of mitochondria through mitochondrial biogenesis enables cardiomyocytes to rapidly adapt to new energy demands during I/R injury and MI. Recently, a full-length transcriptomic analysis in mice revealed that cardiac PGC-1α coding transcripts were decreased during I/R injury, which was associated with the infarcted area [391]. Several studies have suggested that cardiac PGC-1α expression and mitochondrial biogenesis were downregulated in mammal models of MI [392, 393]. In hypoxia/reoxygenation (H/R)-treated cardiomyocytes, the activity of AMPK was decreased with subsequent inhibition of TFAM and Nrf2, while melatonin treatment reactivated the AMPK/PGC-1α pathway to restore mitochondrial biogenesis [394]. PGC-1α-induced mitochondrial biogenesis also participated in cardiac H/R injury by regulating mitochondrial fusion [394]. Sirt1 enhances PGC-1α activity through deacetylation. It has been reported that the disruption of the Sirt1/PGC-1α pathway and increased apoptosis in cardiomyocytes was caused by I/R incubation [395]. Furthermore, due to the relationship between PGC-1α and energy metabolism, activation of the Sirt1/PGC-1α pathway also increased glucose uptake and pyruvate metabolism in cardiomyocytes [395]. It is certain that increased PGC-1α activity is conducive to maintaining mitochondrial homeostasis and alleviating cardiac dysfunction during I/R injury [394–397].

Under I/R stimulation, cardiomyocytes exhibit a shift toward mitochondrial fission and a loss of mitochondrial fusion, which in turn induces mPTP opening, ROS production, and eventually cardiomyocyte death [389]. The expression and activity of DRP1 increase significantly during MI or I/R, whereas pharmacological inhibition of DRP1 protects the heart from I/R injury and reduces infarct size [398–400]. Phosphorylation of Ser616 and dephosphorylation of Ser637 on DRP1 is related to mitochondrial fission. I/R injury induces dephosphorylation of DRP1 at Ser637 through upregulating PGAM5 and calcineurin [401, 402], while the activation of Rho-associated coiled-coil containing protein kinase 1 (ROCK1), cyclin-dependent kinase 1 (CDK1), protein kinase C isoform delta (PKCδ) and glycogen synthase kinase-3β (GSK-3β) or inhibition of Sirt3-AMPK pathway can simultaneously increase phosphorylation at Ser616 [402–405]. Besides, decreased miR-499 during MI enhances calcineurin-mediated dephosphorylation of DRP1 at Ser656, which induces mitochondrial fragmented and apoptosis [406]. More recently, Sun et al. [407] found that TBC domain family member 15 (TBC1D15) declined after myocardial I/R injury. It was shown to maintain mitochondria-lysosome contacts to regulate asymmetrical mitochondrial fission via TBC1D15-DRP1 interaction, promote autophagy flux, and preserve mitochondrial homeostasis. The DRP1 receptor MFF also plays a critical role in the regulation of mitochondrial fission following cardiac I/R injury [111]. Cardiac-specific DRP1 knockout mice exhibited mitochondrial elongation and were more susceptible to I/R injury [380]. Therefore, moderate control of mitochondrial fission balance is critical for protecting the heart against I/R injury. In contrast to mitochondrial fission, mitochondrial fusion usually participates in the maintenance of mitochondrial health and physiological function as a protective mechanism. After I/R injury, mitochondrial fusion regulator OPA1 is expressed abnormally [408, 409]. It has also been reported that the expression of OPA1 is decreased in the patient’s hearts with ischemic cardiomyopathy [366]. MCU is a unidirectional channel that controls Ca^2+^ inflow into mitochondria [408]. Guan et al. [408] found that MCU was upregulated during myocardial I/R, leading to mitochondrial calcium overload and activation of calpain, which subsequently inhibited OPA1-mediated mitochondrial fusion and mitophagy. The imbalance of L-OPA1 and S-OPA1 caused by incremental OMA1 also plays an important role during I/R injury [410]. The AMPK-OPA1-mitochondrial fusion/mitophagy axis is disrupted during cardiac I/R injury and contributes to mitochondrial stress and caspase-9-involved mitochondrial apoptosis, while this process can be reversed with the treatment of melatonin [411]. Unlike the effects of OPA1, the role of MFNs in cardiac I/R injury remains controversial. On the one hand, increasing the expression of MFN1/2 has been shown to protect cardiomyocytes against I/R injury, by facilitating mitochondrial fusion and suppressing mPTP opening [398]. The proteasome is responsible for MFN2 degradation while inhibiting proteasome can partly preserve MFN2 to retain mitochondrial integrity and protect against I/R injury [412]. Zhou et al. [413] recently demonstrated that MORN repeat-containing protein 4 exerts endogenous protection in ischemic cardiomyocytes by binding to MFN2 and promoting MFN2 phosphorylation at Ser422 in the ROCK2 complex. On the other hand, unexpectedly, dual ablation of MFN1 and MFN2 in acute hearts leads to MI resistance, even with mitochondrial dysfunction [414]. Similar phenomena have also been observed in mouse cardiomyocytes with single MFN2 deletion [415]. These observations may be related to the extra-mitochondrial pleiotropic actions of MFNs. MFN2 acts as a rope between mitochondria and ER [416]. In the heart, loss of MFNs under I/R stimulation disrupts the interactions between mitochondria and ER, thus attenuating mitochondrial Ca^2+^ overload and suppressing mPTP opening [414]. However, long-term deletion of MFNs is detrimental to cardiomyocytes, and may rapidly progress to dilated cardiomyopathy and cardiac death [417]. Given the diverse functions of proteins involved in mitochondrial dynamics, more experiments are needed to explicate the roles of these proteins during MI.

Recent evidence has demonstrated that cardiac I/R injury resulted in dysregulation of mitophagy. Cardiac Parkin deficiency makes mice much more sensitive to MI surgery but does not affect mitochondrial or cardiac function under physiological conditions [418]. In response to ischemic stress, PINK1/Parkin-mediated mitophagy is induced in both vivo and in vitro, although other studies have reported the opposite [419]. Increasing Parkin-related mitophagy during ischemia appears to be a cardioprotective event, partly because the Parkin-mitophagy pathway inhibits the opining of mPTP and cardiomyocyte necroptosis [418, 419]. However, the role of PINK1/Parkin-mediated mitophagy in cardiac I/R remains complicated and not yet fully understood. Several studies have revealed that mitophagy played a protective role in response to I/R stimulation. Mice lacking PINK1 are more vulnerable to I/R injury due to worsening mitochondrial function [420]. mPTP opens during reperfusion, while PINK1/Parkin‐mediated mitophagy inhibits this opening [420, 421]. Sun et al. [422] found that Parkin catalyzed the ubiquitination of cyclophilin-D (CypD) to block mPTP opining, thereby preventing cardiomyocytes from programmed necrosis and I/R injury, in addition to regulating mitophagy. Several drugs or compounds that pharmacologically activate PINK1/Parkin-mediated mitophagy have also been shown to have cardioprotective effects against I/R injury [423–425]. Conversely, excessive PINK1/Parkin-mediated mitophagy is detrimental during cardiac I/R. The PINK1/Parkin pathway is activated by I/R injury in vivo and in vitro, whereas knockdown or inhibition of Parkin protects cardiomyocytes from mitophagy and apoptosis [426, 427]. Notch1 signaling physiologically regulates cardiac development and cardiomyocyte proliferation, alleviates mitophagy and mitochondrial fragmentation by suppressing the PTEN/PINK1 pathway, and protects the heart from I/R injury [428]. Furthermore, it has been reported that melatonin downregulated the expression of mitophagy-related proteins (Parkin, Beclin1, and NIX) and diminished excessive mitophagy through the MT2/Sirt3/FoxO3a signaling pathway, thus attenuating H/R injury in H9c2 cells [429]. Notably, there is crosstalk between each MQC, and mitochondrial fission is often regarded as to be upstream of mitophagy. As a result, targeting mitochondrial dynamics-related proteins seems to attenuate myocardial I/R injury by controlling mitophagy [411, 430]. FUNDC1-mediated mitophagy plays a beneficial role in cardiac I/R injury. FUNDC1-mediated mitophagy in cardiomyocytes is induced by hypoxia challenge but inhibited during reperfusion [431, 432]. At the molecular level, I/R injury upregulates the expression of CK2α, which contributes to FUNDC1 phosphorylation at Ser13 and subsequent inhibition of FUNDC1-mediated mitophagy [432]. Similarly, elevated RIPK3 also inhibits mitophagy following I/R injury via post-transcriptional modification of the FUNDC1 phosphorylation site [433]. Mao et al. [432] recently found that cardiac I/R stimulation suppressed the expression of Polo-like kinase 1 (PLK1), and thus counteracted the induction of PLK1 and FUNDC1-dependent mitophagy [434]. Impaired FUNDC1-mediated mitophagy hinders the clearance of damaged mitochondria induced by I/R injury, thus facilitating mitochondrial apoptosis and impairing cardiac function. Furthermore, FUNDC1-dependent mitophagy in platelets is associated with platelet activation and cardioprotective effect during I/R injury [431, 435]. A recent study revealed that FUNDC1-mediated mitophagy can maintain MQC and alleviate myocardial I/R damage by activating the mitochondrial unfolded protein response (UPR^mt^) [180]. It has been reported that mitophagy is enhanced through the HIF-1α/BNIP3 signaling pathway, and berberine could further induce this process to protect against myocardial I/R injury [436, 437]. However, Jin et al. [111] found that downregulation of dual-specificity protein phosphatase1 after cardiac I/R injury promotes excessive BNIP3-mediated mitophagy via the JNK pathway and resulted in cardiomyocyte death. Additionally, vitamin D-mediated cardio protection against I/R injury is related to the inhibition of BNIP3-mediated mitophagy and apoptosis [438]. Due to its additional involvement in apoptosis, the effects of BNIP3 and its dependent mitophagy on cardiac I/R injury remain uncertain. Proper regulation of mitophagy may efficiently remove damaged mitochondria, but excessive or insufficient mitophagy appears to aggravate I/R injury. Therefore, potential methods for maintaining baseline mitophagy following I/R injury warrant further research.

#### Atherosclerosis

Atherosclerosis is a chronic and progressive vascular disease based on inflammation that predisposes patients to MI, ischemic cardiomyopathy, strokes, and peripheral arterial disease [439]. Convincing evidence has revealed the essential effects of MQC acting in the pathogenesis of atherosclerosis.

Endothelial cell damage induced by ROS is the primary event of atherosclerosis [440]. After oxidized low-density lipoprotein (ox-LDL) treatment in human aortic endothelial cells (HAECs), the expression of PGC-1α is inhibited, accompanied by mitochondrial energy metabolism disorder, increased mtROS and apoptosis [440]. Consistent with this, PGC-1α protein levels were also downregulated in Ang-II-induced atherogenesis in ApoE^−/−^ mice [441]. Karnewar et al. [441] reported that mitochondria-targeted esculetin reduced oxidative stress and increased mitochondrial biogenesis, exhibiting an anti-atherogenic effect in endothelial cells as well as in mouse models.

Endothelial dysfunction is conducive to the development of atherosclerosis in patients with diabetes due to altered mitochondrial dynamics and subsequent increased ROS, as evidenced by higher FIS1 expression and lower mitochondrial network extent [442]. Consistently, mitochondrial fragmentations were observed by electron microscopy in diabetic mouse aortic endothelial cells [443]. Liu et al. [444] found that DRP1 knockdown in human umbilical vein endothelial cells (HUVECs) alleviated ox-LDL-induced mitochondrial fission and apoptosis. Retinol binding protein 4, an IR-related adipokine highly expressed in the serum of patients with metabolic syndrome, disrupts the homeostasis of mitochondrial fusion and fission in endothelial cells, resulting in higher levels of DRP1 and FIS1 as well as lower levels of MFN1 [445]. Treatment with metformin can improve endothelial function and reduce atherosclerotic lesions in diabetic ApoE^−/−^ mice by regulating DRP1-mediated mitochondrial fission [443]. Mitophagy is also implicated in the development of atherosclerosis. Under high-glucose conditions, PINK1/Parkin-mediated mitophagy was impaired in endothelial cells, leading to mitochondrial dysfunction and apoptosis [446]. Furthermore, Xia et al. [350] demonstrated that ox-LDL challenge upregulated the expression of PTEN, which suppressed mitophagic flux through the AMPK-CREB-MFN2 pathway, leading to apoptosis in HUVECs. On the contrary, higher levels of nuclear receptor subfamily 4 group A member 1 (NR4A1) induced by ox-LDL in endothelial cells triggered excessive Parkin-dependent mitophagy, whereas NR4A1 deletion was shown to protect endothelial cells from energy metabolism disorder and apoptosis [447]. In addition to endothelial cells, the phenotypic transformation of vascular smooth muscle cells (VSMC) plays a critical role in the pathogenesis of atherosclerotic plaque formation. Ox-LDL may lead to lipid deposition and foam cell formation in VSMC. During this process, VSMC exhibits mitochondrial over-fission and decreased mitochondrial branch length, which can be reversed by midiv-1 [448]. Similarly, platelet-derived growth factor (PDGF), involved in recruiting VSMCs to the neointima and promoting atherosclerosis development, promotes mitochondrial fragmentation and reduction of MFN2 in VSMCs [449]. Wang et al. [450] also reported that silencing DRP1 could inhibit VSMC migration induced by PDGF and decrease pathological intimal hyperplasia in mice. In an atherosclerosis mouse model, apelin-13 promoted PINK1/Parkin-mediated mitophagy of VSMCs via activating AMPKα thereby aggravating the progression of atherosclerotic lesions [451]. The authors also found that apelin-13 alters the balance of mitochondrial dynamics (increasing DRP1, decreasing MFN1, MFN2, and OPA1), inducing a proliferation phenotype in VSMCs [451].

Ox-LDL stimulation promotes mitochondrial dysfunction and inflammatory response in monocyte-macrophage, which participates in chronic inflammatory diseases, including atherosclerosis. Mechanically, increased methyltransferase-like 3 induced by ox-LDL coordinates with YTHDF2 to suppress the expression of PGC-1α [452]. A recent study reported that enhanced DRP1-mediated mitochondrial fission induced macrophage M1 polarization and foam cell formation through the mito-ROS/NLRP3 inflammasome singling pathway, hence accelerating atherogenesis [453]. Macrophage apoptosis is considered to be a critical step in the formation of a pro-thrombotic necrotic core. Enhancing CD137 (a kind of T cell co-stimulatory molecule) signaling facilitates the progression of atherosclerotic plaque in the ApoE^−/−^ mice, where elevated TUNEL co-staining with the CD68 marker is observed. At the molecular level, CD137 signaling can induce mitochondrial fission through the p38 MAPK pathway in mouse peritoneal macrophages, resulting in mitochondria dysfunction and macrophage apoptosis [454].

Mitophagy is considered to protect the stability of macrophage function. It has been reported that inhibiting caspase 1 antagonizes NLRP3 inflammasome assembly, prevents foam cell formation and pyroptosis in macrophages, partly by boosting Parkin-mediated mitophagy and efferocytosis, and thereby ameliorates vascular inflammation and atherosclerosis [455]. This study demonstrated that targeting the interplay between NLRP3 inflammasome activation and dysfunction of MQC could be a potential therapeutic strategy for atherosclerosis. Apolipoprotein A-I binding protein, recently identified as an autophagy regulator in macrophages, regulates macrophage M1/M2 polarization and performs an anti-atherosclerotic role via PINK1-dependent mitophagy [456, 457]. Notably, despite the associated beneficial metabolic effects, high-protein diets induce the formation of atherosclerotic plaque in mice. Mechanically, elevated levels of amino acid in the blood and atherosclerotic plaque activated mTORC1 in macrophages, leading to repression of downstream mitophagy and consequently exacerbating mitochondrial dysfunction and apoptosis [458].

#### Cardiac aging

Due to the significant increase in human life expectancy, aging-associated cardiovascular diseases are becoming more prevalent and raising concerns due to their substantial impact on health, quality of life, and socioeconomic burdens [459]. The aging hearts exhibit unique histological and morphological features, which are linked to the development of cardiac dysfunction [460]. Specifically, aged hearts experience impaired mitochondrial function characterized by changes in mitochondrial morphology, mPTP opening, MQC dysfunction, and ROS formation [461].

Mitochondrial biogenesis in cardiac senescence-associated mitochondrial dysfunction has attracted extensive attention. In various aging animal models, a decrease in the expression of PGC-1α in cardiac tissue was observed [462–464]. Contrastively, overexpression of PGC-1αin PGC-1α muscle-specific transgenic mice counteracted age-associated pathological changes in the heart [465]. Wang et al. [462] demonstrated that spermidine supplementation enhanced mitochondrial biogenesis and function via the Sirt1/PGC-1α signaling pathway and thus ameliorated cardiomyocyte aging in rats. Alterations in mitochondrial dynamics have also been associated with cardiac aging. Reduced levels of MFN1 and MFN2 were found in the hearts of 25-month-old rats [464]. Furthermore, Fernández-Ortiz and colleagues [466] discovered decreased protein levels of MFN2, OPA1, and DRP1 both in mature mice (12 months old) and old mice (24 months old), compared with young mice (3 months old). In contrast, upregulated expression of OPA1 and DRP1 was reported in the hearts of 36-month-old rats by Ljubicic et al. [467]. Despite the discrepancies in mitochondrial dynamics-related proteins observed across different studies, which can be attributed to dynamic changes occurring at different ages, these findings collectively indicate that there are alterations in mitochondrial dynamics during cardiac aging. Transmission electron microscopy has revealed that aging hearts exhibit a diminished capacity to regulate mitochondrial dynamics, and are more susceptible to I/R injury, which is related to the age‐related deficiency of Sirt1 and Sirt3 [468]. Treatment with melatonin has been shown to enhance the response of mitochondria dynamics during cardiac aging by upregulating mitochondria fusion and fission proteins [466]. Overall, maintaining a synthetic balance between mitochondrial fusion and fission may be a realistic strategy for preserving cardiac health throughout late life rather than targeting specific phases of mitochondrial dynamics.

Mitophagy progressively declines with age in the heart. Deletion of Parkin results in premature cardiac aging in mice, as evidenced by the accumulation of mtDNA deletion mutations, the decline in cardiac functional reserve, and increased SA-β-gal activity [469]. The foregoing process can be reversed by inducing Parkin overexpression, implying that Parkin-mediated mitophagy serves a cardioprotective role in cardiac aging. P53, a transcription factor associated with senescence signal, inhibits Parkin translocation to mitochondria by binding to the RING0 domain, hence suppressing mitophagy and enhancing cardiac aging [469]. It has been reported that Shank3 expression is increased in aging cardiac tissue, and Shank3 knockout alleviates age-induced cardiac dysfunction [470]. Mechanically, Shank3 binds with CaMKII to impede its translocation to the mitochondria, which suppresses CaMKII activation and Parkin-mediated mitophagy, further triggering cardiomyocyte apoptosis. Additionally, both RhoA and lncRNA LOC105378097 also have been found to modify mitophagy as upstream regulators affecting heart aging and cardiac dysfunction in mice [471, 472]. Gao et al. [473] recently revealed that advanced aging dampens mitophagy by reducing the expression of Parkin, microtubule-associated protein light chain 3 II, phosphorylation of p62 and TBK1, whereas Parkin overexpression could rescue cardiac aging by promoting K63-linked polyubiquitination of TBK1 to facilitate mitophagy. Together, these data suggest that mitophagy plays a beneficial role during cardiac aging.

#### Hypertension

Hypertension is one of the most common cardiovascular disorders worldwide [474]. Endothelial dysfunction of the vascular structure and activation of the sympathetic nervous system are major pathogenic factors contributing to hypertension [475]. The paraventricular nucleus (PVN) plays a crucial role in the regulation of sympathetic output and salt appetite [476], making it of pivotal importance in hypertension. Sun et al. [476] found that PGC-1α and DRP1 expression were decreased while MFN2 expression was elevated in the PVN of spontaneously hypertensive rats, alongside increased oxidative stress. This observation indicates that MQC and mitochondrial function are altered during hypertension. Certain compounds such as oleuropein and punicalagin have been shown to attenuate hypertension partially by improving mitochondrial biogenesis and dynamics [476, 477]. Microglial neuroinflammation in the rostral ventrolateral medulla is also associated with stress-induced hypertension [478]. In a rat model of stress-induced hypertension, decreased Sigma-1R (an ER chaperone protein) levels result in decreased ER-mitochondria contact and mitochondrial hyperfusion, hence inducing microglial M1 polarization [478]. Enhanced mitochondrial fission was also observed in the arterial media of angiotensin II (Ang II)-induced hypertensive mice and midiv-1 treatment prevented Ang II-induced VSMC phenotypic switching and hypertension by inhibiting mitochondrial fission and oxidative stress [479].

### MQC and metabolic disease

#### IR and diabetes mellitus (DM)

The prevalence of DM, a chronic metabolic disorder, is rapidly increasing worldwide [480]. T2DM, accounting for 90 – 95% of cases, represents the major subtype of DM, IR, often accompanied by defects in insulin secretion, is considered to be a primary contributor to the development of T2DM [481]. Given its role in energy metabolism, maintaining mitochondrial homeostasis is of crucial importance to the pathophysiology of DM.

Accumulating evidence suggests that mitochondrial biogenesis is impaired in individuals with DM. Specifically, the expression of PGC-1α is significantly reduced in the liver and skeletal muscle of diabetic mice [481–483]. The liver and muscles serve key functions in regulating blood glucose homeostasis. The imbalance between glucose release from the liver and uptake from muscle and adipose tissue contributes to the development of IR and DM [484]. In diabetic hepatocytes, suppressed PGC-1α leads to hepatic mitochondrial dysfunction and increased gluconeogenesis. However, restoring redox balance through a liver mitochondrial-targeting antioxidant nano-mitoPBN can promote mitochondrial biogenesis and enhance glucose catabolism via the AMPK/Sirt3/PGC-1α axis, effectively preventing diabetes in diabetic mice [484]. Additionally, Zhu et al. [482] demonstrated that activating the Sirt1/PGC-1α/MFN2 signaling pathway could enhance mitochondrial biogenesis and alleviate IR in the liver. Nevertheless, various evidence also suggests that PGC-1α drives gluconeogenesis in the liver, leading to increased blood glucose [485]. It has been reported that PGC-1α and TFAM protein levels are decreased in skeletal muscle of db/db mice, indicating a reduction in mitochondrial biogenesis. Catalpol can promote mitochondrial biogenesis in skeletal muscle and increase glucose uptake and ATP production, thereby ameliorating IR primarily through activation of AMPK/PGC-1α/TFAM signaling [481, 483].

DM is related to an imbalance in mitochondrial dynamics. Myotubes derived from patients with T2DM exhibited increased mitochondrial fragmentation and decreased mitochondrial content, accompanied by impaired mitochondrial lipid oxidation and respiratory capacity [486]. Further, Jheng et al. [487] found that protein expressions of DRP1 and FIS1 were significantly increased in the skeletal muscle of ob/ob mice, and inhibiting mitochondrial fission with mdivi-1 improved insulin signaling and insulin sensitivity. Muscles from obese or T2DM patients showed repression of MFN2 expression. It has been reported that liver-specific deletion of MFN2 in mice resulted in glucose intolerance and impaired insulin response [71]. Furthermore, MFN2 deficiency impairs insulin signaling and insulin sensitivity in muscle and liver tissues through mechanisms involving ROS production and ER stress [71]. A recent study demonstrated that high-intensity interval training preserved fasting blood glucose and glucose homeostasis in T2DM mice through remodeling the balance of mitochondrial dynamics (increasing MFN2, DRP1, and FIS1) as well as improving glycolipid metabolism [488]. Enhanced and sustained mitochondrial fragmentation was observed in the white adipose tissue (WAT) from ob/ob mice, with higher expression of DRP1 but lower expression of MFN2 and OPA1 [489]. Midiv-1 therapy could attenuate mitochondrial dysfunction and induce white-to-beige adipocyte transdifferentiation by promoting mitochondrial biogenesis and fusion-to-fission balance [489].

As an adaptive mechanism, increased mitophagy in individuals with pre-diabetes is considered to eliminate dysfunctional mitochondria and modulate mitochondrial oxidative stress, thereby preventing the development of T2DM. However, mitophagy is impaired and higher levels of ROS further exacerbate mitochondrial dysfunction during T2DM [490]. Compared to lean individuals, myotubes derived from T2DM patients were found to exhibit significantly lower expression of Parkin [486]. Heat shock protein (HSP) 72, a stress-inducible chaperone protein, exhibited decreased levels in the muscles of obese and T2DM patients. Drew et al. [491] discovered that deletion of HSP72 resulted in decreased respiratory capacity and IR in muscle, leading to an IR-obesity phenotype in mice. Mechanically, during mitochondrial stress, HSP72 rapidly translocates to mitochondria where it interacts with MFN2 and subsequently forms a complex with Parkin, thereby maintaining mitochondrial morphology and autophagic signaling [491]. Liver-specific deficiency of Parkin also impairs mitochondrial respiratory capacity, and results in hepatic steatosis and IR in HFD-fed mice, implying a link between mitophagy and liver IR [492]. Chronic low-grade inflammation is closely associated with T2DM. A recent clinical study found a significant decrease in mitophagy-related proteins PINK1 and Parkin within peripheral blood mononuclear cells from patients with T2DM [493]. Furthermore, Gupta et al. [494] observed that under palmitate and diabetic conditions, disturbances in mitochondrial homeostasis were accompanied by elevated inflammatory IL-1β response in macrophages. At the molecular level, palmitate increases FOXO3a acetylation and prevents its binding to the PINK1 promoter, consequently downregulating PINK1-mediated mitophagy while enhancing NLRP3 inflammasome activation [494]. Similarly, FUNDC1-dependent mitophagy is implicated in the mitochondrial function of WAT. It has been reported that FUNDC1 deletion mice fed an HFD exhibited obesity and IR, partly due to decreased mitophagy and mitochondrial quality in WAT, resulting in WAT remodeling and inflammation [198].

#### Obesity

Obesity is a high-risk factor and trigger for many diseases, including T2DM, cancer, cardiovascular disease, dyslipidemia, metabolic syndrome, liver disease, chronic kidney disease (CKD), and other diseases [495]. Epidemiological studies have shown a significant global rise in the prevalence of obesity patients, imposing an immense burden on the healthcare system in terms of managing obesity and its associated disorders [496]. In recent years, an increasing body of studies has confirmed that MQC is one of the main molecular mechanisms underlying obesity [198, 497]. This suggests that ameliorating obesity may be facilitated by reversing mitochondrial dysfunction and regulating mitochondrial quality. As described below, MQC-mediated regulation of obesity primarily stems from its influence on skeletal muscle cells and adipocytes.

Obesity is strongly related to low-grade chronic inflammation, and dysfunction of adipocyte mitochondrial is a major contributor to inflammation in adipose tissue [498]. The role of mitochondria in WAT has long been overlooked due to their limited presence. A variety of proteolytic enzymes in mitochondria, such as ClpP and Lonp1 located in the matrix, and Yme1L and m-AAA located in the inner membrane, are involved in the regulation of MQC [499]. Previous studies have confirmed that OMA1, the metalloprotease, directly regulates and deactivates the motility-related GTPase OPA1 [500, 501]. In OMA1 mutant mice, inhibition of OPA1 hydrolysis disrupts the balance between mitochondrial fusion and fission, thereby aggravating obesity and thermogenic disorders [500]. In adipocytes specifically lacking Parkin protein expression through knockout techniques, mitophagy is slightly reduced while the stability of PGC-1α is enhanced by increased NQO1 protein levels. This promotes the biogenesis of mitochondria and ultimately resists HFD and aging-induced obesity [497]. The deficiency of the FUNDC1 receptor for mitophagy in WAT leads to defective mitophagy and impaired MQC, thereby exacerbating HFD-induced obesity [198]. Therefore, active regulation of MQC in adipocytes has been found to promote metabolic homeostasis and reduce the risk of obesity, thus highlighting the important role of adipose mitochondria.

Skeletal muscle, which constitutes a significant portion of the body, plays a crucial role in regulating energy balance by efficiently utilizing fat and glucose. Mitochondria are highly abundant in skeletal muscle, and any impairment in their function directly affects skeletal muscle performance, potentially leading to chronic diseases such as diabetes, obesity, and aging [194]. Among the elderly population, there is a considerable number of obese individuals who also suffer from sarcopenia, known as sarcopenic obesity, further exacerbating physiological decline [502]. Previous research has shown that the administration of the mitochondrial uncoupler BMA15 can enhance mitochondrial function and attenuate age-related loss of muscle mass and function by activating MQC. This protective effect against sarcopenic obesity was observed in obese mice at 80 weeks [503]. Furthermore, an intervention study involving 81 elderly obese patients showed that high-intensity interval training combined with l-citrulline, a potential pharmacological agent for enhancing mitochondrial and muscle function, as well as adipose tissue metabolism based on findings from aged rodent model, may increase mitophagy, mitochondrial fusion, and mitochondrial biogenesis, resulting in increased muscle strength and reduced adipose tissue accumulation [504]. Maintaining the balance of MQC in skeletal muscle also contributes to the reduction of dietary obesity. The skeletal muscle-specific knockout of FUNDC1, a mediator of mitophagy, can lead to defective mitophagy and impaired mitochondrial energy production. However, it induces a retrograde response that upregulates the expression of FGF21, promoting thermogenic remodeling of adipose tissue, protecting against HFD-induced obesity, and enhancing glucose tolerance and insulin sensitivity [505]. Muscle-specific brain-derived neurotrophic factor increases the mitochondrial content of skeletal muscle, enhances mitochondrial fission and mitophagy, and maintains the balance of MQC in skeletal muscle, thereby reducing dietary obesity in mice [506]. In summary, targeting MQC can reduce dietary obesity and age-related sarcopenic obesity by regulating the communication between skeletal muscle and fat tissues.

### MQC and nervous system disease

**Fig. 5:**
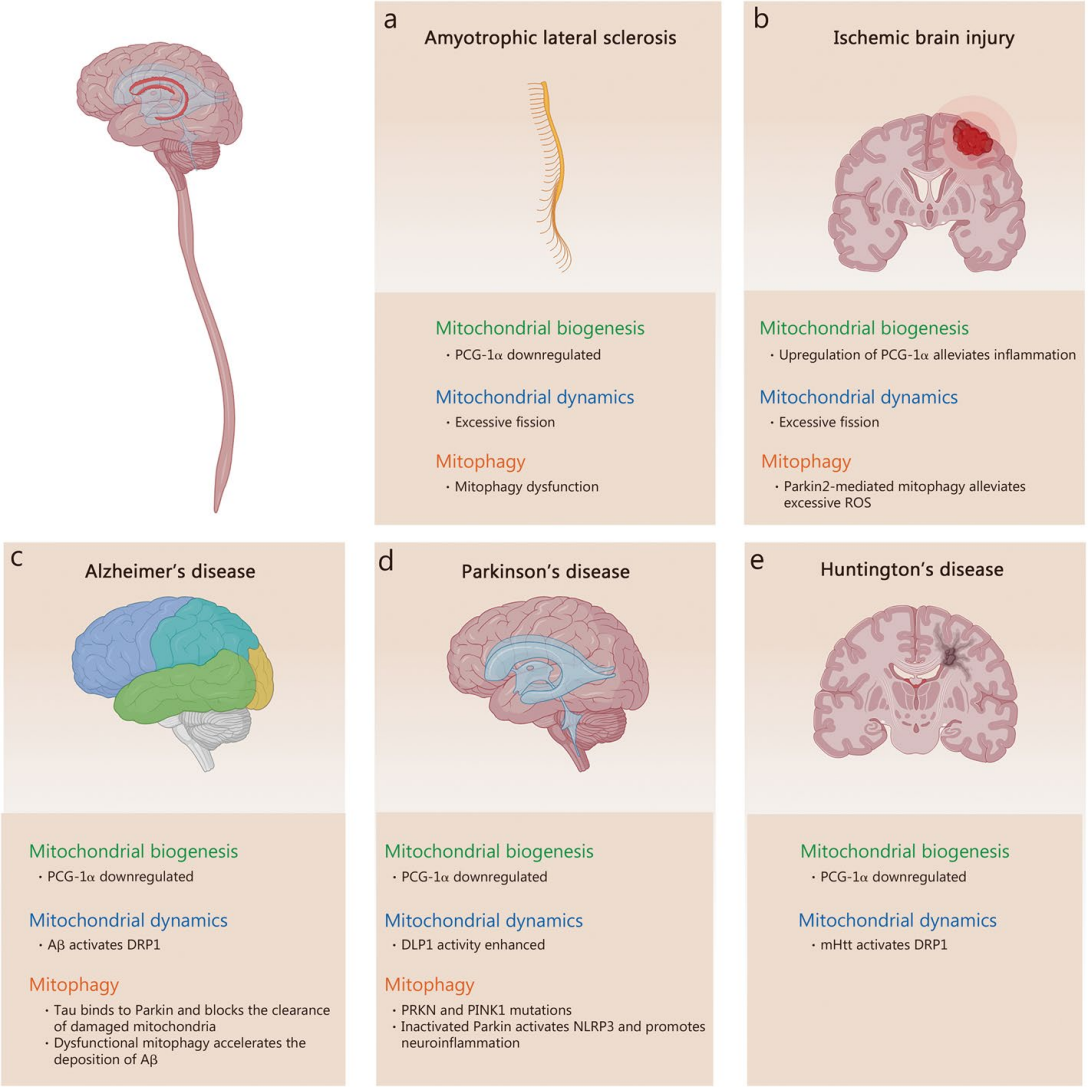
Mitochondrial quality control and nervous disease. **a** In amyotrophic lateral sclerosis, a downregulation of PGC-1α mediates a decrease in mitochondrial biogenesis, excessive mitochondrial fission, and impaired mitophagy, which are key features of the disease. **b** Ischemic brain injury exhibits similar mitochondrial phenotypic characteristics, and mitigating brain tissue damage and oxidative stress levels can be achieved through upregulation of mitochondrial biogenesis mediated by PGC-1α and mitophagy mediated by Parkin-2. **c** Accumulated Aβ in Alzheimer’s disease activates DRP1, leading to impaired mitochondrial dynamics. Additionally, Tau can interact with Parkin, affecting the clearance of damaged mitochondria. Dysfunctional mitophagy further accelerates Aβ accumulation, forming a positive feedback loop of mitochondrial damage. **d **Mutations in PRKN and PINK1 have been recognized as important genetic factors in Parkinson’s disease, and impaired mitophagy can further activate NLRP3, inducing neuroinflammation. **e** The key feature of Huntington's disease is the activation of DRP1 by mHtt, triggering severe mitophagy. Created by Biorender.com, accessed on 25 Aug 2023. DRP1 dynamin-related protein 1, mHtt mutant Huntingtin, PGC-1α PPAR-γ coactivator-1α, PINK1 PTEN-induced kinase 1, ROS reactive oxygen species

Neural cells require high concentrations of ATP to maintain their normal function, implying a need for a robust rate of mitochondrial turnover. Dysregulation of mitochondrial homeostasis is involved in various neurological disorders, in which the disruption of MQC is a central factor. Below, we discuss the relationships between MQC and common neurological disorders (Fig. 5).

#### Alzheimer’s disease (AD)

AD is the most prevalent neurodegenerative disorder characterized by deposition of Tau and Aβ [507]. Previous evidence showed that mitochondrial disruption in neurons is concentrated near Aβ [508], indicating the involvement of mitochondrial dysfunction in the progression of AD. More studies have revealed that impaired mitochondrial function in neurons precedes Tau and Aβ deposition, while Tau and Aβ in turn promote mitochondrial dysfunction, thus constituting a vicious cycle [509–511]. Tau can bind to Parkin and block its translocation to depolarized mitochondria, thereby inhibiting the clearance of damaged mitochondria [512]. In early synaptic distribution defects observed in AD, Parkin-mediated mitophagy is widely activated in neurons with Tau lesions, accelerating the turnover of mitochondrial Rho GTPase1 and preventing mitochondrial flow to the synapses for replenishment [513]. Therefore, it is crucial to investigate specific mechanisms underlying Tau-influenced mitophagy at different stages or sites of AD pathology for more precise therapeutic targets. Moreover, maintaining mitochondrial protein homeostasis through mitophagy can effectively delay the deposition of Aβ [514]. As a source of Aβ, elevated APP-CTF within neurons is also associated with impaired mitophagy in early AD [515]. Nitric oxide released from Aβ induces S-nitrosylation of DRP1, leading to excessive mitochondrial fission and neuronal synaptic damage, and aggravating AD progression [516]. Further, Aβ interacts with LONP1 inhibiting protease activity, leading to disruption of mitochondrial proteostasis and dysfunction [517]. These findings suggest a broad interaction between Aβ/Tau and impaired MQC in AD, and targeting the MQC may be a promising therapeutic intervention. In AD, there is impaired mitochondrial biogenesis due to decreased levels of PCG-1α, a transcriptional regulator essential for mitochondrial biogenesis in AD [518]. Moreover, genetic factors such as APOE 4 carrier population, may also contribute to the dysregulation of MQC, leading to impaired mitochondrial biogenesis and dynamics, and an increased susceptibility to AD [519].

#### Parkinson’s disease (PD)

PD is a neurodegenerative disease characterized by a unique motor phenotype and pathological features, including the presence of Lewy bodies and loss of nigra neurons [520]. Growing evidence emphasizes the significance of MQC in PD pathogenesis. Loss of function mutations in PRKN and PINK1 are the most common genetic factors associated with early-onset hereditary PD, as they disrupt mitophagy and lead to the accumulation of damaged mitochondria in neurons [521, 522]. Of note, genetic forms of PD extend beyond Parkin and PINK1 mutations; for example, mutations in VPS35 can enhance its interaction with DLP1, thereby promoting turnover of the mitochondrial DLP1 complex through transport via mitochondria-derived vesicles to lysosomal degradation, ultimately promoting mitochondrial breakage [523]. Moreover, Parkin inactivation results in the accumulation of PARIS, a Parkin-interacting substrate, which represses PGC-1α promoter activity, and may further inhibit mitochondrial biogenesis [524]. Impaired mitophagy accompanied by subsequent release of mtDNA triggered by PINK1/Parkin deficiency may be involved in the process of neuroinflammation. This has been demonstrated in a cohort study by Borsche et al. [223], who found that both IL-6 and mtDNA were significantly upregulated in the plasma of PINK1/Parkin-associated PD patients. Moreover, a recent study revealed that Parkin normally inhibits inflammasome priming through ubiquitination-mediated targeting and proteasomal degradation of NLRP3; however, under conditions associated with PD, inactivated Parkin leads to inflammasome activation and promotes neuroinflammation [525]. These findings suggest that impaired MQC not only causes cellular energy defects in neurological diseases but may also be involved in neuroinflammation and broader pathological processes.

#### Huntington’s disease (HD)

HD is an autosomal dominant inherited progressive neurodegenerative disorder characterized by chorea and dystonia, incoordination, cognitive decline, and behavioral challenges [526]. Mutant Huntingtin (mHtt) disrupts the function of PINK1, leading to a decrease in the targeting of mitochondria to autophagosomes and promoting the fusion between damaged mitochondria [527]. A recent study discovered that abnormal binding of mHtt to ULK1 and BECN1 is a consequence of failed autophagy. In mice and humans with HD, mHtt abnormally interacts with the mitochondrial DRP1, stimulating its enzymatic activity and causing excessive mitochondrial fission [528]. Another study reported that p53 plays a role in this process by binding to DRP1 and promoting subsequent mitochondrial fission [529]. Moreover, excessive mitochondrial fission mediated by DRP1 increases the susceptibility of HD cells to apoptosis [530]. Protective mechanisms against excessive mitochondrial fission also exist in HD patients; for example, upregulated Sirt3 may mitigate it by downregulating the expression of DRP1 and FIS1 [531]. In addition, multiple studies suggest PPAR-δ and PCG-1α are repressed in HD, which may further suppress mitochondrial biogenesis [532–534]. Conversely, increasing the activity of PCG-1 and PPAR-δ is associated with an increment of mitochondrial mass and neuroprotection [535, 536]. Moreover, defective mitophagy within MQC leads to insufficient clearance of the damaged mitochondria as well as release of mtDNA from these damaged mitochondrial will lead to innate immune activation, thus aggravating neuroinflammation in HD patients [537].

#### Amyotrophic lateral sclerosis (ALS)

ALS, also known as motor neuron disease, causes progressive denervation of autonomic muscles due to degeneration of upper motor neurons in the motor cortex and lower motor neurons in the brain stem and spinal cord. Multiple genetic alterations are involved in the disease progression of ALS, such as SOD1. In a mouse model associated with SOD1 mutation-induced ALS, elevated levels of PCG-1α in muscle stimulate mitochondrial biogenesis, which helps counterbalance some of the observed mitochondrial dysfunction in the disease. However, despite maintained mitochondrial activity and muscle function at end-stage of ALS, survival is not extended [538]. Moreover, damaged mitochondrial fusion can further lead to defective mitochondrial in SOD1 mutation motor neurons [539, 540]. In neurons from early ALS patients, damaged mitochondria are cleared by Parkin-mediated mitophagy, however, unabated mitophagy along with degradation of mitochondrial proteins and decreased mitochondrial biogenesis can accelerate neurodegeneration by causing mislocalization and depletion of mitochondria [541]. This process is subject to complex regulation under ALS conditions involving multiple molecules, including mutational factors and aberrant activation of signaling pathways. A recent study indicated that mutant TBK1 in ALS also contributes to disrupting mitophagic flux, inhibiting the clearance of damaged mitochondria [542]. The overactivation of the ERK1/2 pathway and subsequent increased levels of translocator protein also contribute to impaired mitophagy [543]. Ubiquitinated transactive response DNA-binding protein-43 kD (TDP-43) is a main pathological hallmark of ALS, primarily localized in the cytoplasm of the brain and spinal cord, while it normally resides in the nucleus under physiological conditions. TDP-43 accumulation induces mitochondrial dysfunction through multiple processes, including disruption of MQC, and binding to Parkin pre-mRNA, inhibiting Parkin translation levels [544]. TDP-43 deficiency can also lead to the downregulation of Parkin [545], suggesting that these different mechanisms may coexist rather than exclude each other during mitophagy dysregulation at different stages of ALS progression. Axonal accumulation of TDP-43 can inhibit the local translation of nuclear-encoded mitochondrial genes, further impairing mitochondrial biogenesis in axon [546]. Abnormal mitochondrial dynamics contribute to disease progression in ALS, hyperactivated DRP1 leads to increased mitochondrial fragmentation and exacerbates the progression of ALS, while negative modulation or suppression of DRP1 expression could rescue cell viability [547].

#### Ischemic brain injury

In the context of I/R brain injury, upregulated PGC-1α in microglia promotes mitophagy, thereby alleviating ROS-mediated NLRP3 inflammasome activation, and in turn exerting neuroprotective effects [548]. Parkin-mediated mitophagy also alleviates excessive ROS production and may underlie the neuroprotective effects exerted by ER stress [549, 550]. In addition, BNIP3/NIX and FUNDC1-mediated mitophagy are involved in neuroprotection during I/R brain injury and can function independently of PARK2 [140, 551]. A previous study pointed out that excessive mitophagy activated by BNIP3 in ischemic brain injury without reperfusion could lead to neuron death [143]. Mitochondrial fission is an upstream process tightly linked with mitophagy, thus maintaining mitochondrial fission can promote mitophagy and protect against nerve damage caused by ischemic stroke [552]. Additionally, hyperglycemia may exacerbate mitochondrial fission under cerebral I/R conditions, further aggravating neural injury [553]. Activation of the N-Methyl-D-Aspartate (NMDA) receptor triggers Ca^2+^ influx, which is called excitotoxicity in stroke, acting as a signaling hub of neuronal pro-death events. A previous study revealed that excitotoxicity inhibits the expression of MFN2, leading to mitochondrial fragmentation and cell death [554]. This suggests that the regulation of mitochondrial dynamics from division to fusion plays a protective role in ischemic brain injury, which was confirmed by a previous study showing that inhibition of DRP1 relieved ischemic brain injury [555].

### MQC and pulmonary disease

**Fig. 6:**
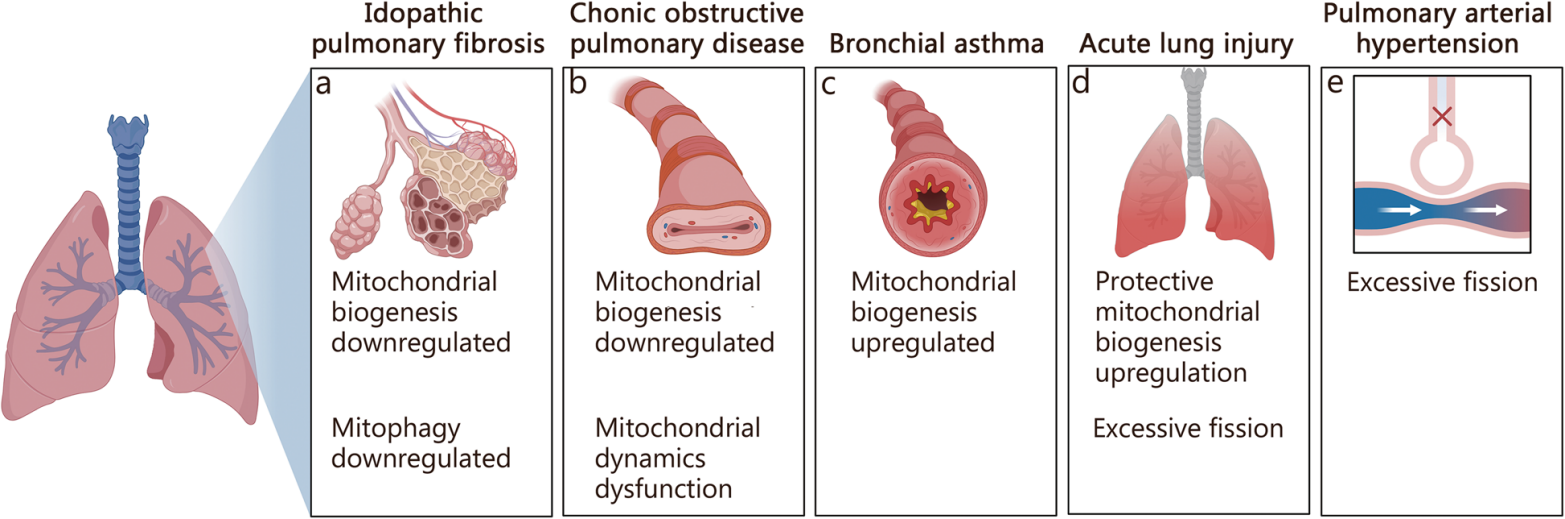
Mitochondrial quality control and pulmonary disease. **a** In idiopathic pulmonary fibrosis, mitochondrial quality control dysfunction manifests as reduced mitochondrial biogenesis and downregulation of mitophagy. **b** Similarly, in chronic obstructive pulmonary disease, diminished mitochondrial biogenesis and impaired mitochondrial dynamics are observed. **c** Conversely, in bronchial asthma, mitochondrial biogenesis is upregulated, which is closely associated with the high demand for smooth muscle cell proliferation. **d** Notably, excessive mitochondrial fission is a key feature of acute lung injury, where increased mitochondrial biogenesis is beneficial. **e** Likewise, excessive mitochondrial fission is also a notable feature of pulmonary arterial hypertension. Created by Biorender.com, accessed on 25 Aug. 2023

Mitochondria play a crucial role in the regulation of redox and immune responses, exerting influence on processes such as ROS production, activation of inflammasome, cellular proliferation, and prevention of fibrosis. These processes are implicated in the pathogenesis of various lung diseases. Here, we delve into the vital role of MQC in lung diseases (Fig. 6).

#### Idiopathic pulmonary fibrosis (IPF)

In myofibroblasts, TGF-β1 can induce transcriptional inhibition for PINK1, and deficient mitochondrial targeting for mitophagy, contributing to the progression of pulmonary fibrosis [556]. Impaired mitochondrial in alveolar type II cells are associated with the downregulation of PINK1 and defective mitophagy, making them susceptible to apoptosis and the development of lung fibrosis [557]. Another study suggests that there is a link between ATF3 and PINK1 in the alveolar epithelium; specifically, increasing ATF3 associated with aging inhibits the transcript level of PINK1 [558]. In alveolar macrophages, ROS-induced mitophagy and apoptosis resistance contribute to the long-term production of TGF-β, promoting the progression of pulmonary fibrosis [559]. However, although mitophagy is often considered to be a protective mechanism against damage mitochondria in pulmonary fibrosis, a recent study showed that PGAM5-mediated mitophagy in turn led to a self-perpetuating escalation of mitochondrial membrane potential depolarization [560]. Although the TGF-β induced Smad1/2 pathway is well recognized as regulating mitochondrial fragments, a previous study demonstrated inactive Smad2 could bind to MFN2 and further mediate mitochondrial fusion [561]. However, further studies are needed to understand how Smad2 influences mitochondrial function in IPF. A recent study proposed that the stiff matrix in fibrotic lung promotes mitochondrial fission in fibroblasts to meet the higher energy demand [562]. Protective mechanisms also exist against lung fibrosis in vivo, for example, thyroid hormone can promote the expression of PINK1 and PGC-1α, resulting in the restoration of mitochondrial function [563]. Chung et al. [564] revealed that MFN1 and MFN2 are associated with regulating the synthesis of lipids, while loss of MFN1 and MFN2 in ACE2 exacerbates bleomycin-induced lung fibrosis.

#### Chronic obstructive pulmonary disease (COPD)

Oxidative stress-mediated mitochondrial damage is an important pathological manifestation of COPD. Depolarized mitochondria activate mitophagy through the PINK1/Parkin2 pathway to clear the damaged fraction. However, the Parkin expression is downregulated in the lungs of COPD patients, causing mitophagy deficiency, which in turn promotes cell senescence and PCD [565]. Interestingly, overexpression of Parkin is sufficient to induce mitophagy when PINK1 protein levels are reduced. Conversely, PINK1 overexpression fails to rescue Parkin downregulation-mediated mitophagy and subsequent cell senescence [566], indicating that Parkin plays a pivotal role in regulating mitophagy during COPD. Moreover, mitophagy also contributes to necroptosis in COPD [567]. In addition, impaired mitochondrial dynamics characterized by defective fission and fusion processes are observed in the lung tissues of patients with emphysema, suggesting an association between impaired mitochondrial dynamics and emphysema progression [568]. In COPD patients exposed to cigarette smoke, alveolar epithelial cells or fibroblasts partially modulate MQC in response to external stimuli by regulating the coexistence ratio of different forms of OPA1, namely L-OPA1, and S-OPA1, and under the intervention of OPA1-interacting protein SLP-2, thereby mitigating damage [569].

#### Bronchial asthma

In young children with severe wheezing, increased mitochondria biogenesis is involved in the remodeling of bronchial smooth muscle [570]. The proliferation of asthmatic bronchial smooth muscle cells mainly requires mitochondrial-depend oxidative phosphorylation, thus increased mitochondrial biogenesis is essential in BSM cells [571]. Allergen-induced ROS production modulates mitophagy by regulating OPTN, which participates in the development of allergic airway inflammation [572]. Moreover, the MQC cross-talk between different cell types plays a role in asthma. For example, epithelial-derived thymic stromal lymphopoietin may induce excessive ROS production and subsequently regulate mitophagy and mitochondrial biogenesis, further influencing M1/M2 polarization chemokine expression in monocytes [573]. IL-4 mediated accumulation of asymmetric dimethylarginine causes oxo-nitrative stress, leading to mitochondrial dysfunction and HIF-1α activation. Activation of HIF-1αdecreases mitochondrial biogenesis, resulting in an alteration of mitochondrial turnover that can lead to bronchial epithelial cell death [574].

#### Acute lung injury (ALI)

Sepsis-induced ALI is associated with mitochondrial damage, whereas activated mitophagy, removes damaged mitochondria, promoting repair of lung tissues and cell survival [575]. In rat models, heme oxygenase-1 and carbon monoxide play a protective role in lung injury by regulating mitochondrial dynamic equilibrium [576]. Importantly, *Staphylococcus aureus* infection promotes PINK1 activity, which then phosphorylation of cardiolipin synthase on mitochondria (CLS1), leading to the excessive release of cardiolipin and further aggravating ALI [577]. Many studies have shown that the impaired mitochondrial dynamic is characterized by upregulated mitochondrial fission, and resultant oxidative stress is an essential mediator of ALI, as evidenced by the upregulation of OPA1 and MFN1. Conversely, increased mitochondrial fusion can alleviate ALI [578–580]. Under conditions of ALI, lung cells initiate Nrf2-mediated protective response, such as promoting mitochondrial biogenesis to reduce oxidative stress and inflammatory response for alleviating the progression of ALI [581]. Mitophagy seems to be an aggravating factor for ALI based on previous research findings. Therefore, PINK1 interaction with the ubiquitin apparatus to enhance mitochondrial quality could limit inflammatory injury [582]. Our team has been dedicated to exploring the pathogenesis of ALI and potential therapeutic strategies [579, 583, 584]. Our latest research findings indicate that mitochondrial dysfunction in type II alveolar epithelial cells contributes to epithelial barrier disruption during ALI [583]. Mechanistically, HDAC3 exerts its regulatory function by promoting the deacetylation and nuclear translocation of FOXO1, leading to upregulation of ROCK1 transcription, which exacerbates mitochondrial damage [583]. In conclusion, targeting MQC holds significant research potential for the prevention and treatment of ALI.

#### Pulmonary arterial hypertension (PAH)

The pathological process of PAH is characterized by the proliferation of smooth muscle cells and the metabolic transition of mitochondria from oxidative phosphorylation to aerobic glycolysis [585]. Excessive proliferation of pulmonary artery smooth muscle cells (PAMSCs) is associated with mitochondrial fragmentation in human and experimental PAH models. Decreased expression of MFN2 causes excessive mitochondrial content in PAMSCs, contributing to a proliferation-apoptosis imbalance of PAMSCs [586]. The upregulation and activation of DRP1 are also required for the proliferation of smooth muscle cells to accommodate mitosis [587]. Moreover, an in vitro study found that phosphorylation of DRP1 rather than upregulation alone promotes mitochondrial fission and subsequent PAMSC proliferation [588], suggesting the involvement of additional signaling cascades in vivo. Notably, mitochondrial biogenesis does not contribute to the elevated mitochondrial content in PAMSCs, instead, downregulation of PCG-1α in PAH leads to reduced mitochondrial biogenesis as well as further downregulation of MFN2 expression [586]. There is substantial evidence indicating that the post-translational activation of DRP1 or the downregulation of MFNs is the primary driving factor contributing to aberrant cell division in the pathogenesis of PAH [589].

### MQC and kidney disease

Kidneys play essential roles in maintaining homeostasis. by actively reabsorbing large amounts of solutes in the renal tubules and collecting ducts, resulting in high energy demands. The proximal tubules are responsible for reabsorbing more than 99% of glucose, while this high energy requirement cannot be met by anaerobic glycolysis. Instead, mitochondrial oxidative phosphorylation or the production of large amounts of ATP from fatty acid oxidation is required [590]. Therefore, MQC directly determines the balance of water and electrolytes in the body.

#### Acute kidney injury (AKI)

The etiology of AKI encompasses a range of factors, such as sepsis, I/R, and exposure to toxins. The pathological process includes endothelial activation, increased microvascular permeability, and altered regional blood flow distribution resulting in areas of inadequate perfusion and local hypoxemia. Ultimately, this cascade leads to the death of certain renal tubular cells.

Numerous experimental studies have shown that mitochondrial biogenesis plays a beneficial role in AKI and its subsequent renal repair. PGC-1α, the major regulator of mitochondrial biogenesis, has been confirmed to be localized in the proximal tubule with a high energy requirement [591]. In AKI induced by various factors including LPS, cisplatin, and I/R, the expression level of PGC-1α is significantly suppressed [591, 592]. The transcription factor PPARα, downstream of PGC-1α, exhibits reduced binding activity to the retinoid X receptor (RXRα) in AKI, which hampers mitochondrial biogenesis, suggesting impaired mitochondrial function in a mouse model of cisplatin-induced AKI [591]. Renal function remains unaffected under normal conditions using PGC-1α knockout mice, but knockout mice exhibit prolonged renal recovery time after injury under septic inflammatory stress conditions [593]. Overexpression of PGC-1α in renal tubular cells promoted renal recovery from LPS-induced AKI, indicating a positive correlation between PGC-1α expression and the extent of renal repair after injury [593]. Moreover, pharmacological activation of PGC-1α accelerated renal recovery from I/R injury in mice [594].

Mitochondrial fragmentation, caused by excessive fission and/or inhibition of fusion, is thought to be a key event in mitochondrial damage and renal tubular injury during AKI [595, 596]. Silencing or pharmacological inhibition of the DRP1 expression attenuated apoptosis and mitochondrial fragmentation, suggesting that DRP1 mediates excessive mitochondrial fission and ultimately leads to mitochondrial fragmentation during AKI. It is an important player in tubular cell apoptosis [595]. Similarly, specific knockdown of DRP1 in mouse kidney proximal tubules ameliorates I/R-induced inflammation and programmed cell death in AKI, while promoting the recovery process of renal epithelial cells after injury [597]. Consistently, Bif-1 regulates the mitochondrial inner membrane by its interaction with prohibitin-2 to disrupt prohibiting complexes. This induces OPA1 proteolysis and inactivation, leading to enhanced apoptosis, and mitochondrial fragmentation in ischemic AKI [598]. These studies suggest that OPA1-mediated mitochondrial fusion plays a negative regulatory role in ischemic AKI. While MFN2 is responsible for holding mitochondria firmly together during mitochondrial fusion, and specific deficiency of MFN2 in mouse kidneys aggravates ATP depletion-mediated renal tubular apoptosis, mitochondrial outer membrane damage, and mitochondrial fragmentation [599]. However, in ischemia-related AKI, mice with MFN2-specific knockout in the proximal tubule (PT) had higher survival rates and a 5-fold increase in kidney cell proliferation [600]. In contrast to its pro-apoptotic effect observed in vitro, PT-MFN2-CKO enhances the Ras-ERK pathway to promote kidney cell proliferation and thus accelerate the repair process after kidney injury [600], this suggests that the detrimental effects of MFN2 knockdown-mediated inhibition of mitochondrial fusion are counteracted or outweighed by the ERK signaling pathway, which manifests as increased proliferation of kidney cells.

There is growing evidence supporting the important role of mitophagy in AKI and kidney repair. The flux of mitophagy increases over time following kidney injury induced by I/R, contrast agents, and cisplatin-induced AKI mouse models [241, 601, 602]. In mice with cisplatin-associated AKI, PGC-1α was found to coordinate with mitophagy in the kidney, enhancing mitophagy and thus improving AKI [603]. Increased expression of BNIP3, a mediator of mitophagy, was observed in I/R-related AKI. Reduced mitophagy through BNIP3 knockdown in vivo and in vitro resulted in the promoted accumulation of damaged mitochondrial, increased oxidative stress, and increased renal tubular cell death [601]. In contrast-associated AKI, the kidneys of PINK1 or Parkin knockout mice suffered more severe renal injury than wild-type mice, and mitophagy mediated by PINK1 or Parkin was largely eliminated, suggesting that the PINK1/Parkin pathway plays a dominant role in mitophagy [241]. Also, it was discovered that mitophagy reduces the activation of mtROS and NLRP3 inflammatory vesicles to ameliorate kidney injury in mice [241]. These discoveries underscore the beneficial role of mitophagy in AKI by reducing damaged mitochondria accumulation and mtDNA release while minimizing the disturbances to renal homeostasis under inflammatory stress through multiple pathways.

#### CKD

CKD is the result of severe kidney damage or persistent irritation, characterized by impaired regeneration of renal tubular cells and tubulointerstitial fibrosis. Decreased PGC-1α expression has been observed in the kidneys of patients with diabetic kidney disease (DKD), accompanied by a decrease in mitochondrial proteins and exosomal mtDNA [604]. Similarly, decreased PGC-1α expression has been found in human patients with DKD as well as mouse models featuring renal podocytes [605]. The long non-coding RNA taurine-upregulated gene 1 (Tug1) was shown to interact with PGC-1α, facilitating its binding to the promoter region and enhancing transcriptional activity through direct interaction with the gene encoding PGC-1α [606]. Overexpression of Tug1 transgene specifically in podocytes improved pathological damage and mitochondrial stability in a DKD mouse model, suggesting a beneficial role for PGC-1α-mediated mitochondrial biogenesis in the progression of DKD [606]. As mentioned earlier, AMPK acts as a positive regulator of PGC-1α, and pharmacological activation of AMPK followed by restoration of PGC-1α expression has shown promising results in alleviating DKD [607]. However, the overexpression of PGC-1α leads to abnormal proliferation of podocytes, leading to albuminuria and glomerulosclerosis [605]. CKD differs from acute injury in that cytokine levels are fully activated and persist for a long time. Mitochondrial biogenesis plays a crucial role in maintaining MQC by generating new mitochondria that replace damaged ones. Conversely, the inhibition of mitochondrial formation directs cellular fate towards death.

Increased mitochondrial fragmentation has been demonstrated in renal tubular cells and podocytes of both experimental models and patients with DKD [608, 609]. Phosphorylation of the DRP1 protein serine 600 (S600) promotes mitochondrial fission, while specific mutation of S600 to alanine reduces mitochondrial fission and ameliorates kidney damage in DKD mice [610]. In addition, the podocyte-specific knockdown of DRP1 in the DKD mouse model improved its mitochondrial structure function and adaptability [611], and pharmacological inhibition of DRP1 in podocytes reduced mitochondrial fission to stabilize mitochondria thereby improving DKD [611, 612]. Enhanced mitochondrial fission was detected in renal tubules of unilateral ureteral obstruction (UUO)-induced CKD mice [613], as well as fibroblasts in the kidneys of CKD patients and UUO mice [614]. C/EBP homologous protein (CHOP) may be a positive regulator of mitochondrial fission. In the UUO-induced CKD model using CHOP knockout mice, elevated OPA1 expression was observed along with reduced mitochondrial fragmentation compared with wild-type littermates, indicating reduced renal fibrosis [615]. Increased phosphorylation of DRP1S616 in the kidney of UUO mice stimulates mitochondrial fission and promotes fibroblast activation and proliferation, suggesting a positive role in renal fibrosis [614]. In summary, excessive mitochondrial fragmentation accompanying CKD is mainly caused by an imbalance within the dynamics regulating mitochondria.

Mitochondrial fragmentation, impaired mitophagy, and increased apoptosis were found in the renal tubules of the DKD mouse model as well as in high glucose (HG)-induced HK-2 cells [609, 616]. Similarly, a significant reduction in the expression level of OPTN, an important regulator of phagosome formation, was detected in the kidney and HG-induced tubular epithelial cells of patients with DKD. This decrease showed a negative correlation with the severity of CKD [617]. Cellular aging is an important factor in the progression of DKD, and overexpression of OPTN promotes mitophagy in renal tubular epithelial cells in a DKD model, thereby alleviating cellular aging. Additionally, the pharmacological activation of mitophagy serves a similar function [617]. Furthermore, the damage was exacerbated by the specific absence of the PINK1/Parkin pathway in a mouse model of UUO renal fibrosis [618].

### MQC and digestive system disease

#### I/R-induced liver injury

The occurrence of liver I/R injury is observed in various clinical scenarios, including liver transplantation, trauma, hemorrhagic shock, and liver resections [619]. Despite advancements in techniques for safer liver surgery leading to improved surgical outcomes, liver I/R injury is still the major cause of postoperative liver dysfunction and failure, especially in the context of liver transplantation [620].

Many pieces of evidence confirm the beneficial role of mitochondrial biogenesis in I/R-induced liver injury. The expression of PGC-1α decreased in a mouse model of IR-related liver injury and hypoxia-induced human hepatocytes [621, 622]. Irisin is a positive regulator of mitochondrial biogenesis and can restore PGC-1α expression levels and TFAM protein levels in a mouse model of I/R-induced liver injury, thereby ameliorating liver injury. This effect was also observed in an in vitro hypoxic hepatocyte model [621]. However, Sirt1 inhibition prevented the positive regulation of mitochondrial biogenesis by Nobiletin, suggesting that Sirt1 likely acts as an upstream effector for the increase in PGC-1α and TAFM expression [622]. Cilostazol, a phosphodiesterase inhibitor, has been demonstrated to increase HO-1 expression and enhance mitochondrial biogenesis in a mouse model of I/R-related liver injury and extracted primary hepatocytes, thereby improving mitochondrial function, and reducing liver injury [623]. In contrast, cilostazol’s enhancement of I/R-induced mitochondrial biogenesis in both in vitro and in vivo models was blocked after inhibiting HO-1 or Nrf2 expression [623]. This suggests that mitochondrial biogenesis induced by cilostazol is dependent on HO-1 and Nrf2. The liver is a highly regenerative organ that requires stable mitochondria to maintain its function during early stages of injury as well as meeting high energy demands for cellular regeneration post-injury. Therefore, promoting new mitochondria production is beneficial for liver recovery. Excessive inhibition of mitochondrial fission and/or fusion has detrimental effects on IR-associated liver injury. In I/R-related liver injury, there were significantly higher expression levels observed for DRP1 and FIS1 associated with mitochondrial fission compared with sham group [621], while the expression of MFN1 and MFN2, which are associated with mitochondrial fission, was decreased [624]. Irisin reduces mitochondrial fragmentation by decreasing the expression levels of DRP1 and FIS1 to inhibit mitochondrial fission and thereby ameliorate I/R-related liver injury [621]. In addition, both SUMOylation of DRP1 and phosphorylation at Ser616 were significantly increased in the livers of liver transplant recipients undergoing I/R. These post-translational modifications were designed to recruit DRP1 to mitochondria for mitochondrial fission following I/R injury [625]. Augmenter of liver regeneration (ALR, genetic name, *Gfer*), formerly called hepatic stimulator substance, was originally identified in the liver of weanling rats in 1975 [626]. *ALR* knockout mice with liver injury induced by ischemia–reperfusion injury showed a significant increase in SUMOylation of DRP1, which was similarly manifested in I/R-induced hepatocytes, while ALR overexpression prevented the increase in DRP1 SUMOylation [625]. Moreover, inhibition of ALR expression in hepatocytes prevented the translocation of DRP1 to mitochondria and reduced mitochondrial fragmentation [626]. This suggests that ALR is a negative regulator of mitochondrial fission by inhibiting SUMOylation of DRP1, thereby reducing its translocation to mitochondria improving impaired mitochondrial dynamics, and ultimately preventing IR-related liver injury. In addition, the mitochondrial fusion-associated OPA1 protein is hydrolyzed by OMA1 in a mouse model of I/R-associated liver injury, leading to the inhibition of mitochondrial fusion and loss of mitochondrial membrane potential [627]. In contrast, liver injury caused by I/R induced OPA1 hydrolysis and mitochondrial instability was attenuated in a mouse model of hepatocyte-specific overexpression of human cyclooxygenase-2 (COX-2) [627], indicating that human COX-2 may be a positive regulator of mitochondrial fusion in hepatocytes. Mitochondrial dynamics play an important role in IR-induced liver injury, and excessive activation of mitochondrial fission and impaired mitochondrial fusion negatively impact the maintenance of homeostasis and recovery from liver injury.

**Fig. 7:**
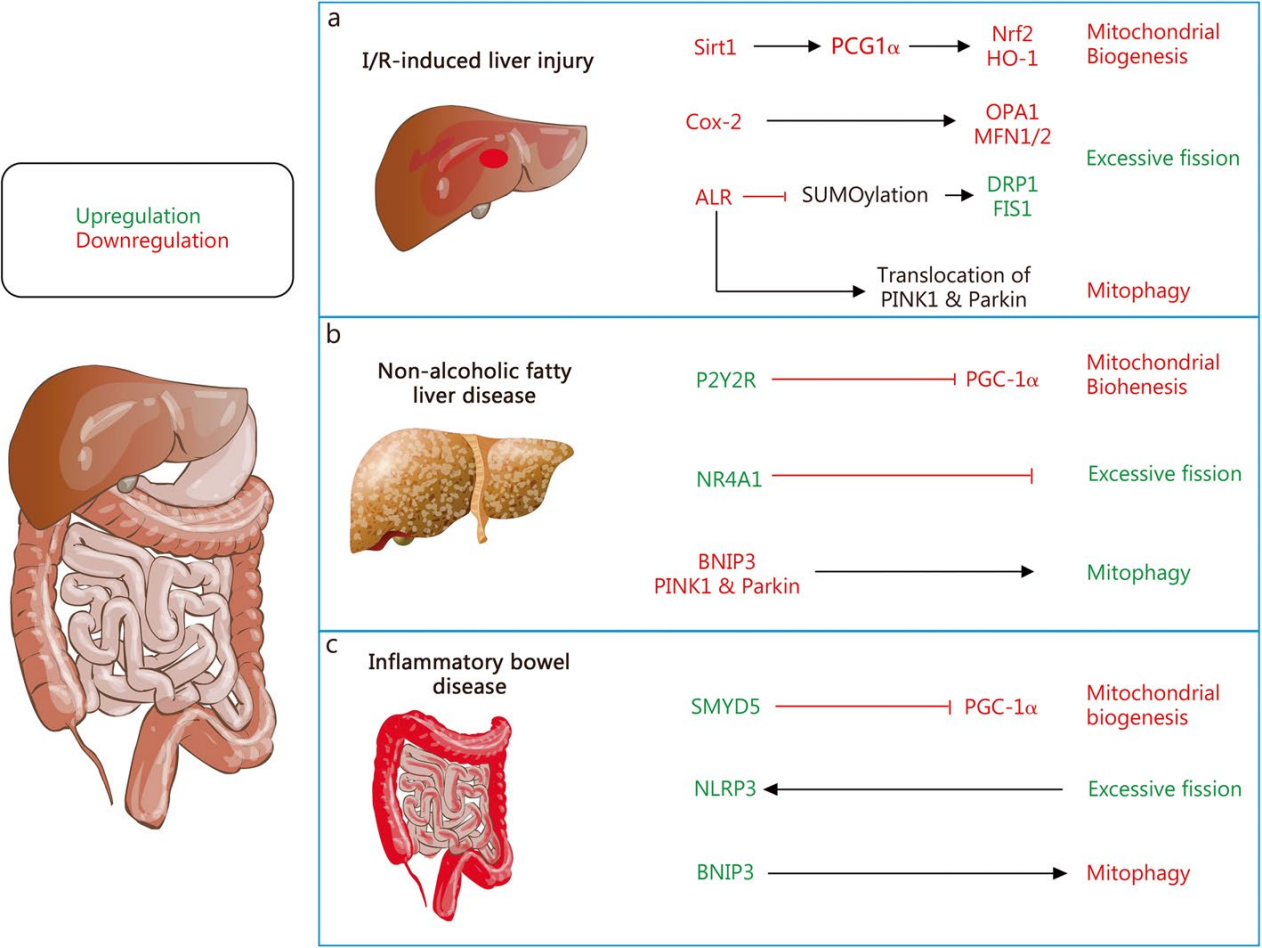
Mitochondrial quality control and digestive system diseases. Various digestive system diseases demonstrate a downregulation of mitochondrial biogenesis, excessive mitochondrial fission, and downregulation of mitophagy within the mitochondrial quality control system. **a** In hepatic ischemia–reperfusion injury, downregulation of sirt1 hinders the downstream mitochondrial biogenesis process, with alterations in Cox-2 and ALR expression levels also involved in the regulation of mitochondrial dynamics disruption. Additionally, ALR is implicated in the regulation of the PINK1 and Parkin-mediated mitochondrial translocation process, leading to impaired mitophagy. **b** In non-alcoholic fatty liver disease, upregulation of P2Y2R affects PGC-1α expression levels, consequently causing inadequate mitochondrial biogenesis. NR4A1 is involved in regulating excessive mitochondrial fission, while mitophagy defect is a notable feature of this disease. **c** In inflammatory bowel disease, insufficient mitochondrial biogenesis is controlled by SMYD5, and notably, excessive mitochondrial fission contributes to the activation of NLRP3 in intestinal inflammation. ALR augmenter of liver regeneration, BNIP3 BCL2 interacting protein 3, Cox-2 cyclooxygenase 2, DRP1 dynamin-related protein 1, FIS1 fission protein 1, MFN mitofusin, HO-1 heme oxygenase 1, I/R ischemia–reperfusion, NLRP3 NLR family pyrin domain containing 3, NR4A1 nuclear receptor subfamily 4 group A member 1, Nrf2 nuclear factor E2-related factor 2, OPA1 optic atrophy 1, PGC-1α PPAR-γ coactivator-1α, P2Y2R P2Y receptor 2, SMYD5 SET and MYND domain 5, Sirt1 sirtuin 1, SUMO small ubiquitin-like modifier

Mitophagy was inhibited in a mouse model of I/R-related liver injury [628]. During hepatic I/R injury, mitophagy facilitated the degradation and recycling of damaged mitochondria, thereby reducing the release of mtDNA while meeting the survival needs of hepatocytes after liver injury. In I/R-induced liver injury, ALR not only enhanced the mitochondrial translocation of PINK1 and Parkin but also induced MFN2 expression [629]. In this process, depletion of MFN2 blocked ALR-induced mitophagy activation, promoted mitochondrial dysfunction, and ultimately increased apoptosis. In addition, mesenchymal stem cells can reduce mtROS overproduction, reduce mitochondrial debris accumulation, restore ATP production, and upregulate the PINK1/Parkin-mediated mitophagy pathway during liver injury. These findings may provide a theoretical basis for exploring novel strategies for the treatment of IR-related liver injury [628] (Fig. 7).

#### Non-alcoholic fatty liver disease (NAFLD)

The prevalence of major factors such as obesity and other components of metabolic syndrome is increasing, leading to a rise in the incidence of NAFLD [630, 631]. In both rodent models and patients with NAFLD, there is a decrease in markers associated with mitochondrial renewal [632], and the hepatic expression of PGC-1α showed a negative correlation with liver fat accumulation and disease severity [633]. Pharmacological activation of downregulated PGC-1α in NAFLD using various products can enhance neo-mitochondrial production, alleviate mitochondrial dysfunction, and improve NAFLD [634]. In addition, estrogen positively regulates the protective effect of PGC-1α in the mouse model of NAFLD [633]. P2Y2R may be a negative regulator of PGC-1α associated with mitochondrial biogenesis. Upregulation of PGC-1α-mediated mitochondrial biogenesis observed in the *P2Y2R* knockout mouse model ameliorates damage and steatosis associated with NAFLD [635]. Therefore, it is essential to induce new mitochondria through PGC-1α-mediated pathways to attenuate NAFLD.

In the NASH model mice fed a Western diet (high in fat, fructose, and cholesterol) for more than 1 month, reduced FIS1 and DRP1 protein levels were observed along with liver inflammation and fibrosis [636]. Reduced mitochondrial fission alleviates hepatic steatosis in a mouse model of NAFLD [637], suggesting the detrimental role played by mitochondrial fission in NAFLD progression and the potential therapeutic target it presents. NR4A1 has been shown to promote mitochondrial fission while inhibiting mitophagy in a mouse model of NAFLD. However, upon melatonin supplementation, the expression of NR4A1 was suppressed, along with reduced downstream mitochondrial fission and restored mitophagy, leading to an improvement of NAFLD [638]. This indicates that pharmacological inhibition of mitochondrial fission may partially contribute to the amelioration of NAFLD along with enhanced mitophagy. In addition, reduced levels of MFN2 expression were detected in steatosis or NASH mouse models [639]. Hepatic-specific knockdown of *MFN2* in mice causes inflammation, triglyceride accumulation, fibrosis, and hepatocellular carcinoma; however, re-expression in a mouse model of NASH ameliorates these conditions [639], highlighting the beneficial role played by MFN2 in NAFLD. Similarly, CXCR3-mediated downregulation of the MFN1 protein leads to mitochondrial dysfunction in a mouse model of NASH [640]. These studies confirm that mitochondrial fission is a detrimental factor in the progression of NAFLD, while mitochondrial fusion serves as a beneficial factor by facilitating the exchange of metabolic substrates and substances between mitochondria.

There is mounting evidence indicating the beneficial role of mitophagy in NAFLD. The expression of BNIP3 is significantly upregulated in the livers of fasted mice fed an HFD [641, 642]. Silencing BNIP3 leads to increased lipid biosynthesis in the liver, accompanied by elevated ATP levels, reduced AMPK activity, and enhanced expression of lipogenic enzymes [643]. Liver mitochondria from BNIP3 null mice exhibited reduced mitochondrial membrane potential, structural abnormalities, and reduced oxygen consumption, and are associated with increased ROS, inflammation, and steatohepatitis-like features [643]. These findings suggest that BNIP3 pathway-mediated mitophagy improves mitochondrial function in a mouse model of NAFLD, and is also linked to lipid synthesis and liver inflammation. The protective effects of the PINK1/Parkin mitophagic pathway were initially observed in studies on alcoholic fatty liver. In a model of alcohol-induced hepatic steatosis, *Parkin* knockout mice showed impaired mitophagy, along with severe swelling and destruction of mitochondria compared with wild-type mice. Additionally, these knockout mice exhibited reduced mitochondrial cristae formation as well as diminished ability for the liver to adapt to alcohol intake [644]. Consistent with this finding, the expression of PINK1 and Parkin was significantly downregulated in an HFD-induced NAFLD mouse model, which was associated with activation of the mitochondria-associated apoptotic pathway and mPTP opening [645]. In addition, pharmacological activation of the PINK1/Parkin mitophagy pathway has been shown to enhance mitochondrial function and alleviate NAFLD in a mouse model of NAFLD [646]. These findings suggest that the PINK1/Parkin and BNIP3 pathways of mitophagy play a beneficial role in the progression of NAFLD by effectively eliminating damaged mitochondria while promoting the generation of new mitochondria through PGC-1α-mediated mechanisms, thereby facilitating hepatocyte mitochondrial renewal and ensuring mitochondrial stability. NAFLD is caused by the persistent accumulation of lipids in hepatocytes and the stabilization of mitochondria can improve the disruption of lipid metabolism and oxidative stress thereby improving NAFLD (Fig. 7).

#### Inflammatory bowel disease (IBD)

The two most prevalent forms of IBD are Crohn’s Disease (CD) and ulcerative colitis (UC). Mitochondrial function in the gastrointestinal epithelium plays a crucial role in maintaining intestinal health, and emerging research suggests a significant association between mitochondrial dysfunction and IBD [647].

PGC-1α exhibited high expression levels in normal intestinal epithelial cells (IECs) [648], whereas its expression was reduced in IECs from patients with IBD [649]. Similarly, repression of mitochondria-related gene expression was found in UC patients, including mitochondrial biogenesis-related PGC-1α [650]. Post-translational modifications were found to regulate PGC-1α in IBD. In colon tissue of IBD patients and mouse models, as well as in human IECs, the expression of SET and MYND domain-containing protein 5 (SMYD5) (a methyltransferase) was upregulated, while PGC-1α expression was downregulated. Knockdown of SMYD5 blocked this process, resulting in upregulation of PGC-1α and improved mitochondrial function and IBD symptoms [651]. Pharmacological activation of PGC-1α also showed a protective effect against IBD by reducing the expression of inflammatory cytokines such as interleukin (IL)-1β, IL-18, and TNF-α in IBD mice, as well as macrophage infiltration in colon tissue. This activation further alleviated mitochondrial dysfunction and oxidative stress, while protecting the histological structure of the colon in an IBD mouse model [652]. These findings suggest an important protective role of mitochondrial biogenesis in the progression of IBD.

The expression of mitochondrial fission-associated DRP1 is upregulated in the intestinal epithelium of IBD [653]. Further, an increased level of mitochondrial fission was detected, which was attenuated by Atractylenolide I [654]. A DRP1 inhibitor suppressed NLRP3, ASC, and caspase-1 upregulated by LPS/DSS-stimulated mice bone marrow-derived macrophages (BMDMs), and the addition of Atractylenolide I showed similar results, while this anti-inflammatory effect was diminished upon overexpression of DRP1 [654]. In summary, Atractylenolide I ameliorates IBD by inhibiting DRP1-mediated activation of mitochondrial fission-induced NLRP3 inflammatory vesicles and suggests a detrimental effect associated with excessive activation of DRP1 in IBD.

There is increasing evidence for the beneficial role of mitophagy in IBD. In UC patients and mouse models, BNIP3 is upregulated and targeted to mitochondria in the intestinal epithelium. Conversely, UC symptoms are more severe in BNIP3 knockout mouse models, accompanied by mitochondrial accumulation [655]. This suggests a protective role of BNIP3-mediated mitophagy in the UC progression. The CD-associated ATG16L1^T300A^ variant leads to impaired mitophagy, mtROS accumulation, and increased IL-1β production as well as pro-inflammatory macrophage polarization with reduced bacterial killing [656, 657]. Andrographolide induces mitophagy in IBD, thereby reducing mitochondrial dysfunction and reversing mitochondrial membrane potential. It also deactivates NLRP3 inflammatory vesicles in macrophages both in vivo and in vitro to ameliorate IBD [658]. In addition, Prohibitin 1 negatively regulates mitophagy in the human colonic epithelium [659]. It suggests that mitophagy is upregulated in IBD patients and plays a beneficial role by clearing damaged mitochondria. Given that mitochondria are often damaged in IBD patients and experimental models, activating mitophagy to clear damaged mitochondria serves as a defense mechanism against the release of mtDNA and sustained production of mtROS for protecting against IBD (Fig. 7).

## Therapies targeting MQC

Due to the critical roles played by mitochondrial function in many biological processes and diseases, targeting the MQC system emerges as an appealing therapeutic strategy. In light of the aforementioned findings, a quantity of MQC-targeted drugs and natural compounds have been widely investigated. This section aims to summarize the actionable mechanisms of these drugs and compounds reported thus far while also envisioning potential future therapeutic directions (Table 1).

**Table 1:** Representative therapies targeting mitochondrial quality control

Target	Intervention	Mechanism and effect	Representative disease
Mitochondrial biogenesis	NAD^+^	Promotes PGC-1α	Obesity Alzheimer’s disease
	Quercetin	Promotes PGC-1α	Osteoarthritis
	Metformin	Activates AMPK and promotes PGC-1α	Pulmonary fibrosis
	Pioglitazone	Defend against oxidative stress and promote mitochondrial biogenesis	Demyelinating diseases
Mitochondrial dynamics	Mdivi-1	Selectively inhibits DRP1 and effectively attenuates mitochondrial fission	Myocardial infarction
			Alzheimer’s disease
			Lung cancer
	P110	Blocks the interaction between DRP1 and FIS1	Parkinson’s disease
			Pulmonary hypertension
	BGP15	Promotes GTPase activity and self-aggregation of OPA1	Heart failure
Mitophagy	Urolithin A	Induces PINK1-dependent mitophagy	Obesity-induced metabolic cardiomyopathy
			Septic myocardial injury
	Spermidine	Induces autophagy	Alzheimer’s disease
			Atherosclerosis
	MitoQ	Induces the transcription of PINK	Intervertebral disc degeneration
	Curcumin	Increases PINK1/Parkin expression	Stress-induced intestinal injury

*AMPK* AMP-activated protein kinase, *DRP1* dynamin-related protein 1, *FIS1* fission protein 1, *Mdivi-1* mitochondrial division inhibitor 1, *PGC-1α* PPAR-γ coactivator-1α, *PINK1* PTEN-induced kinase 1, *NAD*^+^ nicotinamide adenine dinucleotide, *BGP15* N-(2-hydroxy-3-(piperidin-1-yl)propoxy)nicotinimidamide dihydrochloride, *MitoQ* mitoquinone

### Target mitochondrial biogenesis

PGC-1α is widely acknowledged as a key regulator of mitochondrial biogenesis. Upstream, the mTOR, AMPK and Sirtuins pathways play important roles in regulating PGC-1α activity. Downstream, PGC-1α stimulates the expression of Nrf1 and Nrf2, subsequently driving the transcription of the *TFAM* gene [660, 661]. Most of the drugs targeting mitochondrial biogenesis regulate these signaling pathways or their components. Nitric oxide, an endothelial metabolite with significant effects on mitochondrial biology [662], activates PGC-1α through an AMPK-or p53-dependent mechanism to initiate mitochondrial biogenesis [663, 664]. DETA-NO and S-nitroso-N-acetyl-penicillamine could act as NO providers in vitro and in vivo respectively, both of which led to a proliferation of functional mitochondria [665, 666]. Besides, NAD^+^ participated in the process of mitochondrial biogenesis by involving the NAD^+^/Sirt1 axis [667, 668]. In vitro supplementation with the NAD^+^ precursor Nicotinamide riboside activated Sirt1, Sirt3, and mitochondrial biogenesis and improved exercise endurance and insulin sensitivity [669, 670]. Furthermore, NAD^+^ has also been demonstrated to alleviate PD and AD by promoting PGC-1α function, reducing neuroinflammation, apoptosis, and DNA damage [667, 670]. Regarding natural products, there are also several compounds found to regulate mitochondrial biogenesis. Resveratrol, the most extensively studied polyphenolic flavonoid, has been shown to improve mitochondrial function and survival rate in models of cardiovascular and neurodegenerative diseases, stroke, epilepsy, aging, depression, and a variety of cancers, by promoting the expression of PGC-1α, Nrf1 and TFAM [671]. Berberine, a purified extract of the traditional Chinese medicine Coptidis rhizome, has exhibited a great impact on metabolic syndrome, cardiovascular disease, and neurodegenerative diseases through the AMPK/Sirt1/PGC-1α pathway [672, 673]. Quercetin, whose chemical structure is similar to that of resveratrol, not only increases mitochondrial copy number but also enhances mitochondrial membrane potential, mitochondrial oxygen consumption, and ATP levels through the AMPK/Sirt1 signaling. It played a protective role in osteoarthritis rats by reducing oxidative stress [674]. Curcumin which has potentially important biological effects on antioxidant and anti-inflammatory properties, has also been shown to potentially trigger mitochondrial biogenesis in vitro and in vivo experimental models, but the exact mechanism remains to be fully understood [675].

As for synthetic drugs, multiple studies have yielded significant findings. Lipoamide, the neutral amide of lipoic acid, functions as an effective antioxidant and mitochondrial nutrient that can stimulate eNOS expression and cGMP production in a dose-dependent manner, and the eNOS/cGMP/PKG signaling pathway was involved in the stimulation of mitochondrial biogenesis [676]. The AMP analog 5-aminoimidazole-4-carboxamide-1-beta-D-ribotide (AICAR) directly activates AMPK, leading to phosphorylation of PGC-1α and activation of Sirt1 through an AMPK-induced increase in NAD^+^/NADH ratio [677, 678]. However, AICAR’s limited ability to penetrate the restricts its application in the central nervous system [677]. Metformin plays an important role in the regulation of energy metabolism through AMPK activation, which can improve the levels of mitophagy and PGC-1α [493, 679]. While metformin has been reported to improve bleomycin-induced pulmonary fibrosis [680], further clarification is needed regarding its cardioprotective effects due to varying conclusions at different metformin concentrations [681]. Formoterol, a selective β(2)-AR agonist, significantly increased the copy number of mtDNA and upregulated the expression of PGC-1α and several other genes involved in the mitochondrial electron transport chain in an I/R-induced AKI mouse model [594], thereby accelerating the recovery process of podocyte from glomerular injury and improving brain function following traumatic brain injury [682, 683]. Fibrates, small-molecule agonists of the PPAR pathway, have the potential to prevent diabetic peripheral neuropathy by activating the PPARα/AMPK/PGC-1α/eNOS pathway to alleviate nerve and endothelial injury [684]. However, the therapeutic potential of the PPAR hybrid agonist is severely hampered due to hepatotoxicity, with the except for clofibrate [685]. Thiazolidinediones exert a potent effect on improving insulin sensitivity through the PPARγ pathway [686]. Pioglitazone can protect mitochondrial function, defend against oxidative stress promote mitochondrial biogenesis, enhance oligodendrocyte differentiation, and ameliorate the pathological state of demyelinating diseases [687]. Leriglitazone enhances mitochondrial function and increases mitochondrial biogenesis by targeting the PPARγ pathway, offering a potentially effective treatment for Friedreich ataxia [688]. However, its application is limited due to serious safety issues such as life-threatening cardiotoxicity and hepatotoxicity [689]. Rimonabant, a selective pharmacological blocker of cannabinoid type 1 receptor, has also been found to increase mitochondrial biogenesis in WAT by inducing eNOS expression, thereby protecting against HFD-induced adipose accumulation [690].

### Target mitochondrial dynamics

Maintaining a balance between mitochondrial fission and fusion is of crucial importance to cellular energy metabolism and homeostasis, and imbalanced mitochondrial dynamics have been implicated in many human diseases [6, 56]. Recent studies have demonstrated the therapeutic potential of pharmacological interventions that restore balanced mitochondrial dynamics. Inhibition of mitochondrial fission seems to reverse the disease process. Mdivi-1, as the selective chemical inhibitor of DRP1, effectively attenuates mitochondrial fission [691]. The protective effect of mdivi-1 in cardiovascular diseases has been reported. Treatment with mdivi-1 can reduce myocardial injury in a mouse model of MI, attenuate doxorubicin-induced cardiotoxicity, and ameliorate hypertension by inhibiting mitochondrial fission [398, 479, 692]. Besides, mdivi-1 treatment has also been shown beneficial effects on many diseases, including AD, subarachnoid hemorrhage-induced brain injury, autoimmune hepatitis, AKI, CKD, sepsis, and metabolic disease, partly due to the protection of mitochondrial dynamics [489, 595, 611, 693–696]. Furthermore, in cancers such as lung cancer and breast cancer, the expression of DRP1/MFN2 indicates a disruption in mitochondrial function. Treatment with mdivi-1 resulted in reduced proliferation and migration [302, 697]. Lei et al. [698] also demonstrated that mdivi-1 upregulates major histocompatibility complex class I expression in cancer cells by affecting mitochondrial dynamics, thereby diminishing the capability of immune escape and enhancing the efficacy of adoptive T cell therapy. P110 has been reported as another selective inhibitor of excessive mitochondrial fission that blocks the interaction of DRP1 with FIS1 [699]. Inhibiting mitochondrial fragmentation and p53-dependent apoptosis in dopaminergic neuronal cells targeting DRP1, P110 alleviates the PD model both in vivo and in vitro [699, 700]. Moreover, beneficial effects of P110 therapy have been observed in other mouse models such as pulmonary hypertension, IBD, and septic organ injury [701–705].

Pharmacological treatments targeting mitochondrial fusion may be a promising approach for stabilizing mitochondrial dynamics. SAMβA, a novel small peptide that selectively antagonizes the interaction between MFN1 and βIIPKC, demonstrates the ability to reverse excessive mitochondrial fragmentation and ameliorate heart failure in rats [706]. BGP-15 promotes GTPase activity and self-aggregation of OPA1, thereby relieving heart failure and PAH in rats through the maintenance of mitochondrial fusion [707, 708]. Mitochondrial fusion promoter-M1 is considered an inducer of mitochondrial fusion proteins, with its administration separately alleviating prediabetic cardiac I/R injury and diabetic cardiomyopathy in rats [709, 710]. The pharmacological mechanisms of melatonin include modulation of mitophagy and mitochondrial biogenesis. Moreover, melatonin suppresses DRP1‐mediated mitochondrial fission in diabetic cardiomyopathy through Sirt6 as well as the Sirt1/PGC-1α pathway respectively [711, 712]. The function of mitochondrial fusion is destroyed during cerebral I/R injury, but melatonin reverses these conditions via the Yap-OPA1 signaling pathway [713]. In addition, the effects on maintaining mitochondrial dynamics of melatonin have potential benefits in respiratory diseases, including ALI and SARS-CoV-2 infection [579, 714]. Studies have illuminated that mitochondria-targeted antioxidant MitoQ protects against DKD and intervertebral disc degeneration through the regulation of Nrf2-related mitophagy and mitochondrial dynamics balance [616, 715]. In a rat model of myocardial I/R injury, there is a deterioration in the degree of the mitochondria network along with impaired mitophagy; however, this condition can be alleviated by resveratrol treatment [716].

### Target mitophagy

Mitophagy, a type of selective autophagy, plays an essential role in maintaining the homeostasis of mitochondria and cells [717]. There has been an increasing interest in the development of compounds that target mitophagy, particularly focusing on the Parkin/PINK1 pathway and the FUNDC1/BNIP3/NIX pathway. Urolithin A, a natural compound derived from the gut microbiome, induces PINK1-dependent mitophagy and exhibits potential for reversing cognitive deficits and alleviating AD in mice [718]. Similarly, Urolithin A activates mitophagy to improve muscle function and exercise capacity in aged mice. Recently, its clinical efficacy has been demonstrated through a randomized clinical trial containing 66 participants [719, 720]. In addition, treatments of Urolithin A have shown beneficial effects in mouse models of obesity-induced metabolic cardiomyopathy and septic myocardial injury [721, 722]. Similar effects have also been observed with spermidine, another natural inducer of autophagy, in rodent models of AD and PD [723]. Furthermore, aging increases the levels of IL-6 and induces mitochondrial dysfunction, thereby enhancing atherosclerosis. This is accompanied by a protective increase in mitophagy. Spermidine plays a protective role in this condition by upregulating the Parkin level [724]. MitoQ, a mitochondria-targeting antioxidant composed of coenzyme Q10 and triphenyl phosphate cations, exhibits dual effects on mitophagy in different disease models. In a mouse model of diabetic mouse kidney tubular injury, hyperglycemic states downregulate the levels of Parkin and PINK1 and lead to increased oxidative stress, apoptosis, and mitochondrial dysfunction. MitoQ induces the transcription of PINK and restores mitophagy in a Nrf2-dependent manner [616]. Correspondingly, intervertebral disc degeneration involves activation of PINK1/Parkin‐mediated mitophagy along with blockade of mitophagic flux. Treatment with MitoQ further promotes PINK1/Parkin‐mediated mitophagy and restores mitophagy flux [715]. Interestingly, exposure to PM2.5 induces excessive vascular fibrosis through enhanced mitophagy; however, this process can be inhibited by MitoQ via ROS/PINK1/Parkin pathway [238]. Whether these discrepancies could be explained by cell specificity requires further investigation. Metformin, a classical hypoglycemic drug, promotes mitophagy of peripheral blood mononuclear cells through AMPK phosphorylation to relieve chronic inflammation and improve β-cell function in vitro and in vivo. This effect has also been confirmed by a randomized controlled trial [493, 725, 726]. Moreover, studies have revealed that metformin can attenuate subarachnoid hemorrhage-induced early brain injury and diabetes-induced renal tubulointerstitial fibrosis through AMPK-dependent mitophagy [727, 728]. Empagliflozin, another novel anti-diabetes drug, activates mitophagy in endothelial cells via the AMPKα1/ULK1/FUNDC1 pathway to alleviate cardiac microvascular I/R injury [173]. Melatonin has been recognized as a regulator of mitophagy and has been shown to ameliorate myocardial I/R injury in mice by improving protective mitophagy and mitochondrial dynamics via the AMPK/OPA1 signaling pathways [411]. Melatonin is beneficial for diabetic cardiomyopathy in mouse models through increased expression of LC3 II, colocalization of mitochondria and lysosomes, as well as translocation of Parkin [729]. Furthermore, the therapeutic effects of melatonin have also been verified in various diseases, including AD, experimental liver fibrosis, traumatic brain injury, and LPS-induced ALI [579, 730–732].

Some natural compounds can also contribute to the maintenance of mitophagy. Curcumin, a natural polyphenol extracted from Curcuma longa Linn, can enhance intestine barrier function and mitigate oxidative stress-induced intestinal injury by increasing Parkin/PINK1 expression [733]. Quercetin inhibits oxidative stress, and ER stress and restores PINK1/Parkin-related mitophagy through Sirt1/TMBIM6, and eventually ameliorates hypoxia-caused cardiomyocyte injury [734]. It has been reported that quercetin can improve the neurochemical levels and PD disability score in PD rats, partially owing to the regulation of PINK1/Parkin mitophagy [735]. Furthermore, regulating the interplay between NLRP3 inflammasome and mitophagy in microglia appears to be a potential novel therapeutic target for quercetin in neurological and neurodegenerative diseases [736]. Puerarin limits the inflammation vulnerability of human umbilical vein endothelial cells by improving the mitochondrial antioxidant capacity and promoting mitophagy [737]. Furthermore, puerarin pretreatment can modulate mitophagy, mitochondrial dynamics, and inflammation to ameliorate palmitate-induced IR in skeletal muscle cells [738].

### Therapeutic outlook

As a non-pharmacological intervention, lifestyle changes, such as engaging in physical exercise, show promise in improving MQC and maintaining health. Physical exercises can activate protective mechanisms and preserve mitochondrial health [739]. Accumulating evidence suggests that proper physical exercise can induce PINK/Parkin‐mediated mitophagy improve mitochondrial dynamics, and consequently improve cardiac function after MI [740]. However, this protective effect appears to be related to the frequency and duration of exercise, partly due to an increase in ACTH, cortisol, growth hormone levels, and energy expenditure during prolonged exercise sessions [741]. Besides, in the context of exercise, there is a promotion towards a protective phenotype in terms of mitochondrial dynamics and mitophagy observed in the liver and skeletal muscle [645, 742]. A recent study has illuminated that mild ketosis induced by a proper ketone ester diet can improve cardiac function in diabetic mice by limiting oxidative stress and repairing MQC processes [743].

Given the dual origin of mitochondrial proteins from both mitochondrial and nuclear, it is crucial to maintain the complex and adaptable balance of mitochondrial proteome to preserve mitochondrial function [1]. The accumulation of unfolded or misfolded proteins that are over-synthesized within cells can lead to cellular function, which can be mitigated by chaperones (including HSP60, HSP70) and proteases (including ClpP and LON). Targeting mitochondrial proteostasis as an innovative therapeutic regimen is gaining increasing attention. ClpP is upregulated in various cancers, including acute myeloid leukemia, breast cancer, and lung cancer, where its expression correlates with the viability, growth, resistant, and metastasis of malignancies [744]. ONC201 has been identified as one of the ClpP activators that induce degradation of subunits within the respiratory chain complex, therefore leading to the death of tumor cells [745]. Furthermore, treatment with ClpP activator also inhibits inflammatory and ameliorates diet-induced steatohepatitis in mice [746]. However, the mechanisms and underlying substrates of mitochondrial proteostasis have not been fully elucidated, further investigation is required for relevant therapies.

Given the irreversible mitochondrial damage observed in numerous diseases and the limited efficacy of current treatments, mitochondrial transplantation is emerging as a promising therapeutic strategy. This approach involves supplementing impaired mitochondria with healthy autologous mitochondria isolated from normal tissue. These advances have demonstrated positive outcomes in various mammal disease models, including AKI, MI, and ALI, accompanied by enhanced mitochondrial function and minimized adverse reactions [747–750]. Even so, further evaluation is required to assess the effectiveness of existing therapy strategies and ensure the stability of transplantation in subsequent stages.

## Conclusions and perspectives

Over several decades, the relentless efforts by numerous researchers and the rapid development of experimental techniques have led to deep analysis or initial discovery of the diverse functions of mitochondria in biological organisms. Additionally, there has been a more intuitive observation of the dynamic morphology and distribution of mitochondria. Initially recognized as the crucial energy producers in cells, mitochondria now play a pivotal role in regulating cellular functions and phenotypes in response to physiological signals or external stimuli. They not only maintain the basic functions of various organs under physiological conditions but also play a significant role in buffering stimuli and transmitting signals during disease and injury [751]. Therefore, the MQC mechanisms, which ensure the stable function and high plasticity of mitochondria in eukaryotic cells, have gradually gained attention. In this review, we mainly elaborate on the major mechanisms and regulatable targets involved in MQC regulation, including mitochondrial biogenesis, mitochondrial dynamics, and mitophagy, and discuss their roles in physiological activities and various systemic diseases. Furthermore, while summarizing the latest research progress on therapeutic or intervention approaches targeting different aspects of MQC, we also highlight the challenges and gaps in MQC research, providing potential directions for future investigations.

Various key proteins involved in different main aspects of MQC have been identified. Mitochondrial biogenesis ensures the appropriate quantity of mitochondria and flexible expansion in response to increased energy demand. The pivotal regulator for initiating mitochondrial biogenesis is PGC-1α, which controls the synthesis of new mitochondrial proteins and replication of the mitochondrial genome [11]. Additionally, mitochondrial dynamics underscore the highly dynamic nature of mitochondrial function and quantity, involving a delicate balance between mitochondrial fusion and fission. The key regulators responsible for initiating OMM fusion are MFNs, while OPA1 primarily regulates IMM fusion. Conversely, DRP1 and its associated receptors predominantly regulate mitochondrial fission. Currently, these key proteins serve as indicators to monitor the processes of mitochondrial dynamics [58]. During the clearance process of dysfunctional or severely damaged mitochondria, mitophagy is activated. The main pathways include Parkin-mediated mitophagy and non-Parkin-mediated pathways such as BNIP3/BNIP3L and FUNDC1 [131]. Current research on the regulation of MQC primarily focuses on the transcription and post-translational modifications of these key proteins. AMPK, CaMK, and other kinases, as well as deacetylases such as Sirt1 and Sirt3, are among the prominent targets for regulating MQC. Beyond its fundamental roles in metabolism and energy synthesis, MQC is indispensable for regulating calcium release, maintaining redox balance in the cellular environment, and even participating in PCD processes. As a result, MQC has garnered increasing interest among researchers due to its implications in cancer, metabolic diseases, and acute/chronic injuries affecting various parenchymal organs. However, despite these advancements, the dynamic changes of MQC in various diseases have not yet been fully elucidated by existing studies. Moreover, within the same disease models, the direction of mitophagy and dynamics can even exhibit completely opposite effects. Therefore, further exploration is warranted to identify detection indicators or experimental methods that enable objective and dynamic monitoring of MQC alterations during the disease progression. This endeavor will contribute significantly towards comprehensively understanding the physiological and pathological functions of MQC.

With the increasing recognition of the role of MQC in organisms, there is promising research potential for developing therapeutic strategies and drugs to improve or promote specific aspects of MQC. Current approaches to enhance MQC often involve upregulating or inhibiting key regulators to influence the MQC process, but targeted interventions specifically targeting mitochondria have received limited attention. Further development of precise mitochondrial-targeted biomaterials and their application as carriers to modulate MQC processes could provide innovative avenues for research.

In general, the present review emphasizes and summarizes the crucial functions of MQC in human health and disease, highlighting its unshakable position. Additionally, we provide a systematic description of current treatment strategies for MQC, aiming to provide theoretical support and novel perspectives for further elucidating the role of mitochondria in human life activities and disease progression, as well as facilitating the development of targeted drugs to improve MQC in the future.

## Data Availability

Not applicable.

## Abbreviations

AD: Alzheimer’s disease
AKI: Acute kidney injury
ALI: Acute lung injury
ALR: Augmenter of liver regeneration
ALS: Amyotrophic lateral sclerosis
AMPK: AMP-activated protein kinase
ATP: Adenosine triphosphate
BCL2: B-cell leukemia/lymphoma 2
BNIP3: BCL2 interacting protein 3
BNIP3L: BCL2 interacting protein 3 like
CaMK: Calcium/calmodulin-dependent protein kinase
CD: Crohn’s Disease
CDK1: Cyclin B/cyclin-dependent kinase 1
CKD: Chronic kidney disease
COPD: Chronic obstructive pulmonary disease
CREB: CAMP-response element-binding protein
CSCs: Cancer stem cells
DKD: Diabetic kidney disease
DM: Diabetes mellitus
DRP1: Dynamin-related protein 1
ER: Endoplasmic reticulum
ERK: Extracellular regulated protein kinase
ERRs: Estrogen-related receptors
ETC: Electron transport chain
FAO: Fatty acid oxidation
FIS1: Fission protein 1
FOXO3: Forkhead box O3
FUNDC1: FUN14 domain containing 1
H/R: Hypoxia/reoxygenation
HD: Huntington’s disease
HF: Heart failure
HFD: High-fat diet
HIF-1α: Hypoxia inducible factor-1α
HSP: Heat shock protein
I/R: Ischemia/reperfusion
IBD: Inflammatory bowel disease
IECs: Intestinal epithelial cells
IL: Interleukin
IMM: Inner mitochondrial membrane
IPF: Idiopathic pulmonary fibrosis
IR: Insulin resistance
JNK: Jun N-terminal kinase
LIR: LC3-interacting region
LONP1: Lon protease 1
M phase: Mitotic phase
MAPK: Mitogen-activated protein kinases
MCU: Mitochondrial calcium uniporter
mdivi-1: Mitochondrial division inhibitor 1
MDVs: Mitochondria derived vesicles
MFF: Mitochondrial fission factor
MFN: Mitofusin
mHtt: Mutant Huntingtin
MI: Myocardial infarction
MiD49: Mitochondrial dynamics protein of 49 kD
mPTP: Mitochondrial permeability transition pore
MQC: Mitochondrial quality control
mtDNA: Mitochondrial DNA
NAFLD: Non-alcoholic fatty liver disease
NF-κB: Nuclear factor-κB
NK: Natural killer
NR4A1: Nuclear receptor subfamily 4 group A member 1
Nrf2: Nuclear factor E2-related factor 2
OMM: Outer mitochondrial membrane
OPA1: Optic atrophy 1
OPTN: Optineurin
ox-LDL: Oxidized low-density lipoprotein
OXPHOS: Oxidative phosphorylation
PAH: Pulmonary arterial hypertension
PAMSCs: Pulmonary artery smooth muscle cells
PCD: Programmed cell death
PD: Parkinson’s disease
PGAM5: Phosphoglycerate mutase 5
PGC-1α: PPAR-γ coactivator-1α
PINK1: PTEN-induced kinase 1
PPAR: Peroxisome proliferator-activated receptor
ROS: Reactive oxygen species
SENP5: SUMO specific peptidase 5
Sirt1: Sirtuin 1
SUMO: Small ubiquitin-like modifier
T2DM: Type 2 diabetes mellitus
TAMs: Tumor-associated macrophages
TBK1: TANK-binding kinase 1
TCA: Tricarboxylic acid
TDP-43: Transactive response DNA-binding protein-43 kD
TFAM: Mitochondrial transcription factor A
TME: Tumor microenvironment
TNF: Tumor necrosis factor
TORC1: Transducer of Creb-related binding protein 1
UC: Ulcerative Colitis
ULK1: Unc-51 like autophagy activating kinase 1
UPS: Ubiquitin-proteasome system
UUO: Unilateral ureteral obstruction
VDAC: Voltage-dependent anion channel
VSMC: Vascular smooth muscle cell
WAT: White adipose tissue
